# Supplementary material for: De novo generation of multi-target compounds using deep generative chemistry
Source: Nat Commun. 2024 May 6;15:3636. doi: 10.1038/s41467-024-47120-y (PMC11074339; doi:10.1038/s41467-024-47120-y)
Supplement: Supplementary file 1 — Supplementary Information [file 41467_2024_47120_MOESM1_ESM.pdf]

Supplementary Information

De novo generation of multi-target compounds using deep generative chemistry

Munson, Brenton P[1,2]; Chen, Michael[1]; Bogosian, Audrey[1]; Kreisberg, Jason F[1]; Licon, Katherine[1]; Ruben Abagyan[3]; Kuenzi, Brent M[1]; Ideker, Trey[1,2,4*]

[1] Division of Human Genomics and Precision Medicine, Department of Medicine, University of California San Diego, La Jolla, California 92093, USA

[2] Department of Bioengineering, University of California San Diego, La Jolla, California 92093, USA

[3] Skaggs School of Pharmacy and Pharmaceutical Sciences, University of California San Diego, La Jolla, California 92093, USA

[4] Department of Computer Science and Engineering, University of California San Diego, La Jolla, California 92093, USA

* Correspondence to tideker@health.ucsd.edu

Supplementary Figures

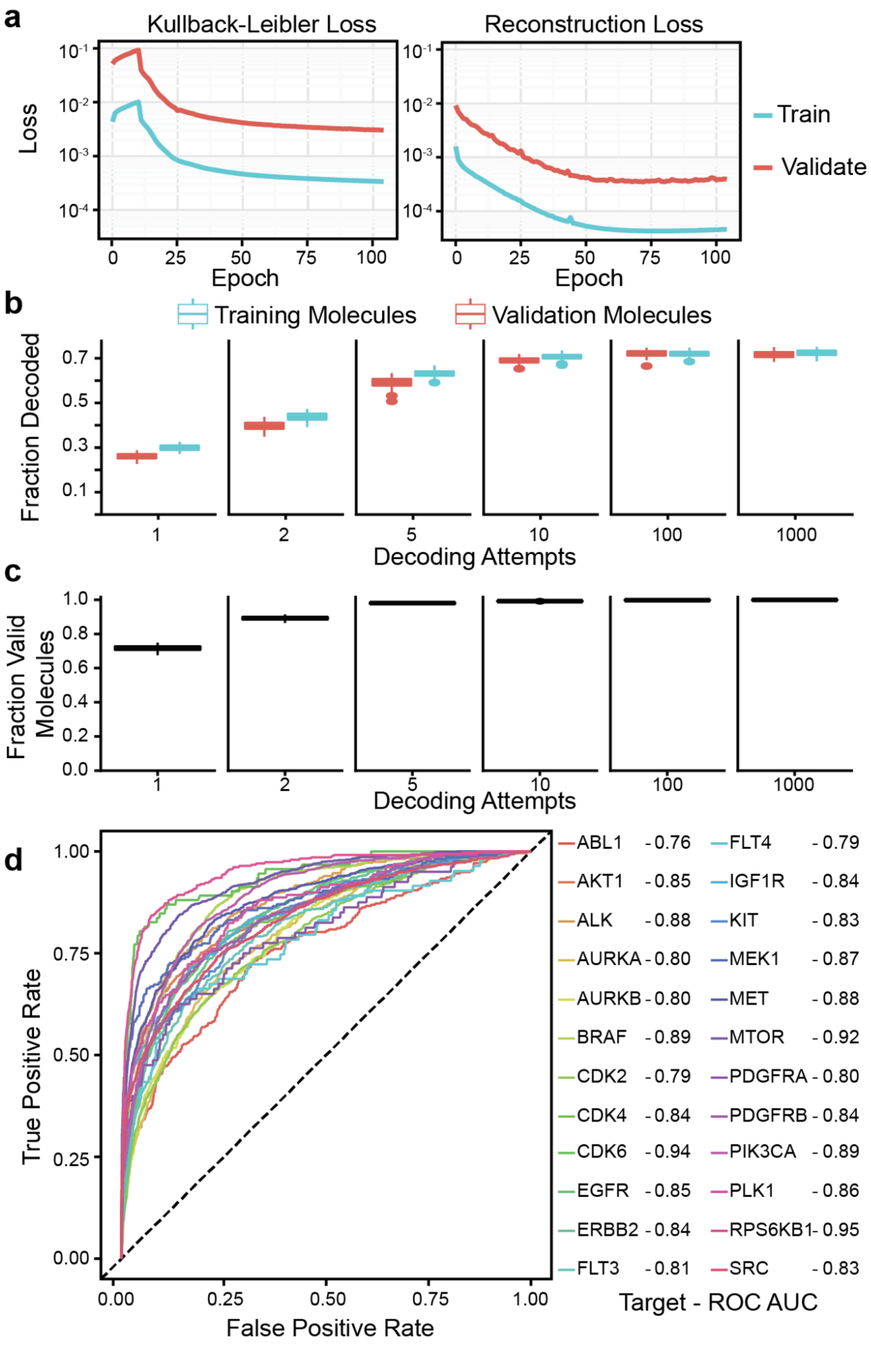

Supplementary Figure 1. Model training performance. a, Training dataset loss and validation dataset loss (cyan vs. red curves) of the POLYGON variational autoencoder over progressive epochs of training. Kullback-Leibler loss (left) quantifies the degree to which the chemical embedding is normally distributed, while the reconstruction loss (right) quantifies the ability of the model to recover compound structures from the embedding. **b**, Fraction of decoded compounds that are close to the corresponding input SMILES string (Levenshtein edit distance < 5), at different numbers of decoding attempts (n=100 for each decoding attempt / molecule category). Training and validation molecule sets are separated and shown in color. Box plots show the minimum (bottom line), first quartile (bottom of box), median (bold midline of box), third quartile (top of box), maximum (top line), and outliers (points). **c**, Fraction of valid SMILES strings decoded from random coordinates in the chemical embedding, at different numbers of decoding attempts. Box plots show are in the same format as in panel (b) (n=100 for each decoding attempt category). **d**, Receiver-operator characteristic (ROC) for multiclass classification of compounds for 24 individual kinase targets (Linear Discriminant Analysis, LDA). Area under the curve (AUC) is given for each target. Source data are provided as a Source Data file.

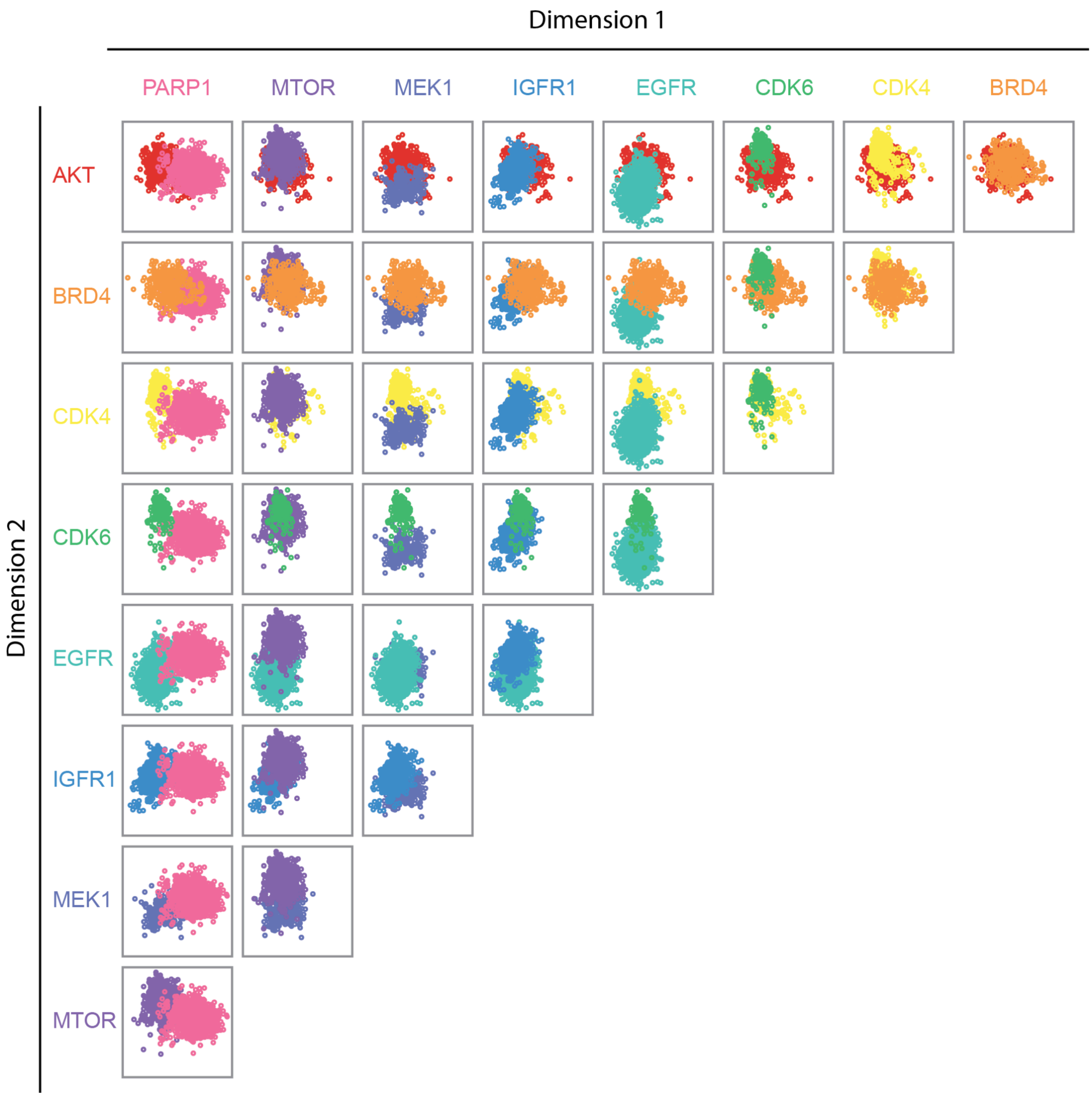

Supplementary Figure 2. Coembeddings in the chemical space for compounds targeting select protein kinases. Points represent compounds in BindingDB, colors label compounds determined to target a given protein target with IC50 < 1 μM. Dimensions 1 and 2 represent the two principal components of the common chemical embedding (**Fig. 2a**). Each panel superposes the compounds binding one target (row) with the compounds binding another (column). Compounds that are recognized by both targets are plotted twice. The number compounds for each protein target range from n=202 to n=1750 (AKT=736, BRD4=397,

CDK4=503, CDK6=202, EGFR=1438, IGF1R=674, MEK1=232, MTOR=1750, PARP1=1618). Source data are provided as a Source Data file.

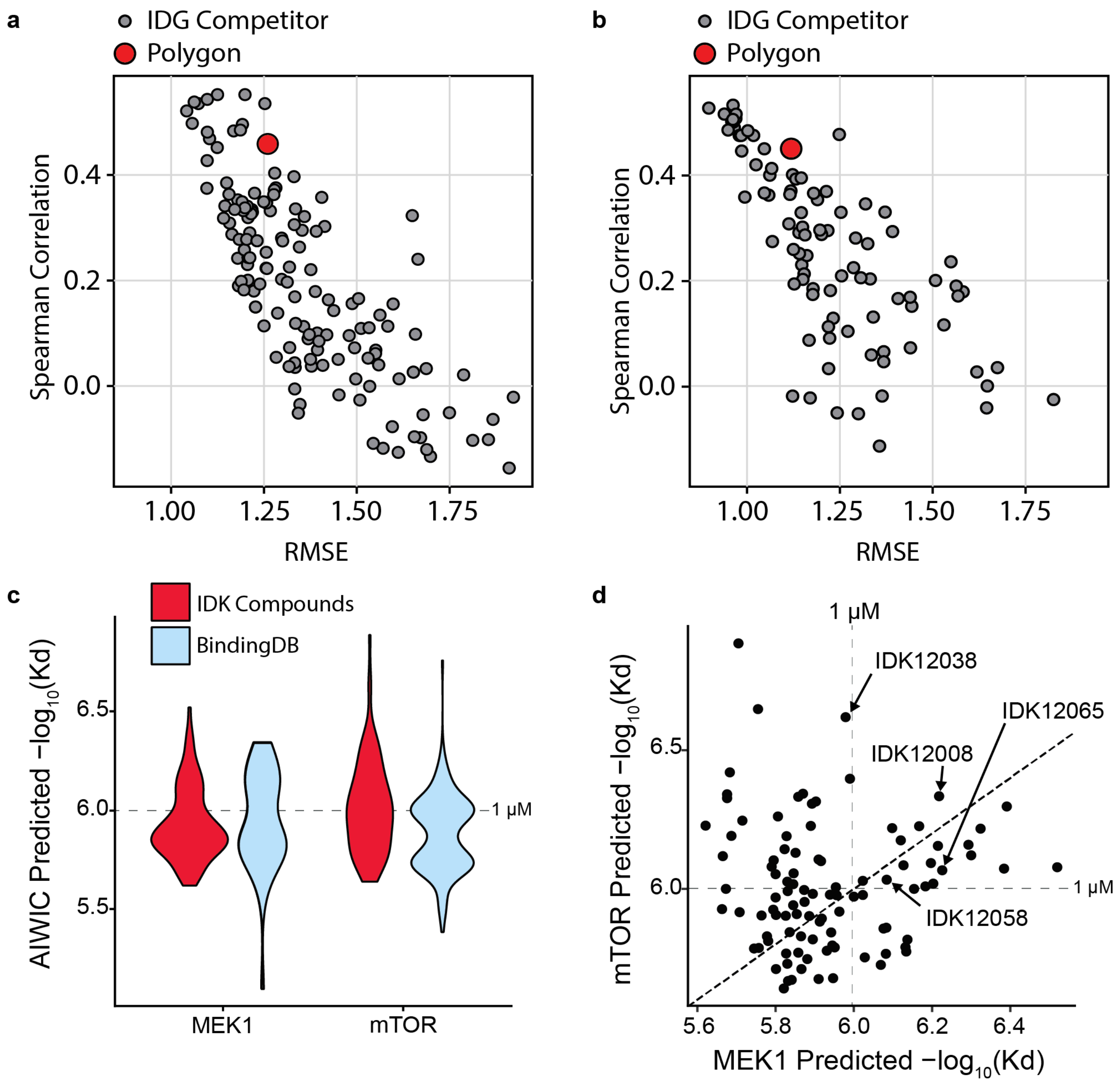

Supplementary Figure 3. Benchmarking POLYGON in IDG-DREAM competition on drug-kinase prediction. a, Performance of POLYGON pIC50 scoring module (red point) in comparison to competing methods (other grey points; method type denoted by shape, selected methods denoted by colors). Performance assessed by Spearman correlation and Root Mean Squared Error (y versus x) in the IDG-DREAM Challenge test data for Round 1, with measurements for 394 kinase-compound pairs across 70 compounds and 192 kinases. Analysis based on Cichońska[12] Fig. 3e. **b,** Like panel **a**, but with performance assessed in IDG-DREAM Challenge test data for Round 2 with measurements for 382 kinase-compound pairs across 24 compounds and 198 kinases. Analysis based on Cichońska[12] Fig. 3f. **c,** Distribution of predicted binding affinities of IDK compounds (red) to MEK1 (left) or mTOR (right), with Kd predictions

made by the best model from the competition, AI Winter is Coming (AIWIC, y axis). For control comparison, predictions also shown for n=200 random compounds sampled from BindingDB (light blue). **d,** Scatterplot of AIWIC-predicted binding affinity of IDK compounds to mTOR versus MEK1; select IDK compounds labeled. Source data are provided as a Source Data file.

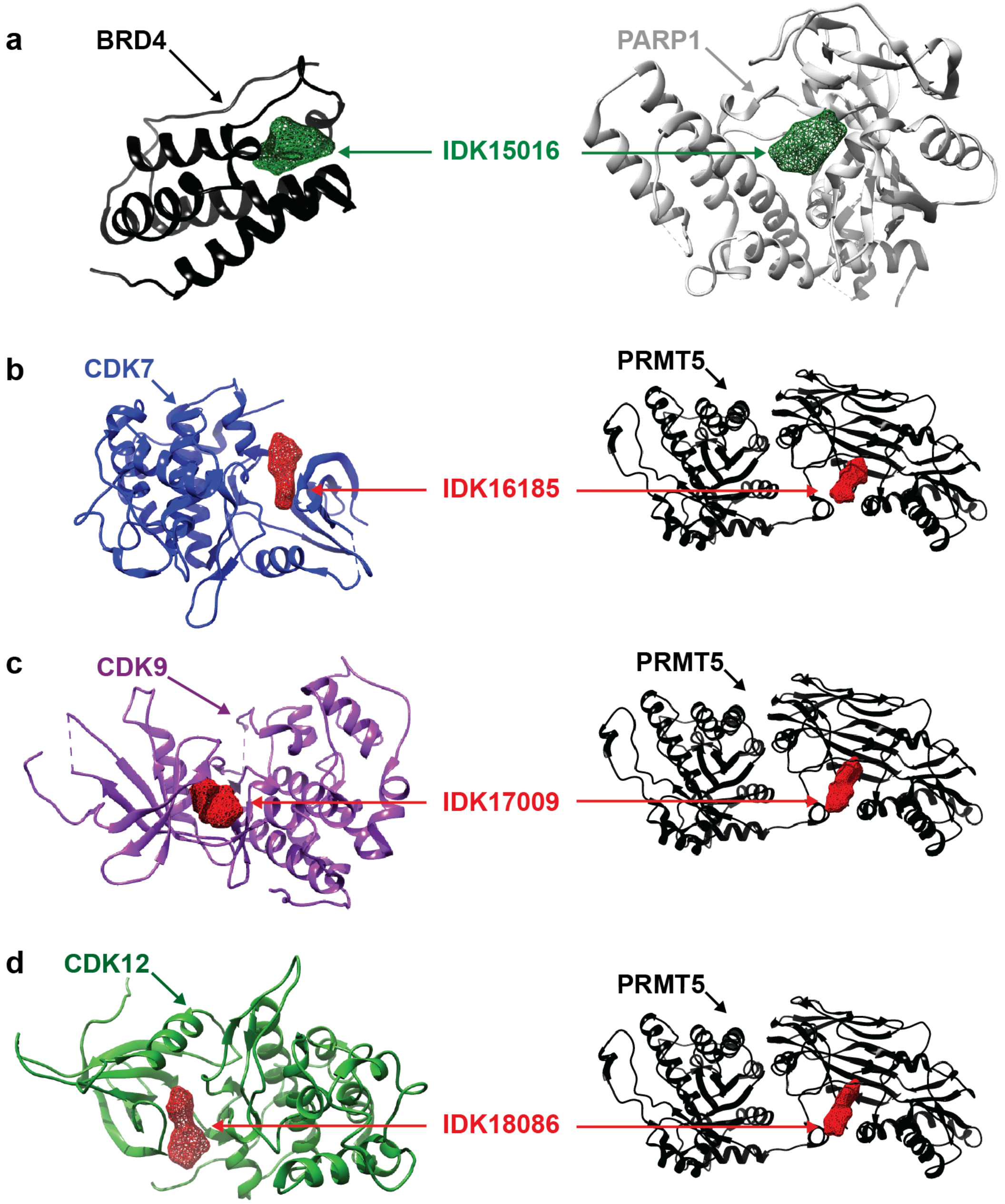

Supplementary Figure 4. Molecular docking simulations of compound designs targeting pairs of protein targets. **a**, Lowest energy docking position of a POLYGON-generated compound IDK15016 (green) in BRD4 (black, left) and PARP1 (grey, right). **b,** Lowest energy docking position of POLYGON-generated compound IDK16185 (red) in CDK7 (blue, left) and PRMT5 (black, right). **c,** Lowest energy docking position of POLYGON-generated compound IDK17009 (red) in CDK9 (green, left) and PRMT5 (black, right) **d,** Lowest energy docking position of POLYGON-generated compound IDK18086 (red) in CDK12 (green, left) and PRMT5 (black, right).

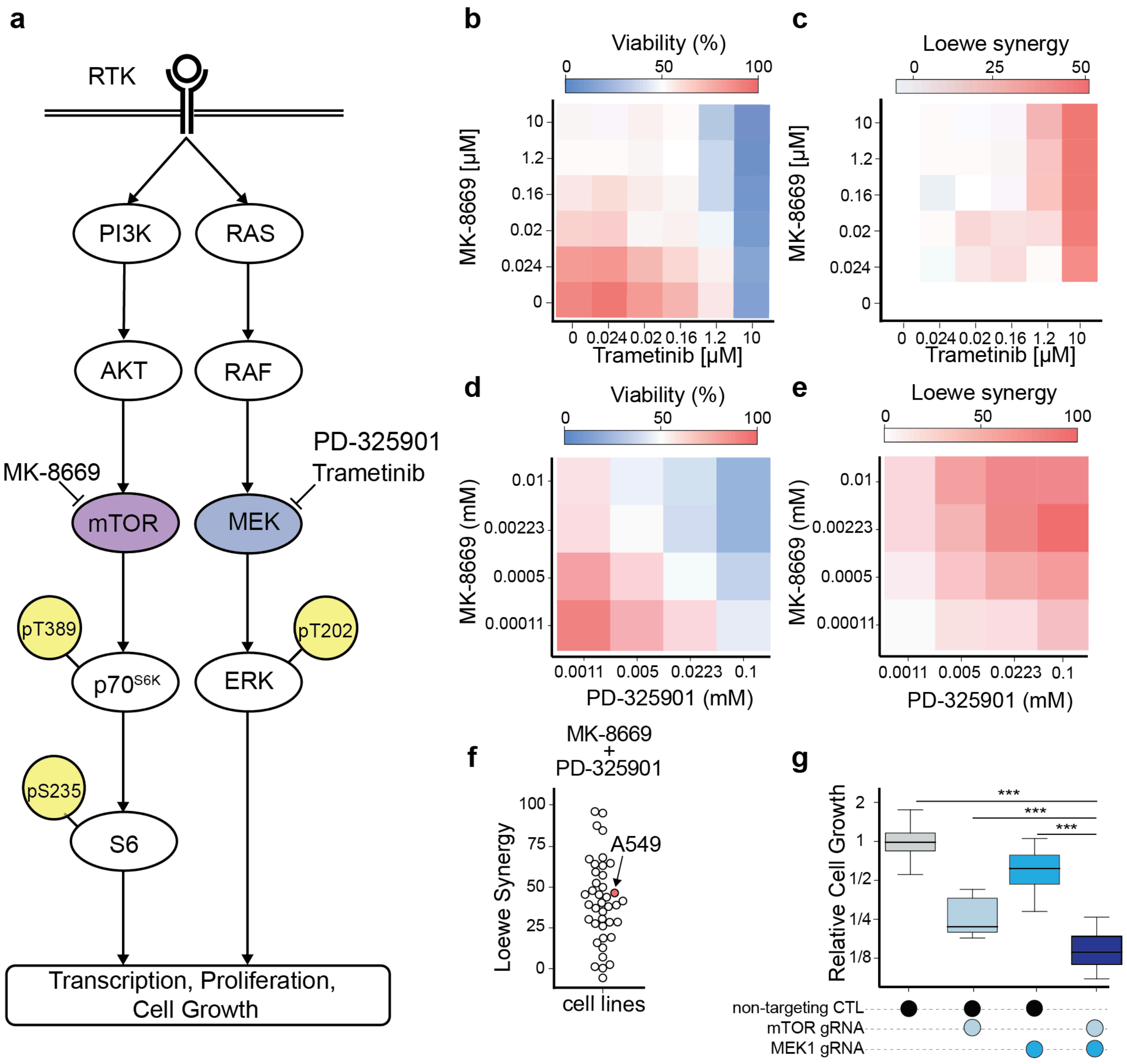

Supplementary Figure 5. Targeting mTOR and MEK1 synthetic lethality by scoring polypharmacology potential of ChemBridge compounds. a, Canonical signaling pathway by which mTOR and MEK kinases become activated following stimulation of receptor tyrosine kinases (RTK). Also shown are the standard inhibitors (MK-8669, trametinib, PD-325901) and phosphorylation readouts (yellow) used in this study. **b,** A459 cell viability relative to DMSO (heatmap) under exposure to combinatorial treatment with canonical inhibitors of mTOR (MK-8669) and MEK1 (trametinib) at indicated doses (y versus x axes). Viability measurements are averages over duplicate readings. **c**, Loewe synergy values computed from cell viability in panel b. **d**, H23 cell viability relative to DMSO (heatmap) under exposure to combinatorial treatment with canonical inhibitors of mTOR (MK-8669) and MEK1 (PD-325901) at indicated doses (y versus x axes), as measured by O'Neil[44]. **e**, Loewe synergy values computed from cell viability in panel d. **f**, Loewe synergy values resulting from combinatorial drug treatment with

inhibitors of mTOR (MK-8669) and MEK (PD-325901) across a panel of 39 cancer cell lines, as measured by O'Neil[44]. Data for the A549 cell line was added from this study (panel b) and is highlighted. **g**, Growth of A549 cells after CRISPR-Cas9 knockout of mTOR and MEK1, individually and in combination, relative to non-targeting control gRNAs. ***$p < 0.001$ by two-sided t-test; exact p-values are provided in the Source Data file. Each gene knockout combination was measured across 9 gRNA pairs in 2 replicate infections. Box plots show the minimum (bottom line), first quartile (bottom of box), median (bold midline of box), third quartile (top of box), and maximum (top line) for each treatment group across all replicates. Source data are provided as a Source Data file.

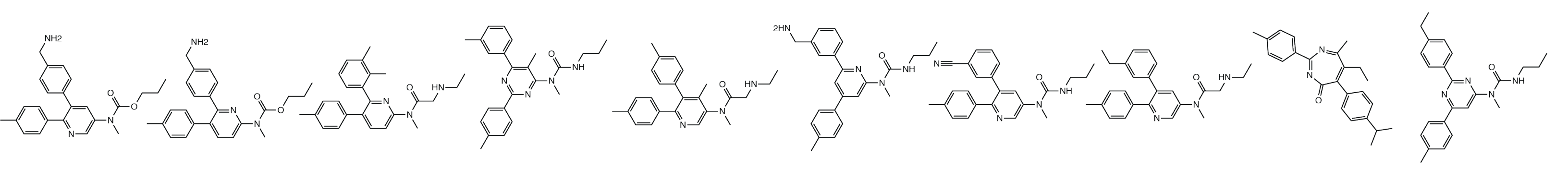

IDK12001 IDK12002 IDK12003 IDK12004 IDK12005 IDK12006 IDK12007 IDK12008 IDK12009 IDK12010

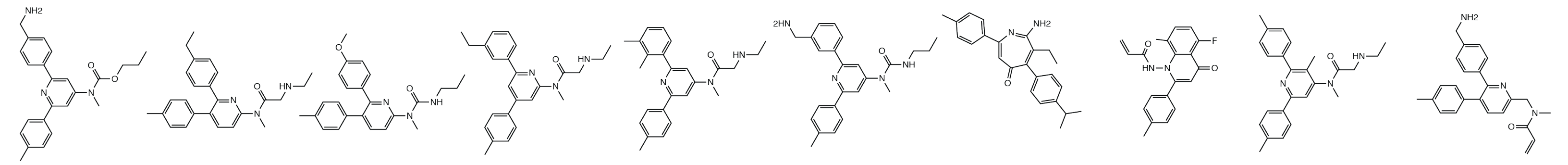

IDK12011 IDK12012 IDK12013 IDK12014 IDK12015 IDK12016 IDK12017 IDK12018 IDK12019 IDK12020

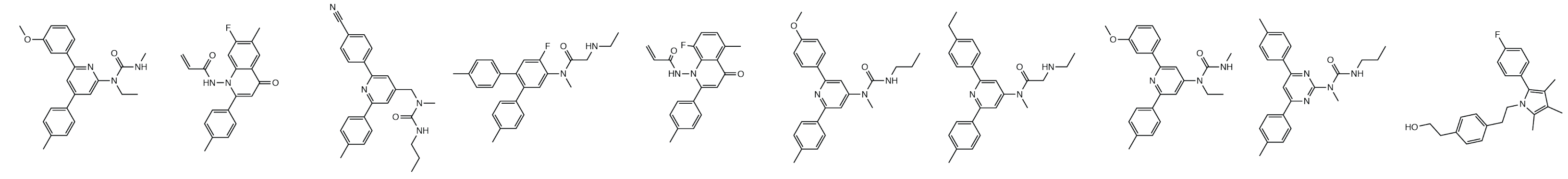

IDK12021 IDK12022 IDK12023 IDK12024 IDK12025 IDK12026 IDK12027 IDK12028 IDK12029 IDK12030

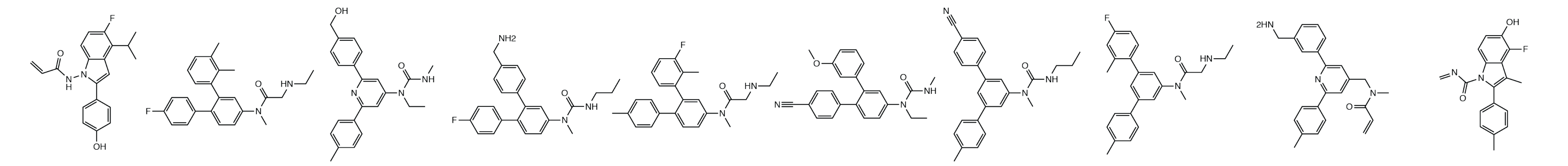

IDK12031 IDK12032 IDK12033 IDK12034 IDK12035 IDK12036 IDK12037 IDK12038 IDK12039 IDK12040

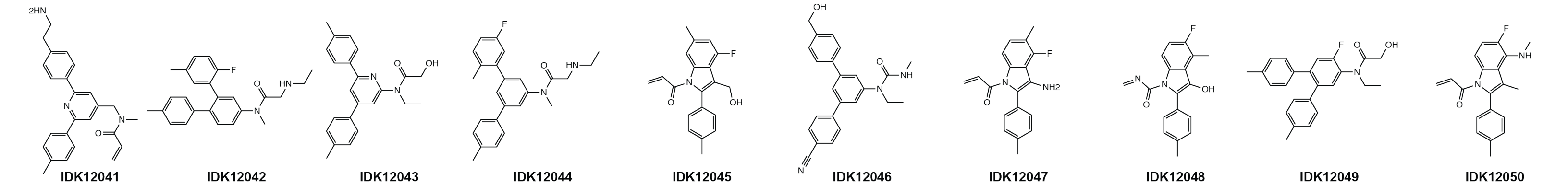

IDK12041 IDK12042 IDK12043 IDK12044 IDK12045 IDK12046 IDK12047 IDK12048 IDK12049 IDK12050

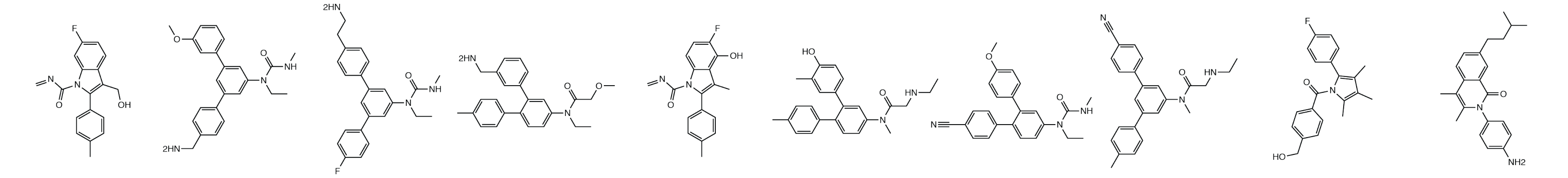

IDK12051 IDK12052 IDK12053 IDK12054 IDK12055 IDK12056 IDK12057 IDK12058 IDK12059 IDK12060

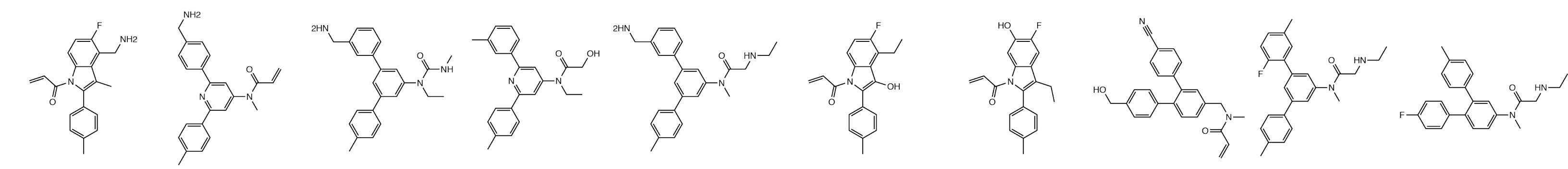

IDK12061 IDK12062 IDK12063 IDK12064 IDK12065 IDK12066 IDK12067 IDK12068 IDK12069 IDK12070

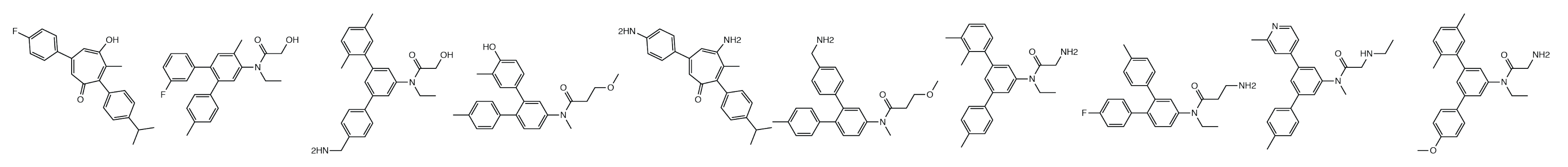

IDK12071 IDK12072 IDK12073 IDK12074 IDK12075 IDK12076 IDK12077 IDK12078 IDK12079 IDK12080

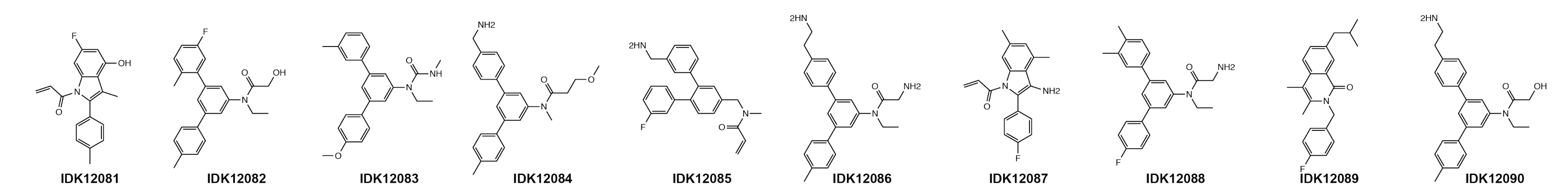

IDK12081 IDK12082 IDK12083 IDK12084 IDK12085 IDK12086 IDK12087 IDK12088 IDK12089 IDK12090

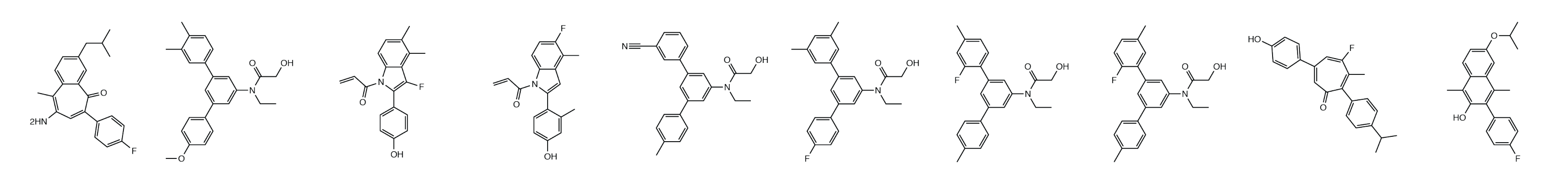

IDK12091 IDK12092 IDK12093 IDK12094 IDK12095 IDK12096 IDK12097 IDK12098 IDK12099 IDK12100

Supplementary Figure 6. Top 100 POLYGON-generated molecules against mTOR/MEK.

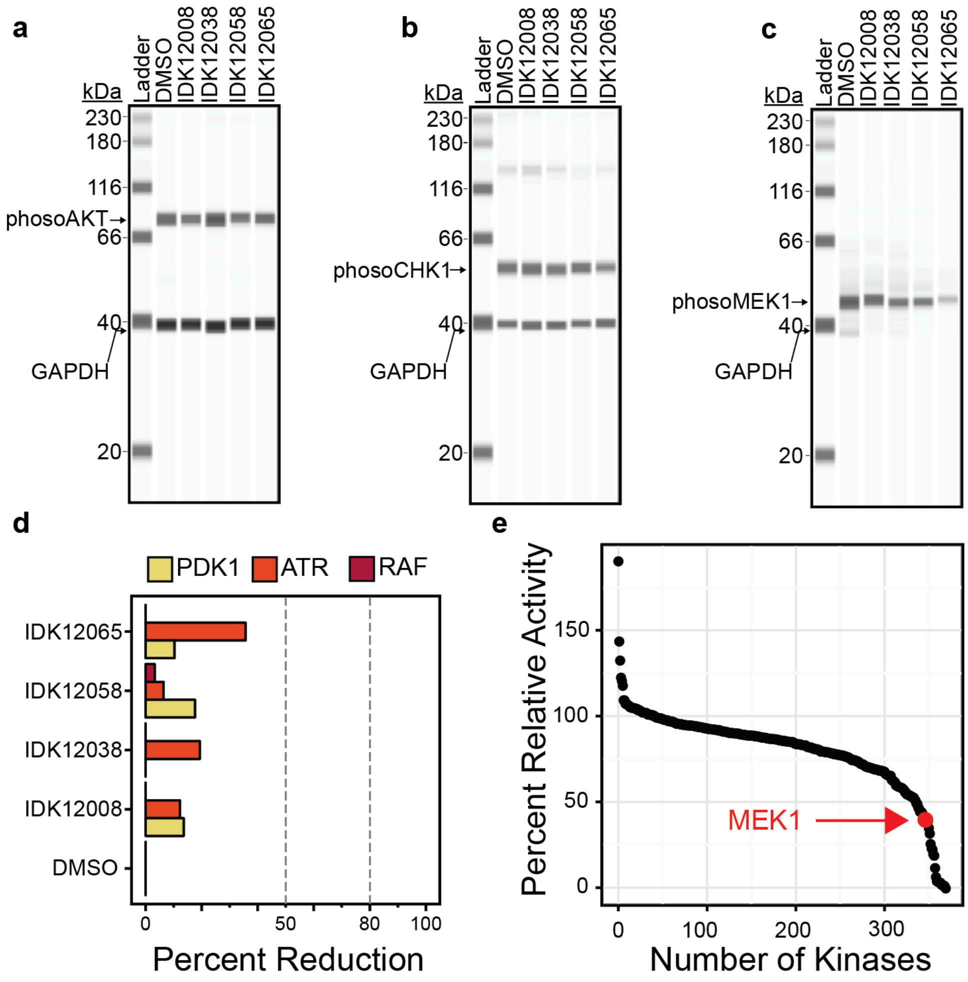

Supplementary Figure 7. Assessment of IDK off-target kinase activity. **a**, PDK1 activity measured by AKT phosphorylation at Thr308 (pAKT, Cell Signaling #9275) upon treatment with 10 μM IDK compounds or DMSO with GAPDH as control. **b**, ATR activity measured by CHK1 phosphorylation at Ser345 (Cell Signaling #2348) upon treatment with 10 μM IDK compounds or DMSO with GAPDH as control. **c**, RAF1 activity measured by MEK1/2 phosphorylation at Ser217/221 (Cell Signaling #9154) upon treatment with 10 μM IDK compounds or DMSO with GAPDH as control. **d**, Percent inhibition of kinases upon exposure to IDK compounds quantified from blots in panels a-c. Activity quantified relative to GAPDH controls and DMSO-treated samples (n=1). **e**, Relative activity of 371 human kinases with a treatment of 10 μM IDK12038, measured with Reaction Biology Wild Type Kinase Panel. MEK1 shown in red. Source data are provided as a Source Data file.

Supplementary Tables

Supplementary Table 1. POLYGON scoring definition for IDK compounds

Score	Goal	Target Mean	Threshold Std Dev	Neighbors	Aggregation
Drug Likeness (QED)	Maximize	0.67	0.1	-	-
Synthetic Accessibility (SA)	Minimize	3.00	0.5	-	-
Ligand Efficiency	Maximize	0.80	0.3	-	-
Latent Distance	Minimize	1.50	0.5	20	Mean

Supplementary Table 2. Key oligonucleotides

gRNA_ID	sgRNA-sequence
MAP2K1-1	TTGGAACAGGACCAACTTGG
MAP2K1-2	TATGGTGCGTTCTACAGCGA
MAP2K1-3	CCATACTTACTCCGCAGAGC
MTOR-1	GCTCCAGCACTATGTCACCA
MTOR-2	CCAGCTCAGATGCCAATGAG
MTOR-3	GAGGAGGTTCCGAAGATAGT
non-targeting-1-0352	GCCATTCTAGTCCCGGCATA
non-targeting-2-0362	GAGTGATGCTTAGACTCCGT
non-targeting-3-0412	GCACGCTGTACAGACGACAA
