# Supplementary material for: De novo generation of multi-target compounds using deep generative chemistry
Source: Nat Commun. 2024 May 6;15:3636. doi: 10.1038/s41467-024-47120-y (PMC11074339; doi:10.1038/s41467-024-47120-y)
Supplement: Supplementary file 3 — Description of Additional Supplementary Files [file 41467_2024_47120_MOESM3_ESM.pdf]

Description of Additional Supplementary Files:

Supplementary Data 1: POLYGON-generated IDK compounds

Supplementary Data 2: Synthetic routes of IDK compound

Supplementary Data 3: 1H NMR results for synthesized IDK compounds
