# Supplementary material for: De novo generation of multi-target compounds using deep generative chemistry
Source: Nat Commun. 2024 May 6;15:3636. doi: 10.1038/s41467-024-47120-y (PMC11074339; doi:10.1038/s41467-024-47120-y)
Supplement: Supplementary file 6 — Supplementary Data 3 [file 41467_2024_47120_MOESM6_ESM.pdf]

Supplementary dataset 3

1H NMR Results for Synthesized IDK compounds

Compound Index

Compound	Page
IDK12001	1
IDK12002	2
IDK12005	3
IDK12007	4
IDK12008	5
IDK12013	6
IDK12024	7
IDK12029	8
IDK12032	9
IDK12034	10
IDK12035	11
IDK12037	12
IDK12038	13
IDK12042	14
IDK12044	15
IDK12049	16
IDK12056	17
IDK12058	18
IDK12065	19
IDK12068	20

Compound	Page
IDK12069	21
IDK12070	22
IDK12074	23
IDK12078	24
IDK12082	25
IDK12084	26
IDK12092	27
IDK12095	28
IDK12097	29
IDK12098	30
IDK12176	31
IDK12195	32

IDK12001

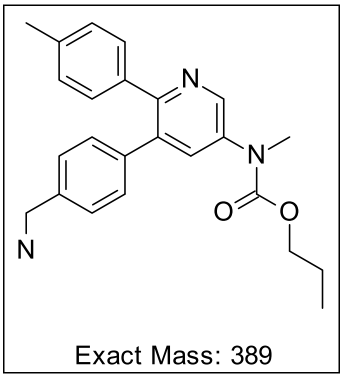

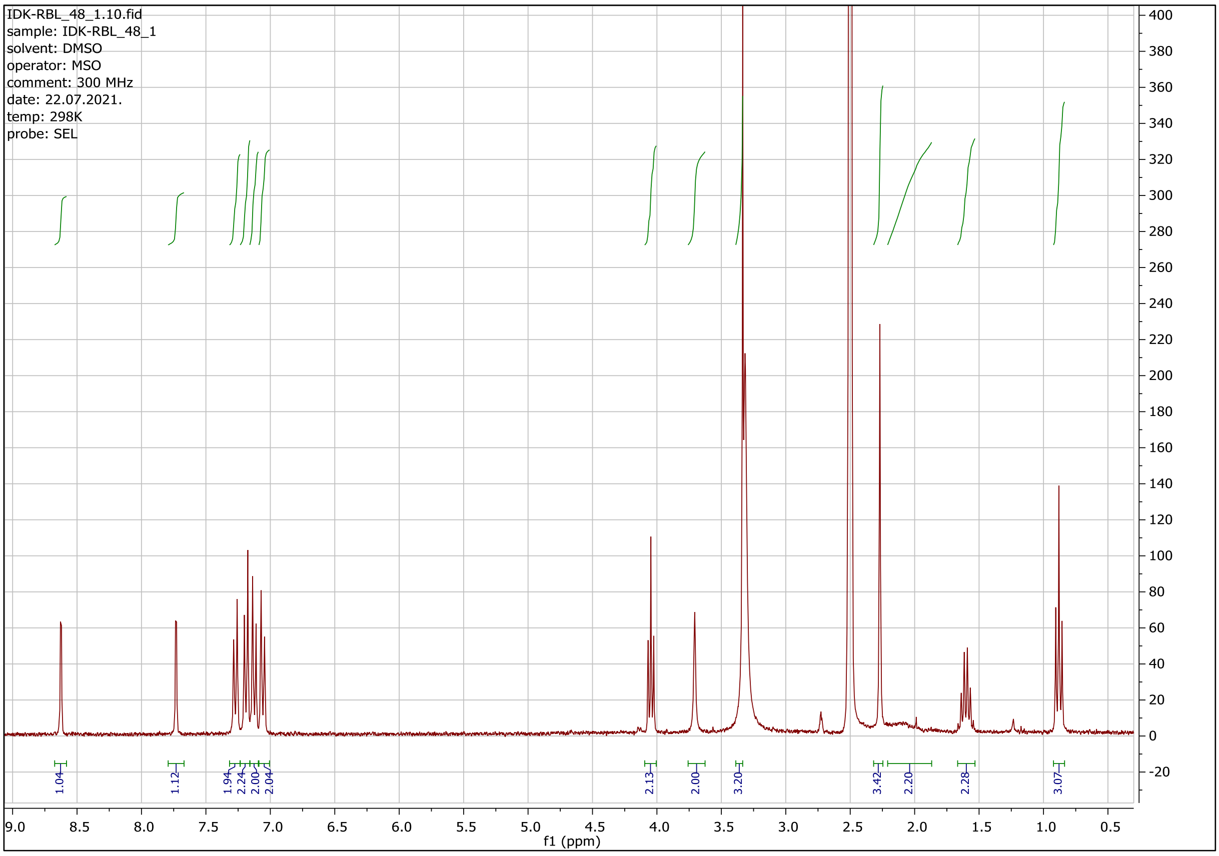

^{1}H NMR (300 MHz, DMSO-d_6) δ 8.62 (d, J = 2.5 Hz, 1H), 7.73 (d, J = 2.5 Hz, 1H), 7.27 (d, J = 7.9 Hz, 2H), 7.23 – 7.16 (m, 2H), 7.16 – 7.09 (m, 2H), 7.06 (d, J = 8.0 Hz, 2H), 4.05 (t, J = 6.6 Hz, 2H), 3.71 (s, 2H), 3.33 (s, 3H), 2.27 (s, 3H), 2.21 – 1.87 (m, 2H), 1.67– 1.53 (m, 2H), 0.88 (t, J = 7.4 Hz, 3H).

IDK12002

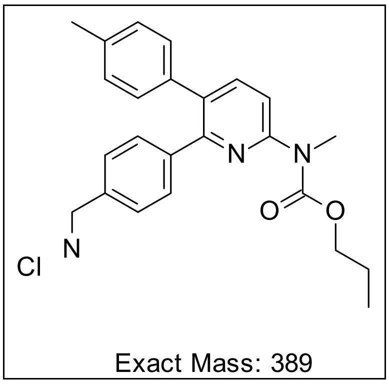

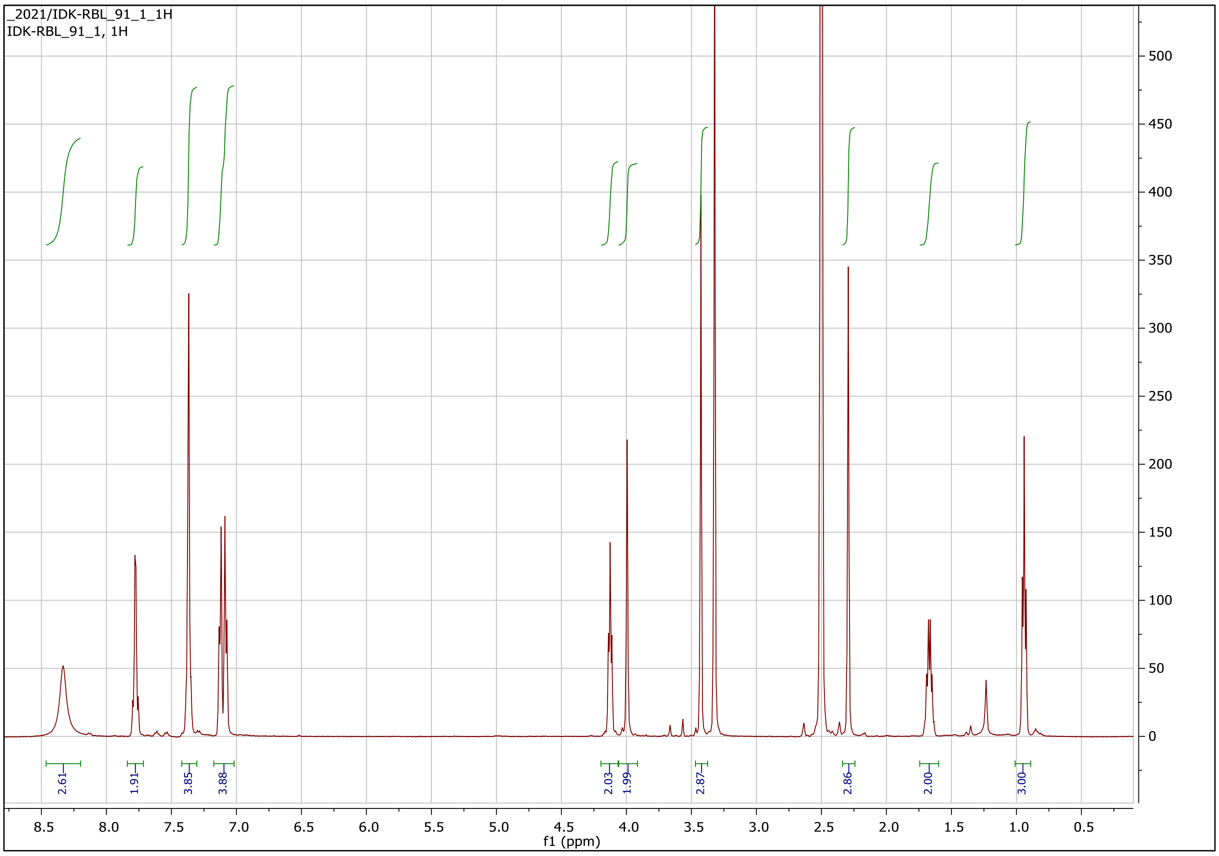

^{1}H NMR (500 MHz, DMSO-d_6) δ 8.33 (br. s, 3H), 7.84 – 7.72 (m, 2H), 7.42 – 7.30 (m, 4H), 7.17 – 7.02 (m, 4H), 4.12 (t, J = 6.5 Hz, 2H), 3.99 (br. s, 2H), 3.43 (s, 3H), 2.29 (s, 3H), 1.74 – 1.60 (m, 2H), 0.94 (t, J = 7.4 Hz, 3H).

Note: the sample contains some grease

IDK12005

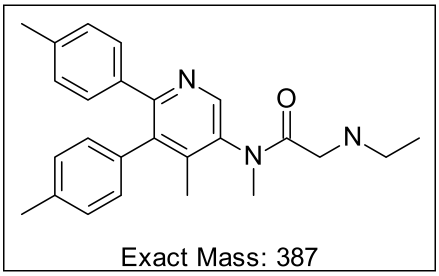

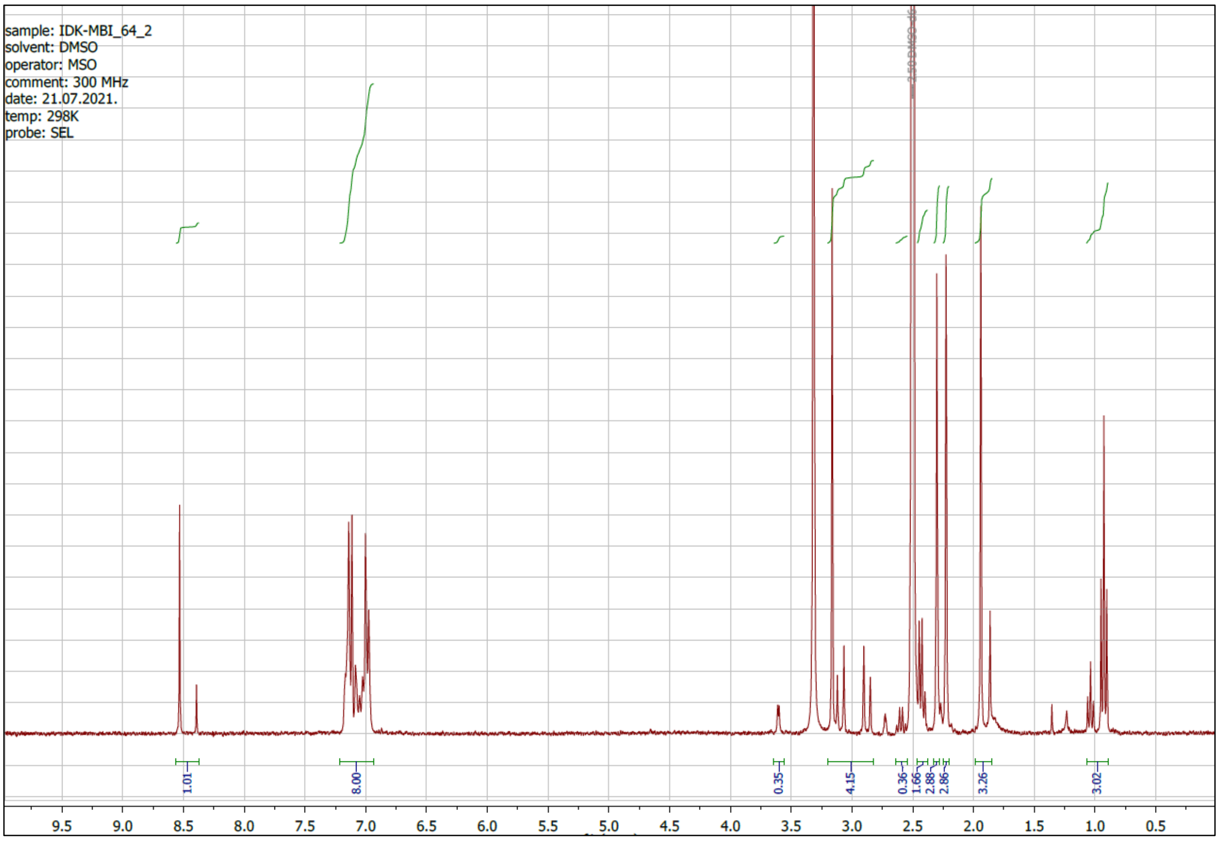

^{1}H NMR (300 MHz, DMSO-d_6) δ 8.56 – 8.38 (m, 1H), 7.21 – 6.94 (m, 8H), 3.20 – 2.82 (m, 4H), 2.45 – 2.38 (m, 2H), 2.30 (s, 3H), 2.23 (s, 3H), 1.99 – 1.85 (m, 3H), 1.07 – 0.89 (m, 3H). ca. 4:1 mixture of rotamers

IDK12007

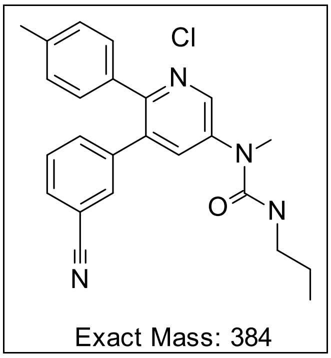

melting range: 183-186 °C

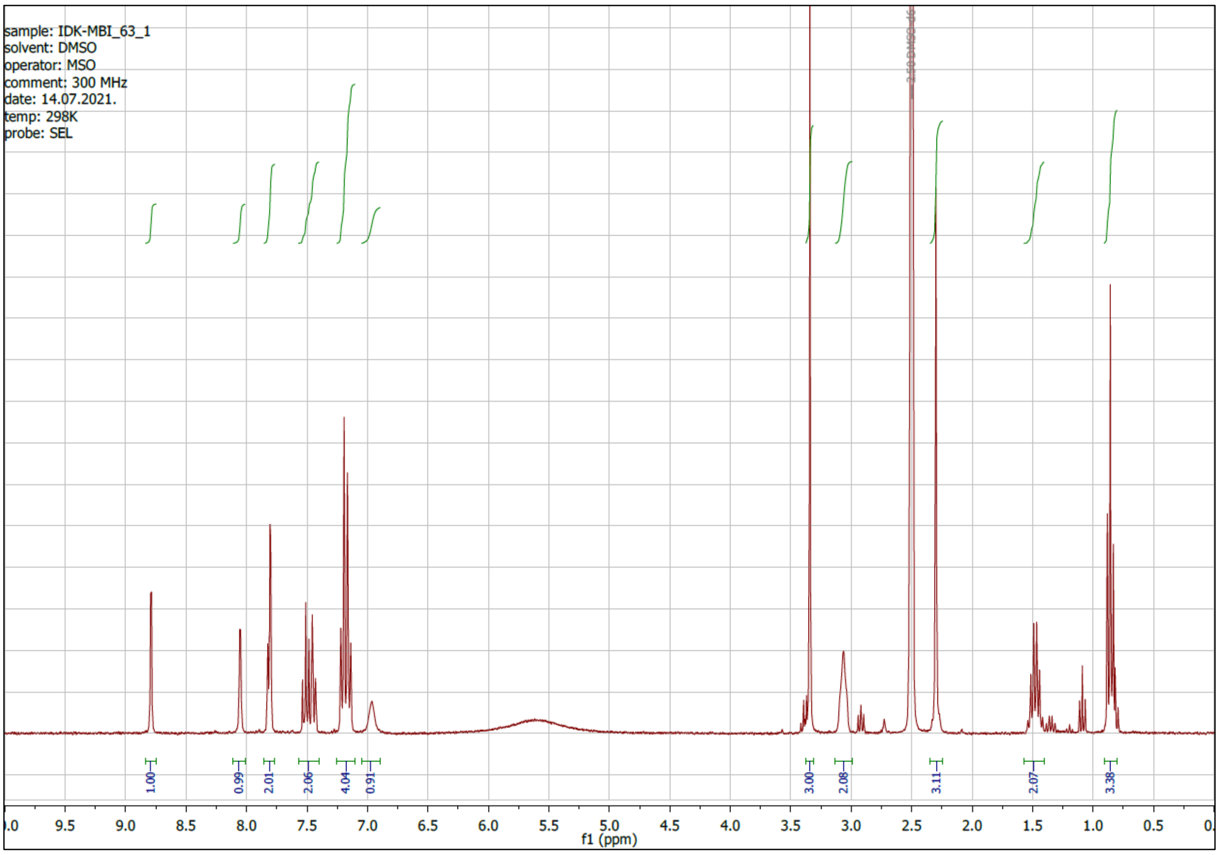

^{1}H NMR (300 MHz, DMSO-d_6) δ 8.79 (d, *J* = 2.5 Hz, 1H), 8.05 (d, *J* = 2.5 Hz, 1H), 7.86 – 7.77 (m, 2H), 7.57 – 7.40 (m, 2H), 7.26 – 7.11 (m, 4H), 6.97 (br.s, 1H), 3.34 (s, 3H), 3.05 (d, *J* = 8.2 Hz, 2H), 2.30 (s, 3H), 1.57 – 1.41 (m, 2H), 0.86 (t, *J* = 7.4 Hz, 3H). Contains 1 w/w% Et_2O.

IDK12008

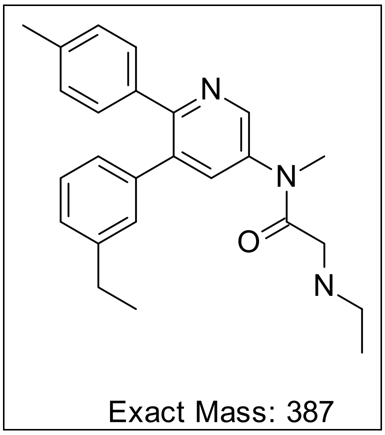

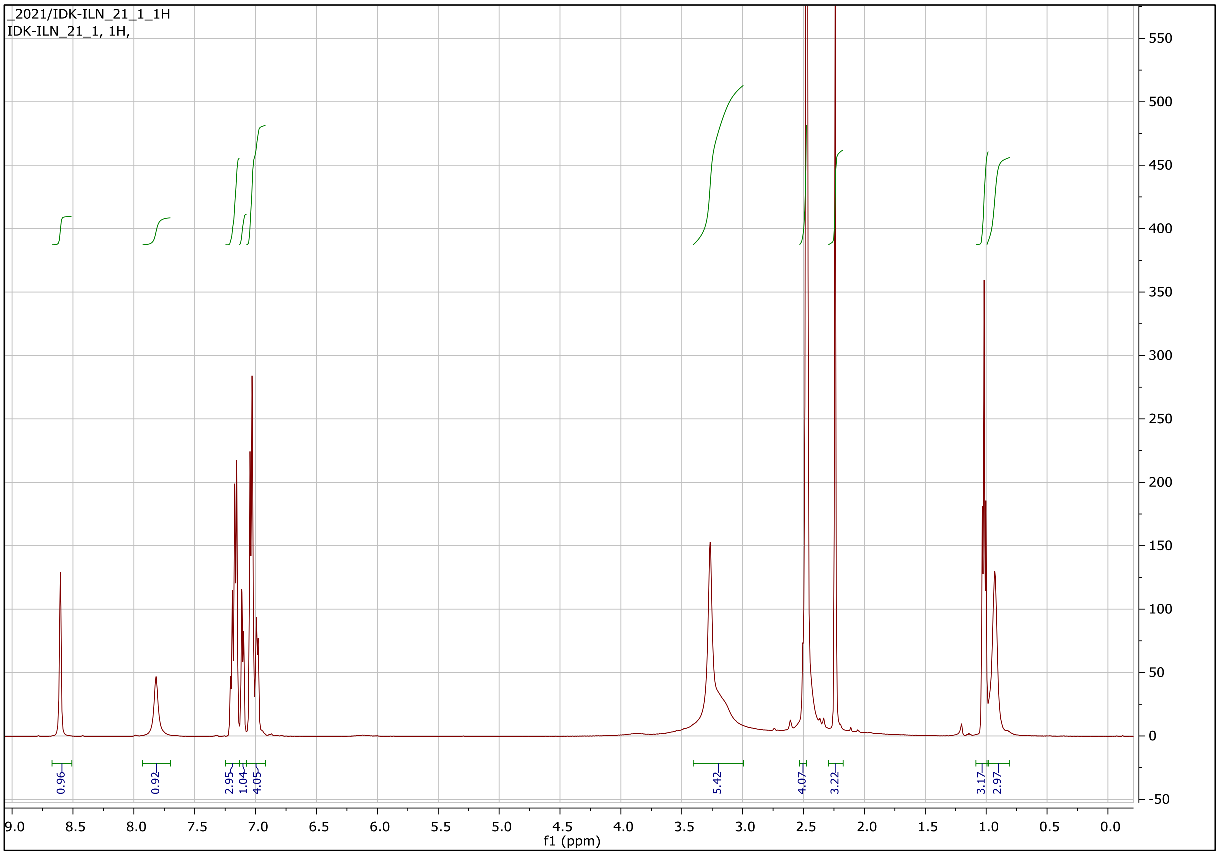

^{1}H NMR (500 MHz, DMSO-d_6) δ 8.60 (s, 1H), 7.82 (br s, 1H), 7.25 – 7.13 (m, 3H), 7.11 (d, J = 7.7 Hz, 1H), 7.07 – 6.92 (m, 4H), 2.24 (s, 3H), 1.02 (t, J = 7.6 Hz, 3H), 0.93 (m, 3H).

Note: 5 protons under water and 4 protons under DMSO

IDK12013

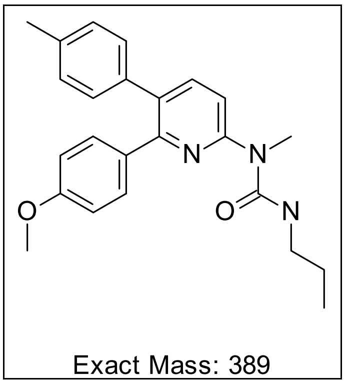

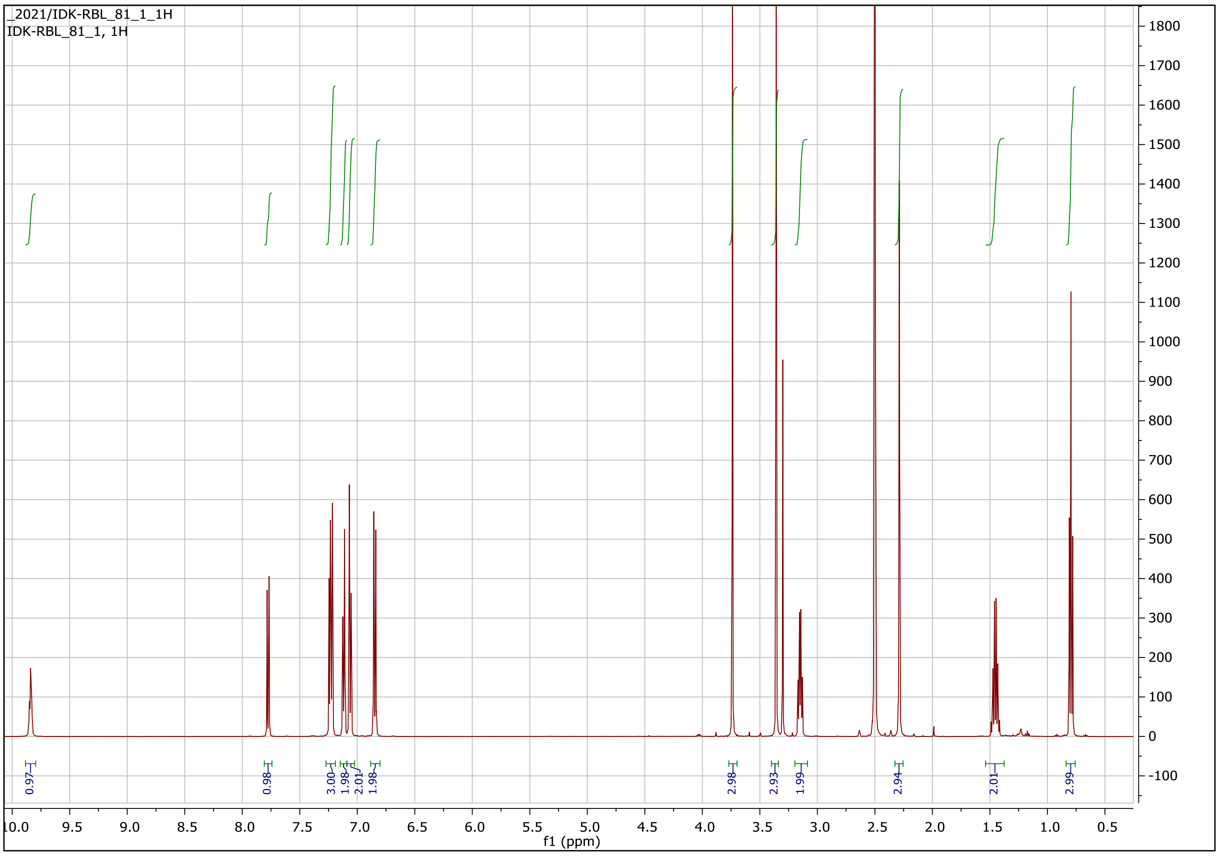

^{1}H NMR (500 MHz, DMSO-d_6) δ 9.84 (t, *J* = 5.3 Hz, 1H), 7.77 (d, *J* = 8.5 Hz, 1H), 7.27 – 7.19 (m, 3H), 7.12 (d, *J* = 7.9 Hz, 2H), 7.06 (d, *J* = 8.2 Hz, 2H), 6.88 – 6.80 (m, 2H), 3.74 (s, 3H), 3.36 (s, 3H), 3.15 (td, *J* = 6.9, 5.2 Hz, 2H), 2.29 (s, 3H), 1.45 (h, *J* = 7.2 Hz, 2H), 0.79 (t, *J* = 7.4 Hz, 3H).

IDK12024

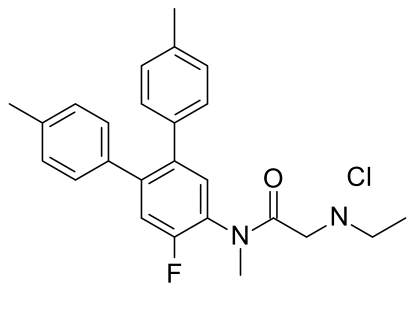

Exact Mass: 390

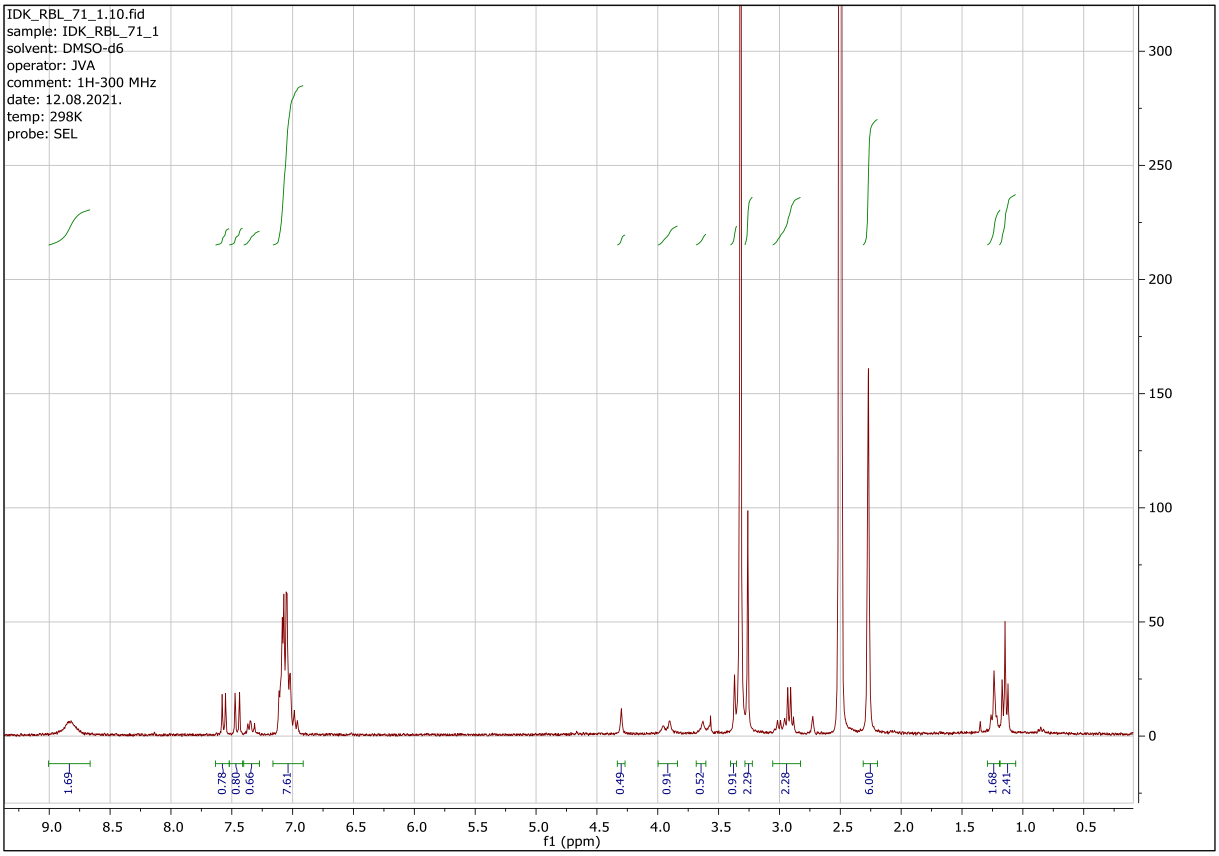

^{1}H NMR (300 MHz, DMSO-d_6) δ 8.83 (br s, 2H), 7.57 (d, *J* = 8.2 Hz, 1H), 7.45 (d, *J* = 11.0 Hz, 1H), 7.40 – 7.27 (m, 1H), 7.16 – 6.91 (m, 7H), 4.00 – 3.84 (m, 1H), 3.68 – 3.59 (m, 1H), 3.26 (s, 3H), 3.06 – 2.83 (m, 2H), 2.31 – 2.22 (m, 6H), 1.15 (t, *J* = 7.2 Hz, 3H).

Mixture of rotamers. Contains less than 2% 1,4-dioxane

IDK12029

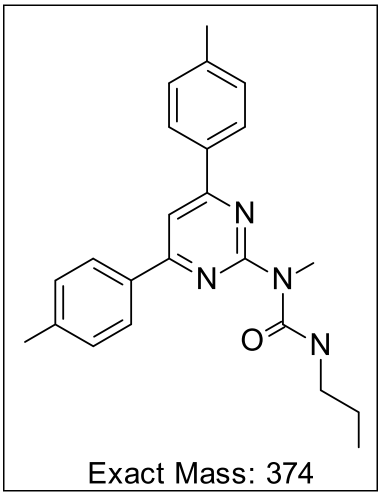

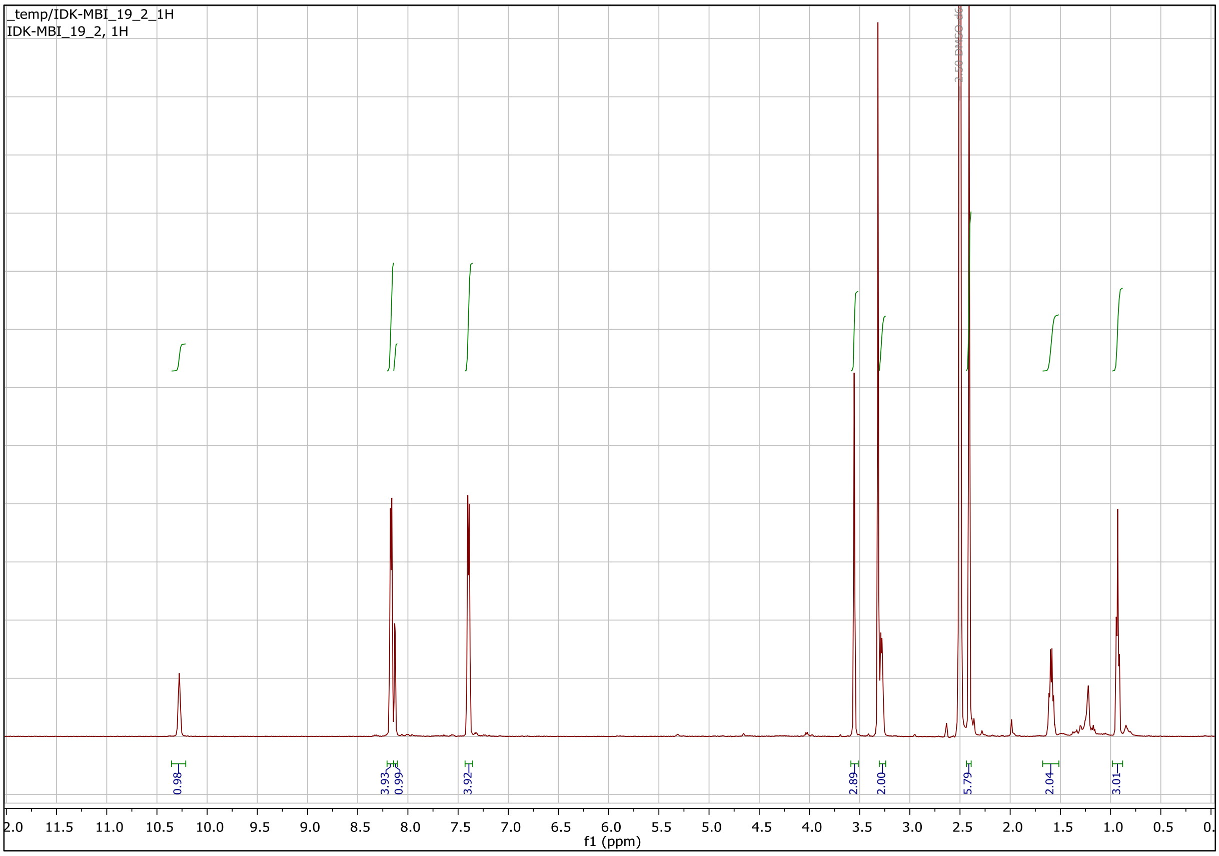

^{1}H NMR (500 MHz, DMSO-d_6) δ 10.28 (t, J = 5.1 Hz, 1H), 8.21 – 8.14 (m, 4H), 8.14 – 8.11 (m, 1H), 7.43 – 7.35 (m, 4H), 3.5 (s, 3H), 3.31 – 3.24 (m, 2H), 2.41 (s, 6H), 1.68 – 1.51 (m, 2H), 0.93 (t, J = 7.4 Hz, 3H).

IDK12032

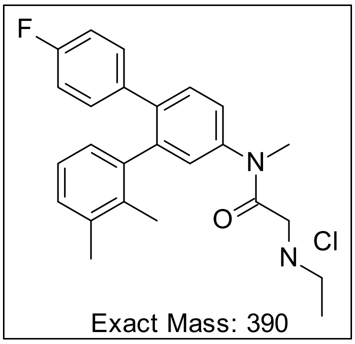

melting range: 181-184 °C

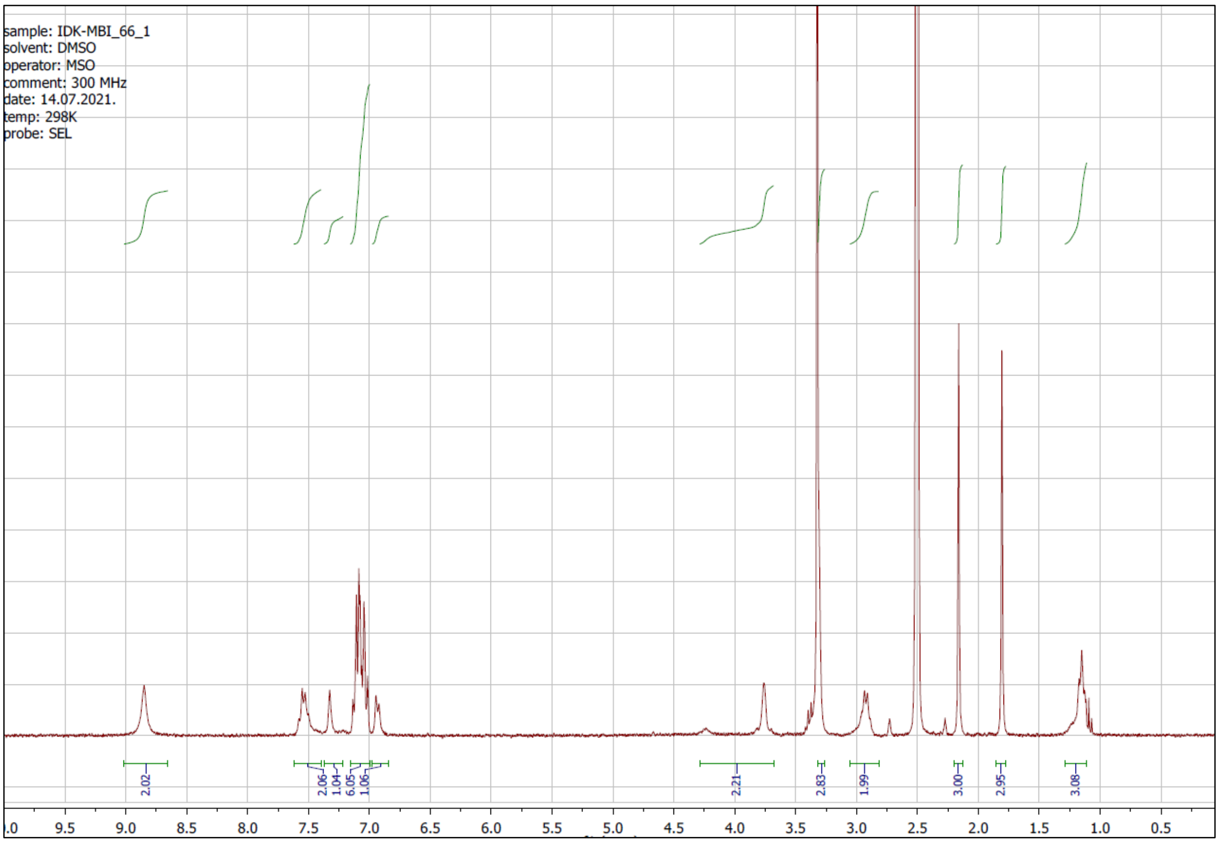

^{1}H NMR (300 MHz, DMSO-d_6) δ 8.85 (br.s, 2H), 7.62 – 7.22 (m, 3H), 7.16 – 7.00 (m, 6H), 6.97 – 6.86 (m, 1H), 4.31 – 3.68 (m, 2H), 3.05 – 2.88 (m, 2H), 2.16 (s, 3H), 1.81 (s, 3H), 1.31 – 1.11 (m, 3H). Contains Et_2O traces

IDK12034

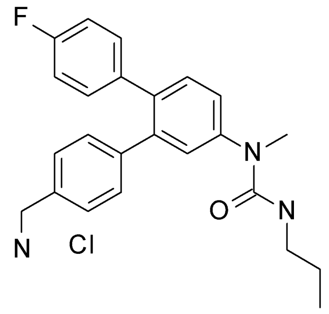

Exact Mass: 391
Exact Mass: 36

melting range: 220 – 223 °C

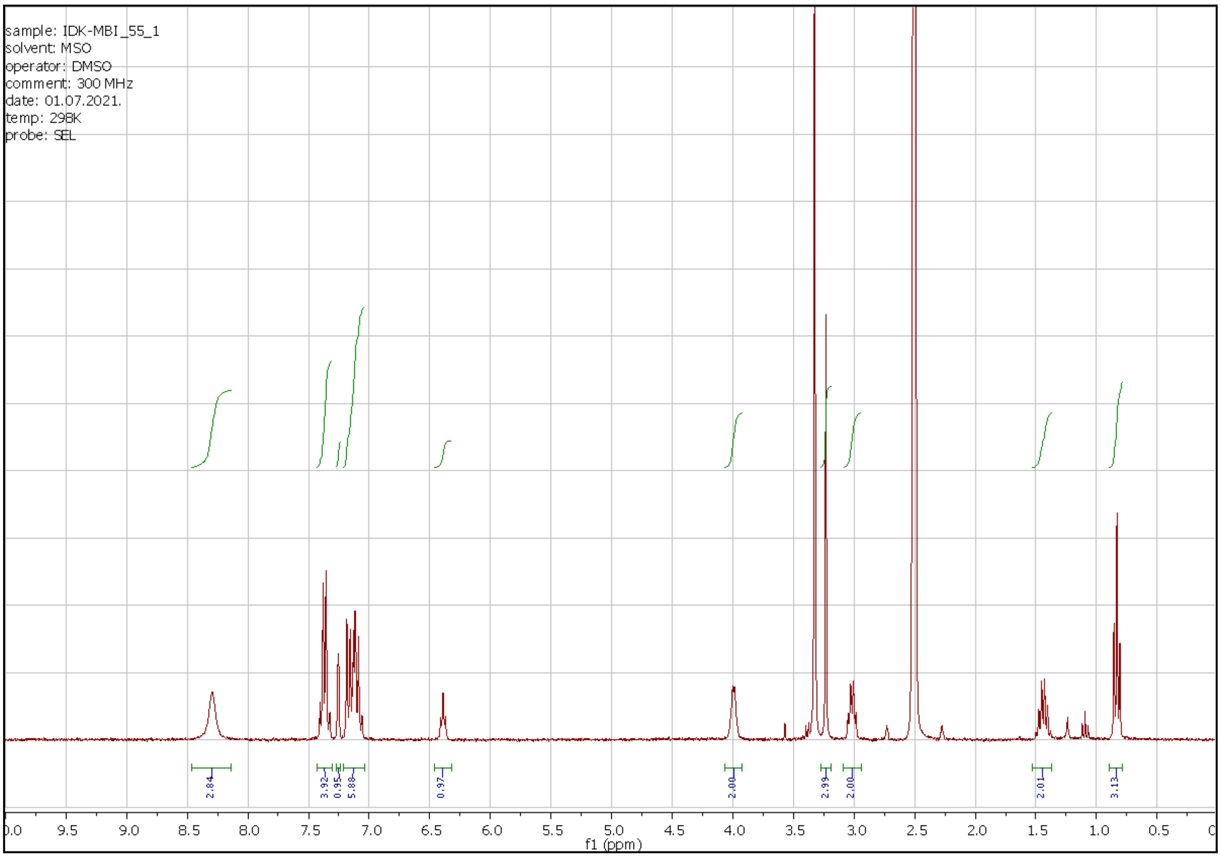

^{1}H NMR (300 MHz, DMSO-d_6) δ 8.29 (s, 3H), 7.43 – 7.31 (m, 4H), 7.25 (d, *J* = 2.2 Hz, 1H), 7.21 – 7.04 (m, 6H), 6.39 (t, *J* = 5.6 Hz, 1H), 4.06 – 3.92 (m, 2H), 3.02 (q, *J* = 6.5 Hz, 2H), 1.52 – 1.37 (m, 2H), 0.83 (t, *J* = 7.4 Hz, 3H). contains 0.9 w/w% Et_2O

IDK12035

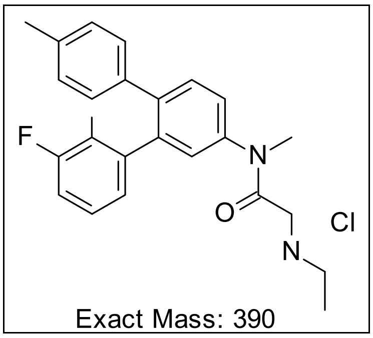

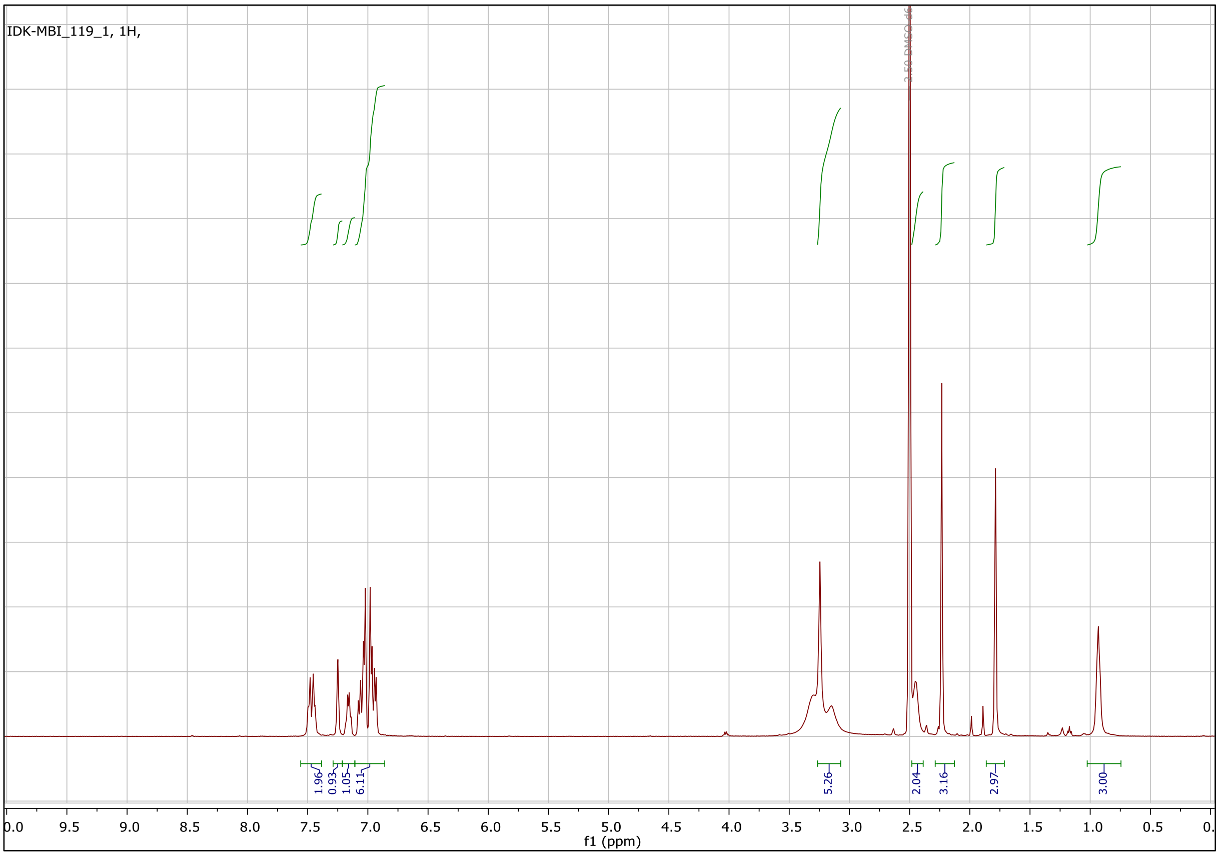

^{1}H NMR (300 MHz, DMSO-d_6) δ 7.56 – 7.38 (m, 2H), 7.29 – 7.21 (m, 1H), 7.16 (q, J = 7.3 Hz, 1H), 7.11 – 6.86 (m, 6H), 3.25 – 3.05 (m, 5H), 2.48 – 2.39 (m, 2H), 2.23 (s, 3H), 1.80 (s, 3H), 0.93 (t, J = 7.0 Hz, 3H).

IDK12037

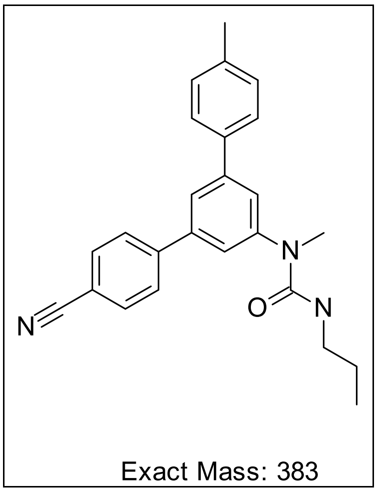

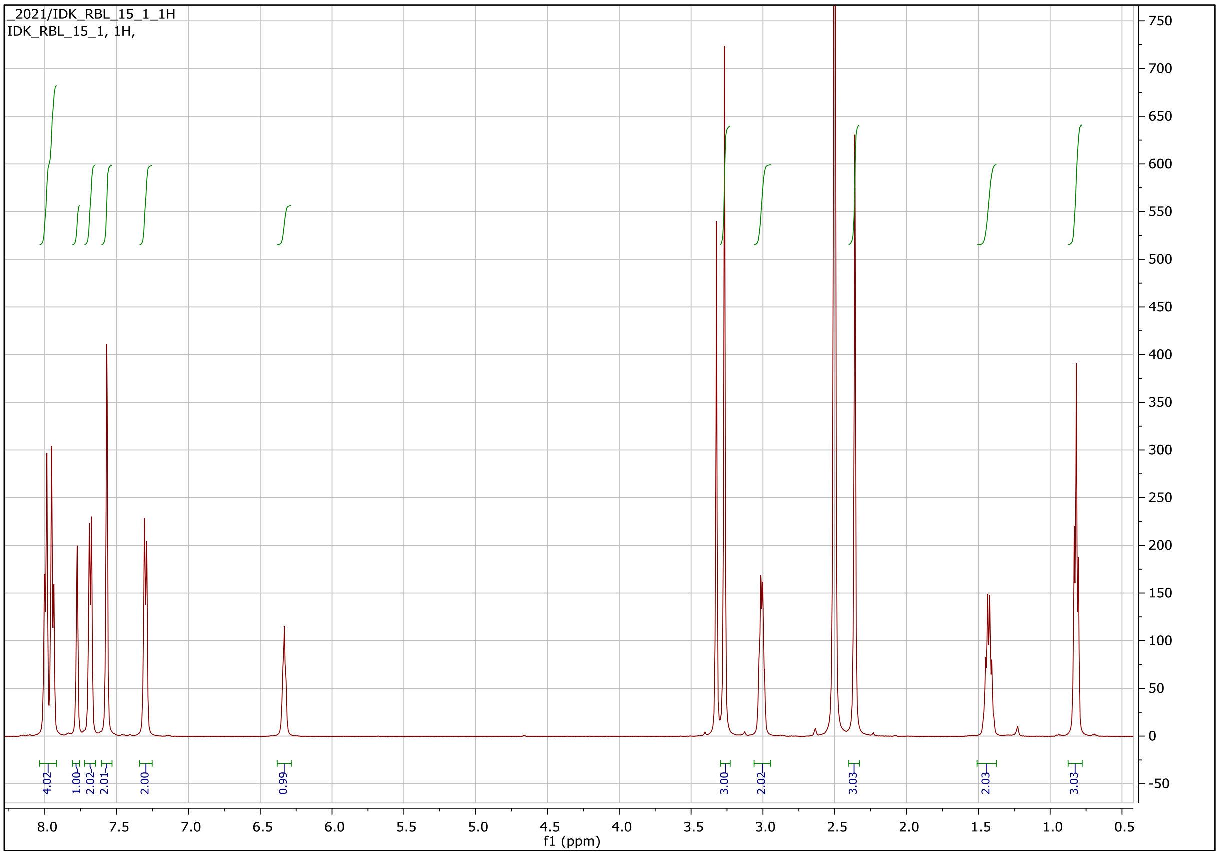

^{1}H NMR (500 MHz, DMSO-d_6) δ 8.04 – 7.92 (m, 4H), 7.77 (s, 1H), 7.68 (d, *J* = 7.7 Hz, 2H), 7.60 – 7.53 (m, 2H), 7.30 (d, *J* = 7.8 Hz, 2H), 6.33 (t, *J* = 5.5 Hz, 1H), 3.27 (s, 3H), 3.06 – 2.94 (m, 2H), 2.36 (s, 3H), 1.51 – 1.37 (m, 2H), 0.82 (t, *J* = 7.4 Hz, 3H).

IDK12038

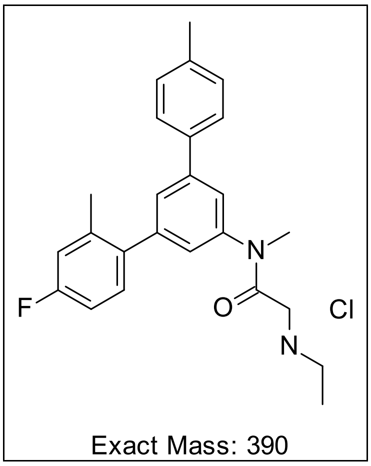

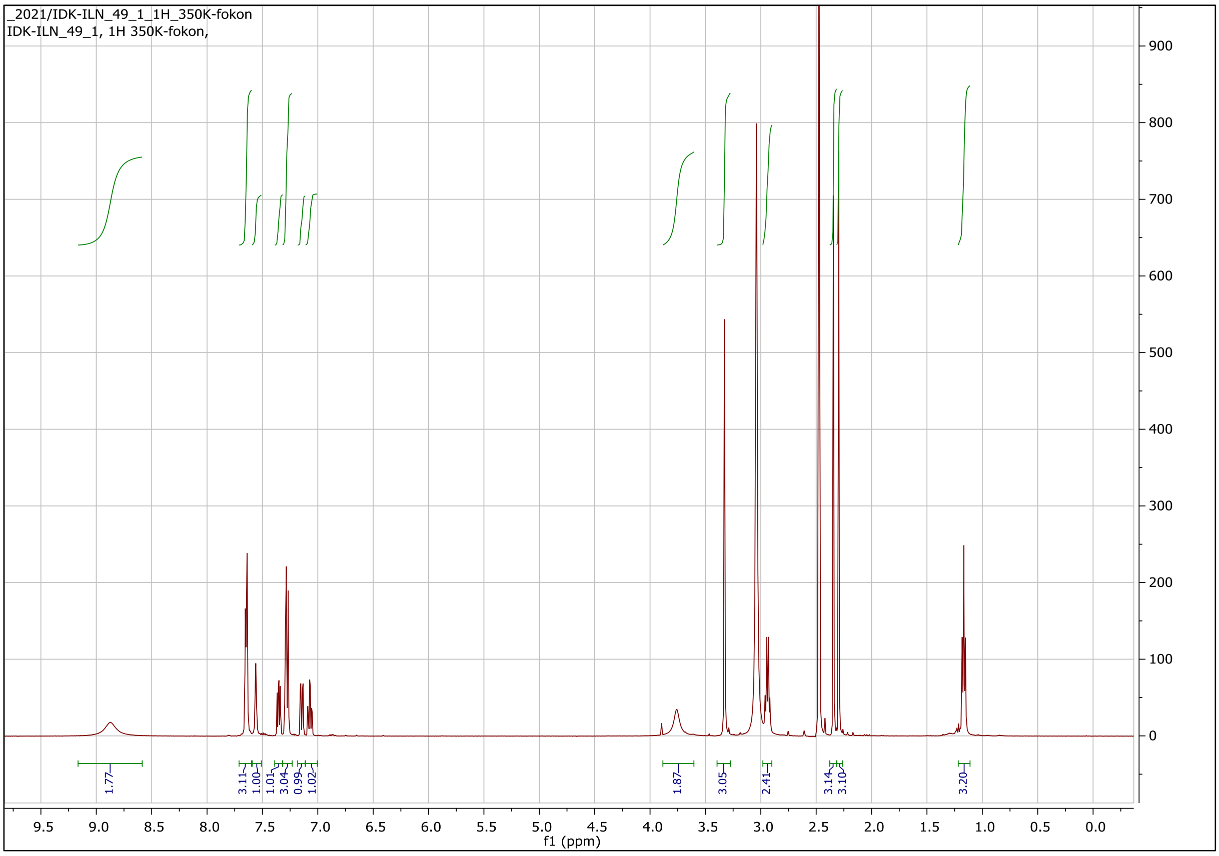

^{1}H NMR (500 MHz, DMSO-d_6) δ 8.87 (br s, 2H), 7.71 – 7.60 (m, 3H), 7.56 (s, 1H), 7.35 (dd, *J* = 8.5, 6.1 Hz, 1H), 7.32 – 7.23 (m, 3H), 7.15 (dd, *J* = 10.1, 2.7 Hz, 1H), 7.07 (td, *J* = 8.6, 2.8 Hz, 1H), 3.76 (br s, 2H), 3.33 (s, 3H), 2.94 (q, *J* = 7.2 Hz, 2H), 2.34 (s, 3H), 2.30 (s, 3H), 1.17 (t, *J* = 7.3 Hz, 3H). Note: at 350 K

IDK12042

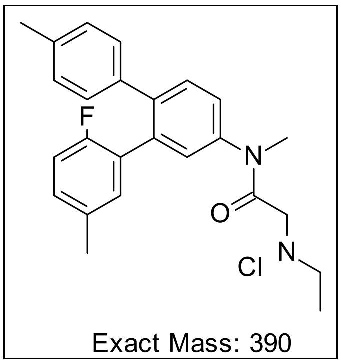

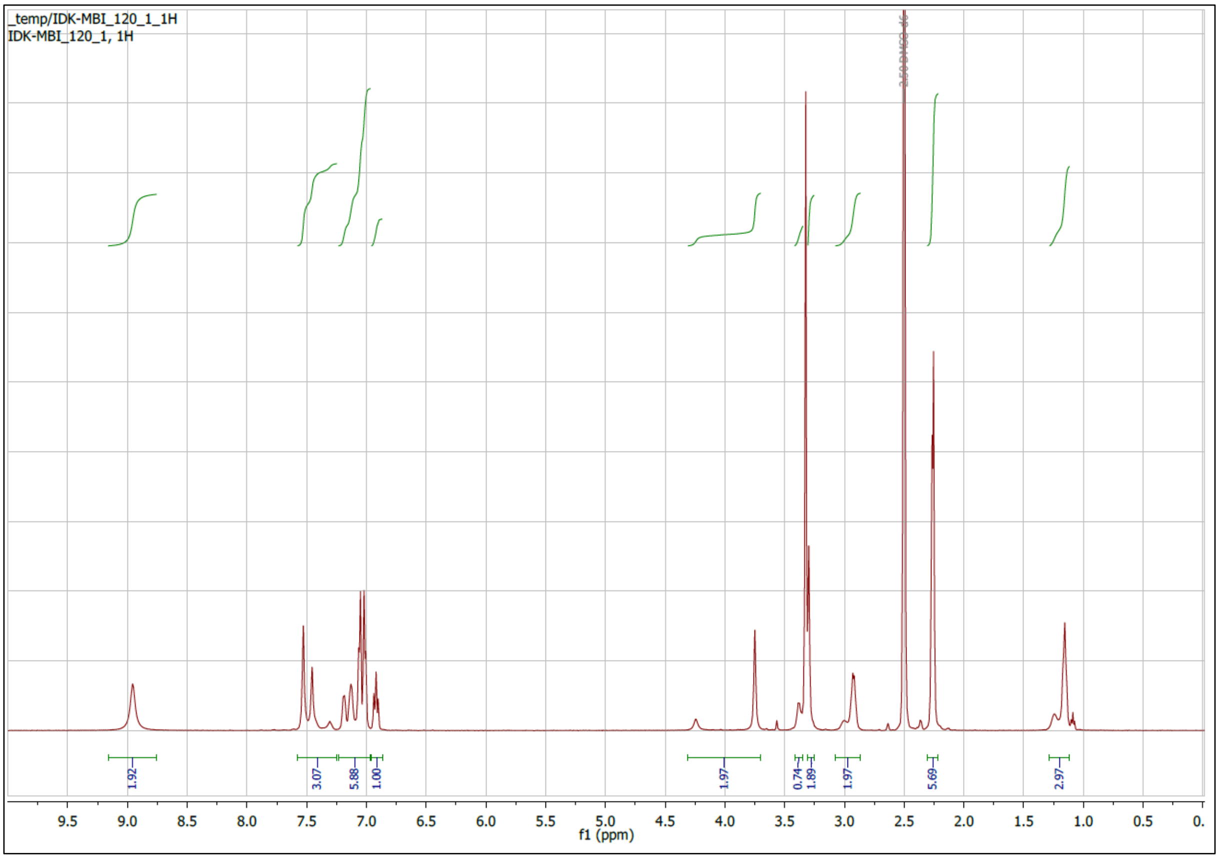

^{1}H NMR (300 MHz, DMSO-d_6) δ 8.96 (br.s, 2H), 7.58 – 7.25 (m, 3H), 7.23 – 6.97 (m, 6H), 6.92 (t, J = 9.2 Hz, 1H), 3.75 (s, 2H), 3.45 – 3.25 (m, 3H), 3.07 – 2.87 (m, 2H), 2.31 – 2.21 (m, 6H), 1.29 – 1.11 (m, 3H). Rotamers observed.

IDK12044

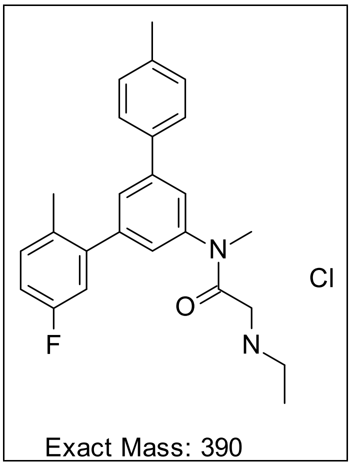

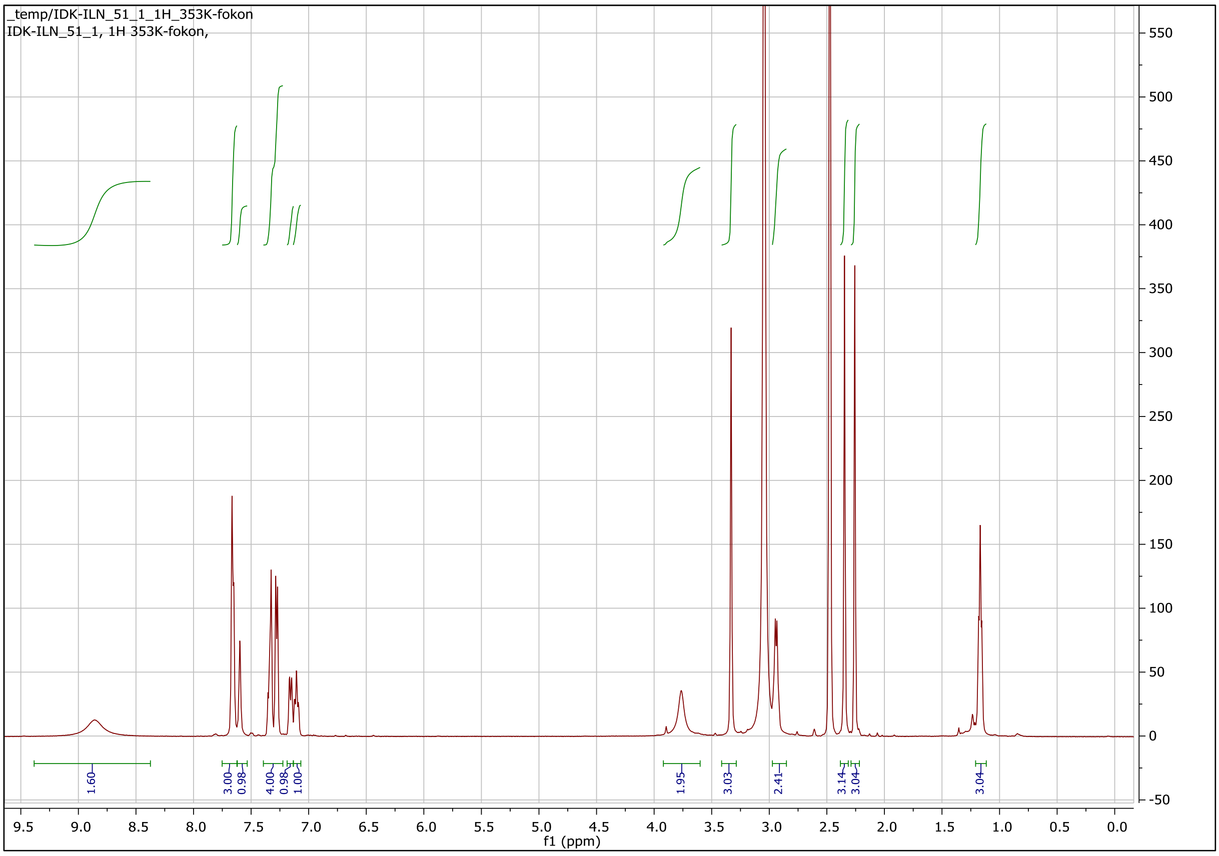

^{1}H NMR (500 MHz, DMSO-d_6) δ 8.85 (s, 2H), 7.75 – 7.62 (m, 3H), 7.60 (s, 1H), 7.39 – 7.22 (m, 4H), 7.16 (d, *J* = 9.8 Hz, 1H), 7.10 (td, *J* = 8.5, 3.0 Hz, 1H), 3.76 (s, 2H), 3.33 (s, 3H), 2.94 (q, *J* = 7.2 Hz, 2H), 2.35 (s, 3H), 2.26 (s, 3H), 1.17 (t, J = 7.2 Hz, 3H). Note: at 353 K, the sample contains some grease

IDK12049

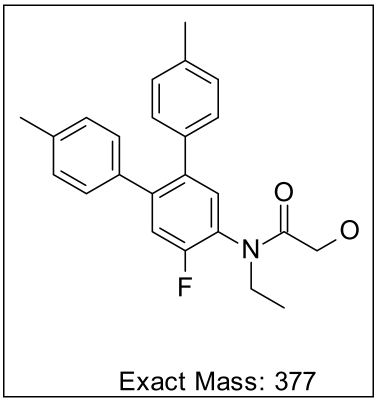

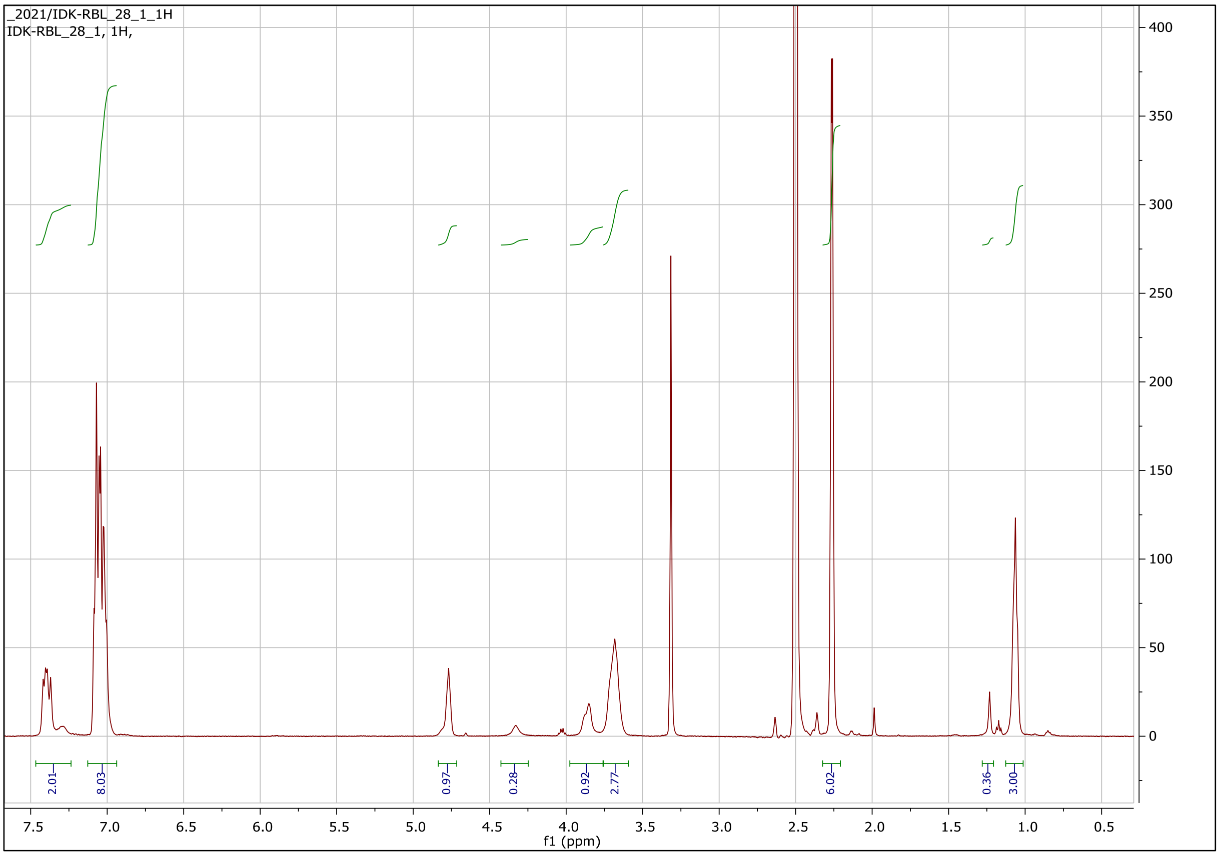

^{1}H NMR (500 MHz, DMSO-d_6) δ 7.47 – 7.24 (m, 2H), 7.13 – 6.94 (m, 8H), 4.84 – 4.72 (m, 1H), 3.93 – 3.80 (m, 1H), 3.76 – 3.59 (m, 3H), 2.26 (d, J = 4.4 Hz, 6H), 1.06 (t, J = 6.9 Hz, 3H).

Note: contains 4 mol% EtOAc

IDK12056

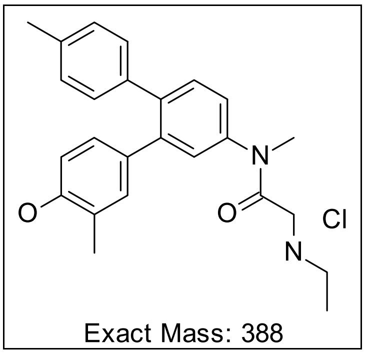

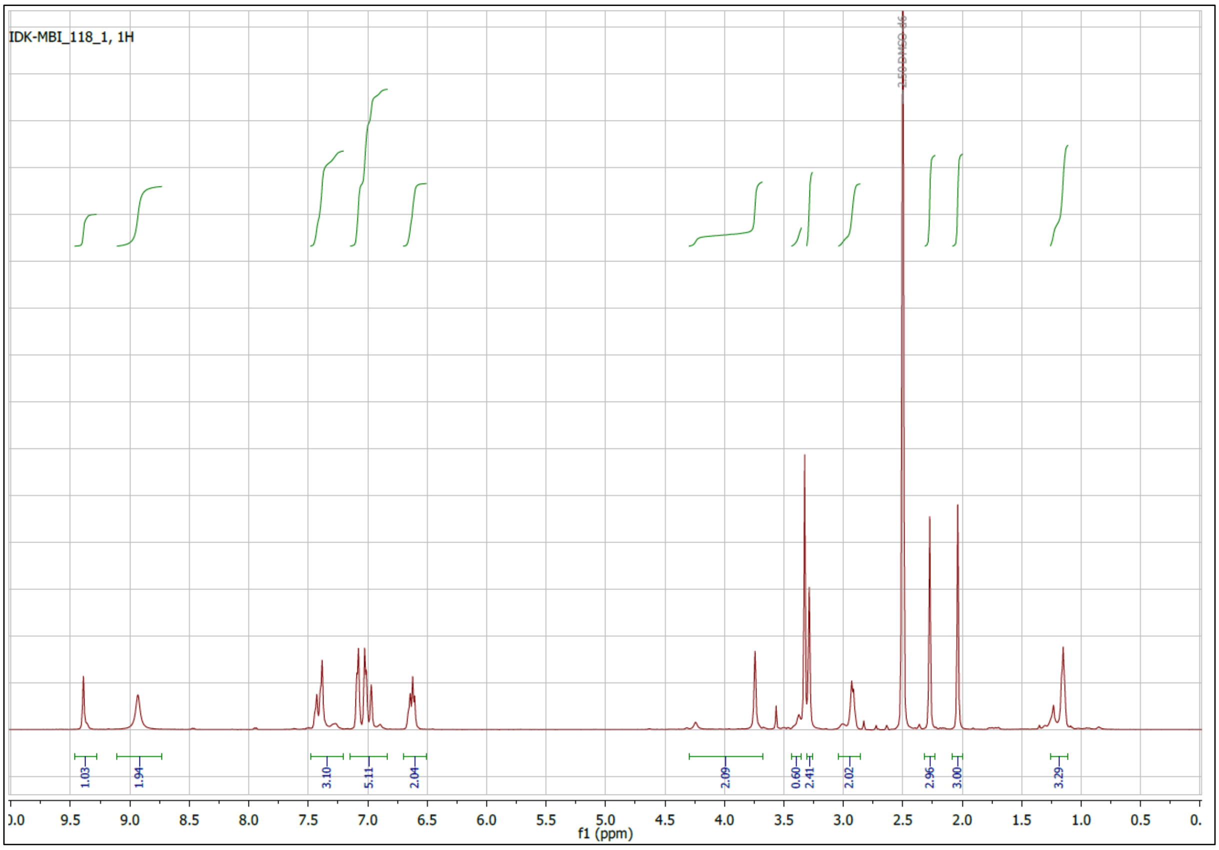

^{1}H NMR (300 MHz, DMSO-d_6) δ 9.39 (s, 1H), 8.93 (br.s, 2H), 7.48 – 7.20 (m, 3H), 7.15 – 6.83 (m, 5H), 6.71 – 6.50 (m, 2H), 3.74 (s, 2H), 3.43 – 3.25 (m, 3H), 3.05 – 2.85 (m, 2H), 2.27 (s, 3H), 2.04 (s, 3H), 1.26 – 1.11 (m, 3H). Rotamers observed.

IDK12058

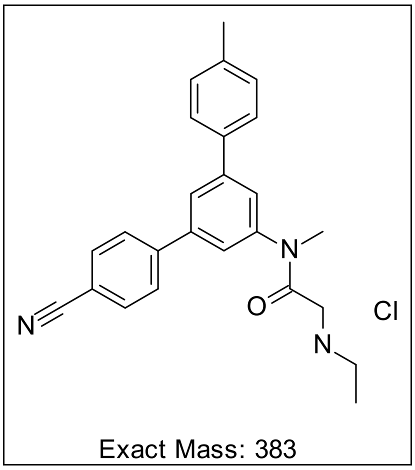

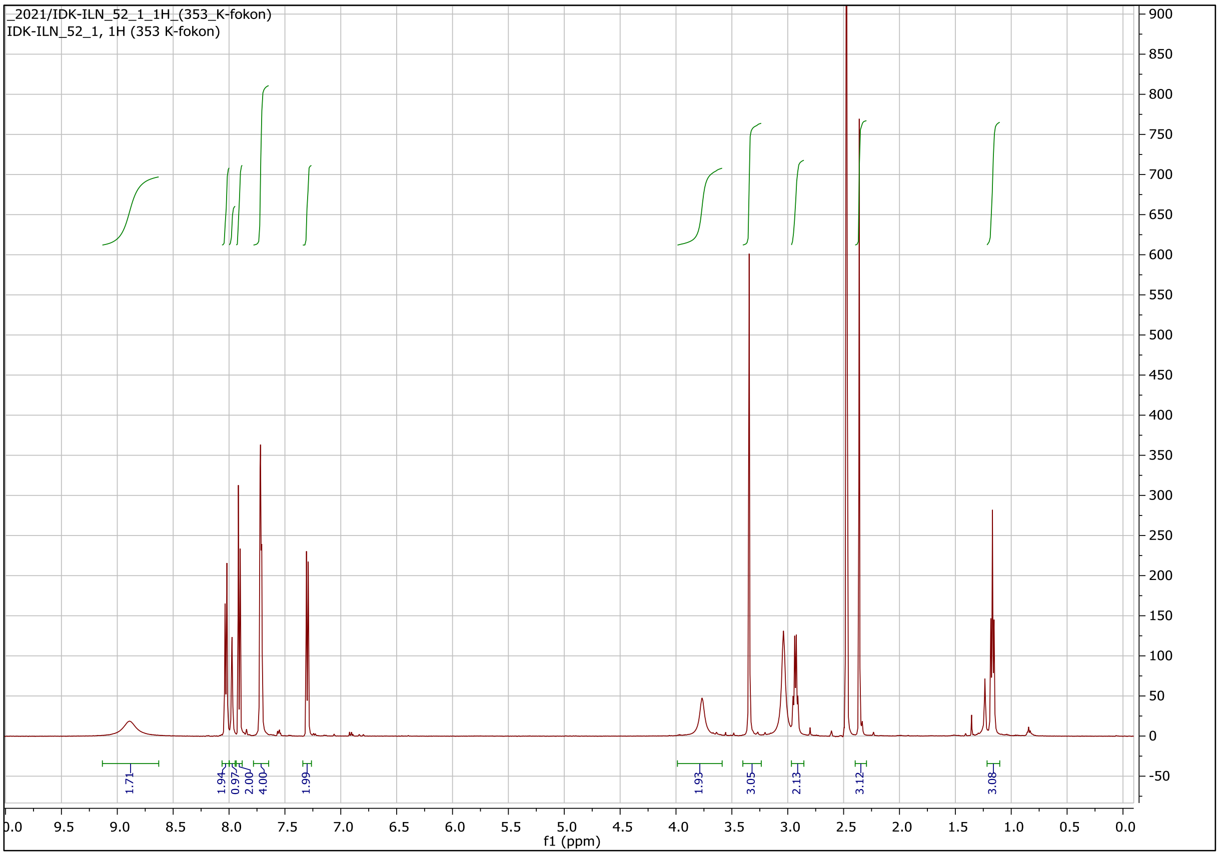

^{1}H NMR (500 MHz, DMSO-d_6) δ 8.89 (br s, 2H), 8.03 (d, *J* = 8.1 Hz, 2H), 7.97 (s, 1H), 7.94 – 7.88 (m, 2H), 7.78 – 7.65 (m, 4H), 7.30 (d, *J* = 7.9 Hz, 2H), 3.77 (br s, 2H), 3.35 (s, 3H), 2.93 (q, *J* = 7.3 Hz, 2H), 2.36 (s, 3H), 1.17 (t, *J* = 7.3 Hz, 3H) at 353K.
Note: the sample contains grease

IDK12065

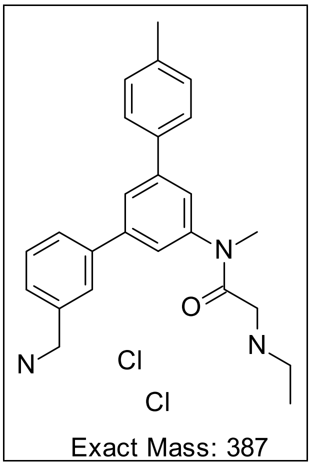

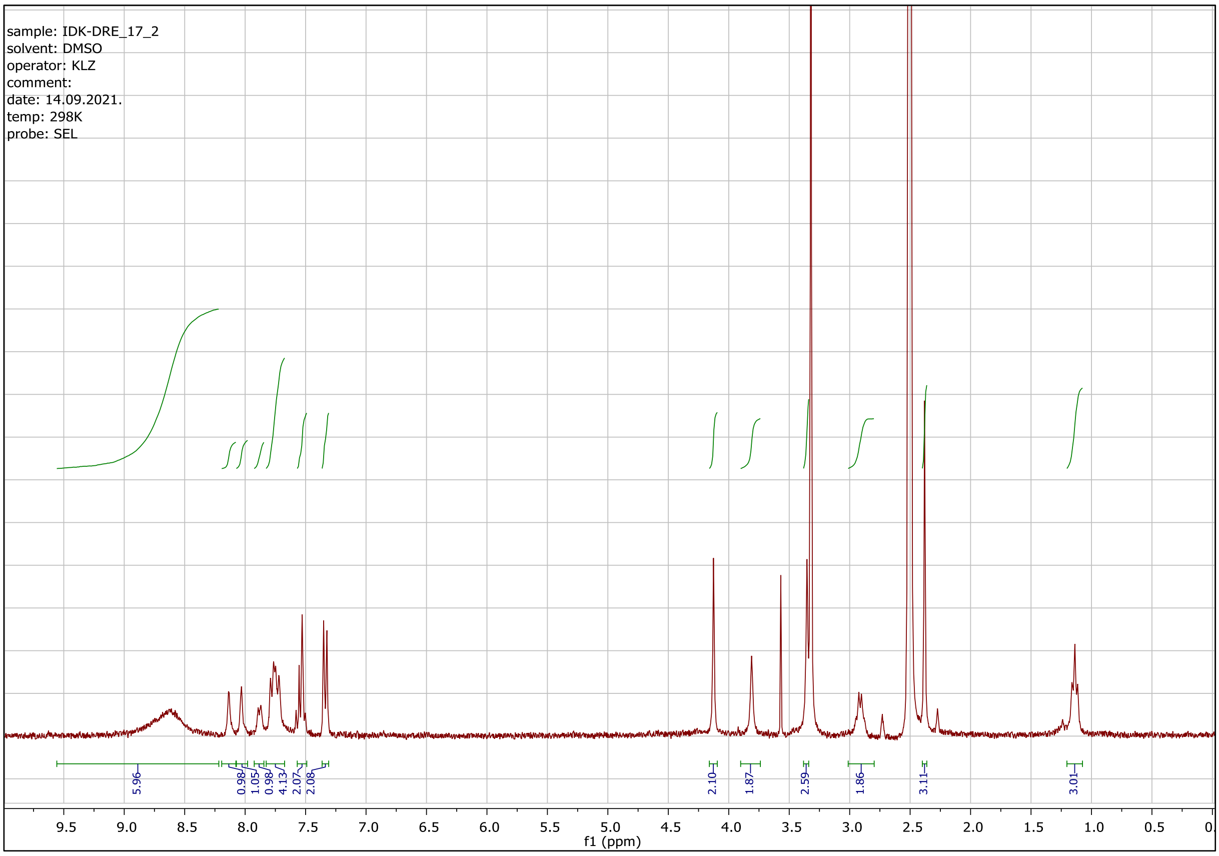

^{1}H NMR (300 MHz, DMSO-d_6) δ 8.61 (br.s, 6H), 8.14 (s, 1H), 8.03 (s, 1H), 7.88 (d, *J* = 6.9 Hz, 1H), 7.76 (dd, *J* = 13.2, 7.9 Hz, 4H), 7.57 – 7.49 (m, 2H), 7.34 (d, *J* = 8.0 Hz, 2H), 4.13 (s, 2H), 3.81 (s, 2H), 3.35 (s, 3H), 3.01 - 2.80 (m, 2H), 2.38 (s, 3H), 1.14 (t, *J* = 7.2 Hz, 3H).

Cont. 2 w/w% 1,4-dioxane.

IDK12068

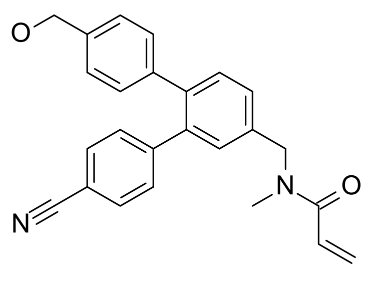

Exact Mass: 382

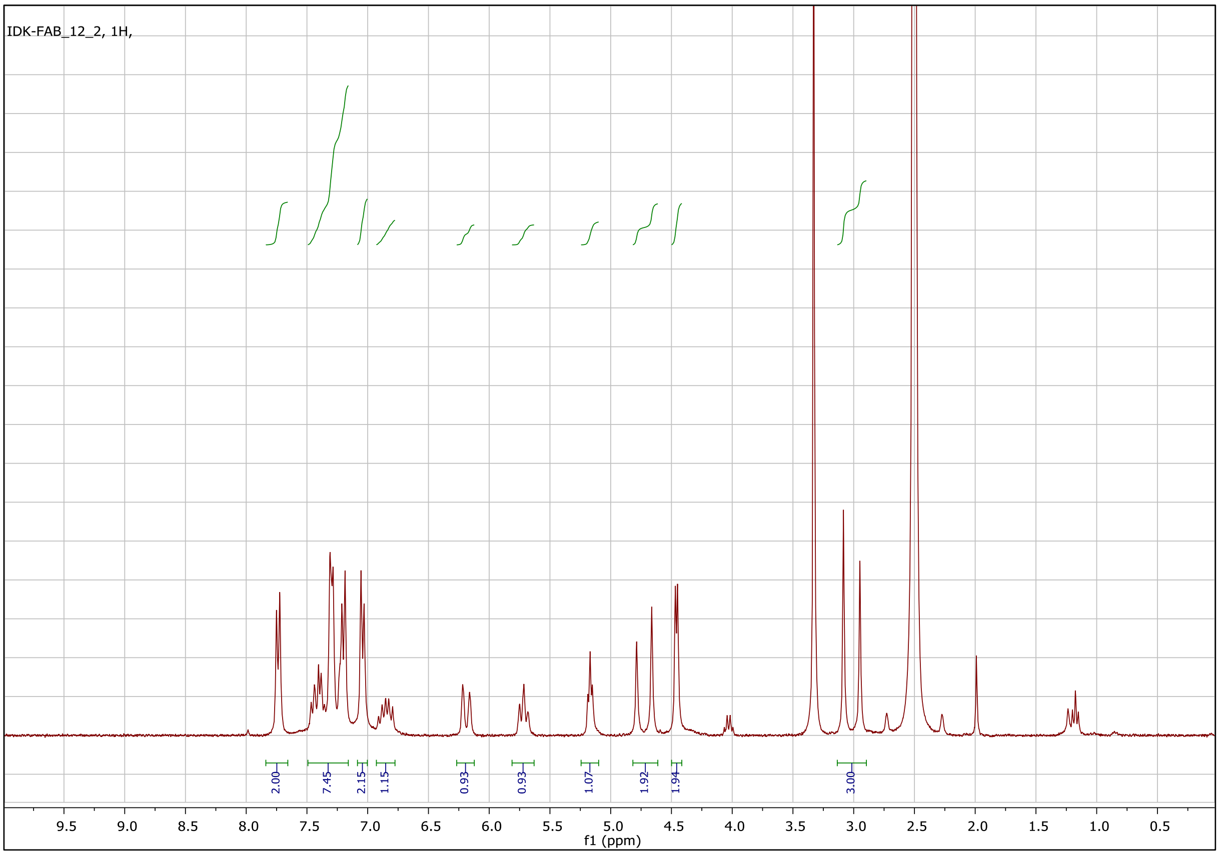

^{1}H NMR (300 MHz, DMSO-d_6) δ 7.74 (d, *J* = 7.9 Hz, 2H), 7.49 – 7.16 (m, 7H), 7.04 (d, *J* = 7.8 Hz, 2H), 6.94 – 6.77 (m, 1H), 6.27 – 6.12 (m, 1H), 5.71 (t, *J* = 10.2 Hz, 1H), 5.17 (t, *J* = 5.7 Hz, 1H), 4.81 – 4.59 (m, 2H), 4.46 (d, *J* = 5.6 Hz, 2H), 3.14 – 2.90 (m, 3H). contains 3.3 w/w% EtOAc

IDK12069

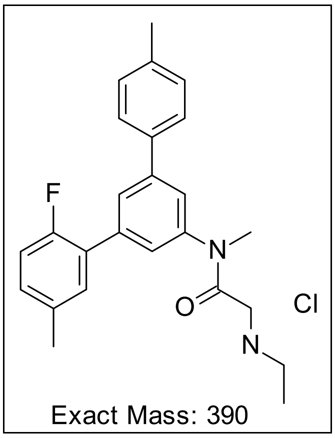

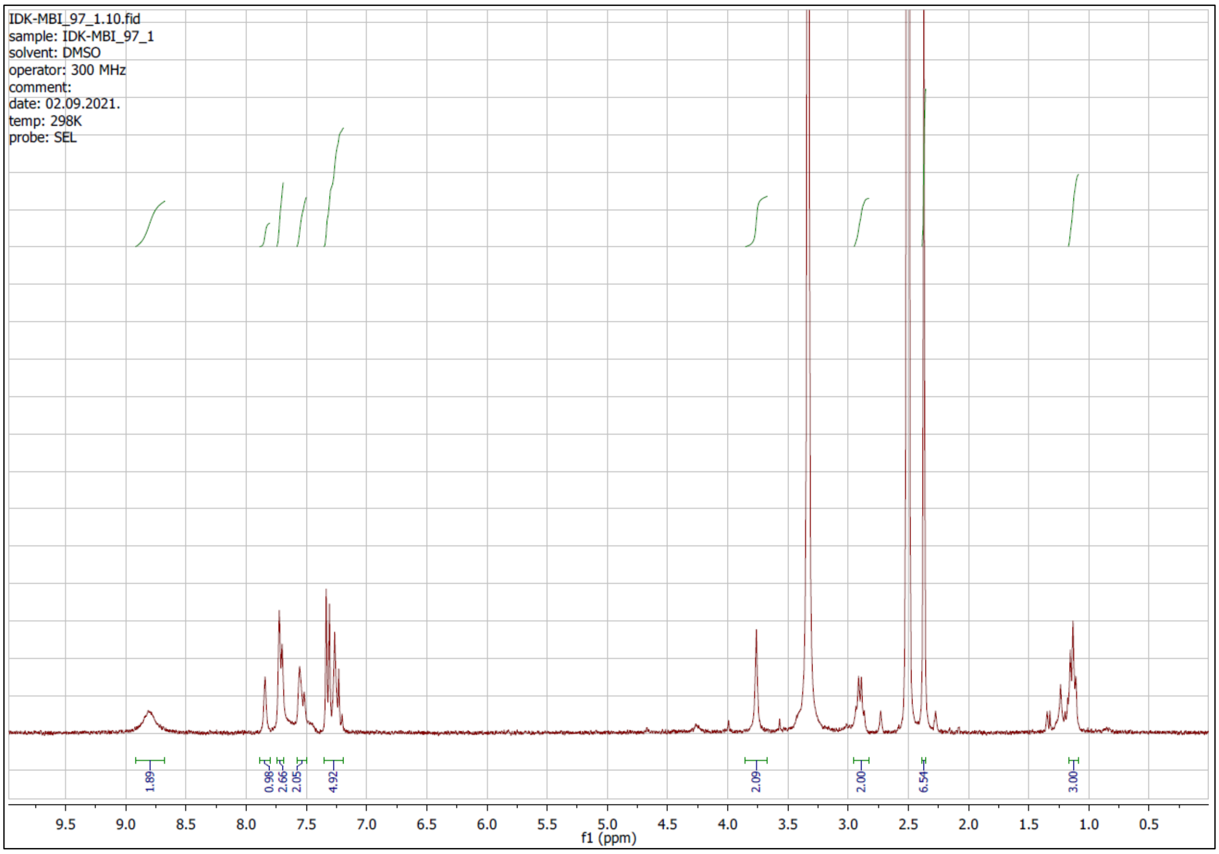

^{1}H NMR (300 MHz, DMSO-d_6) δ 8.81 (br.s, 2H), 7.84 (s, 1H), 7.76 – 7.69 (m, 2H), 7.60 – 7.49 (m, 2H), 7.36 – 7.19 (m, 5H), 3.76 (s, 2H), 2.90 (q, *J* = 7.1 Hz, 2H), 2.37 (s, 6H), 1.13 (t, *J* = 7.1 Hz, 3H). Note: N-metil signal is under water signal

IDK12070

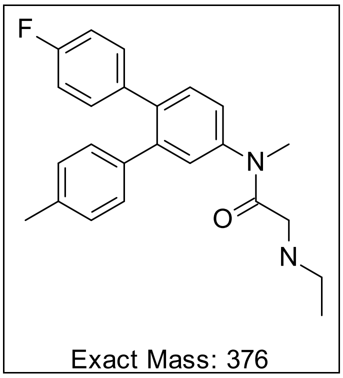

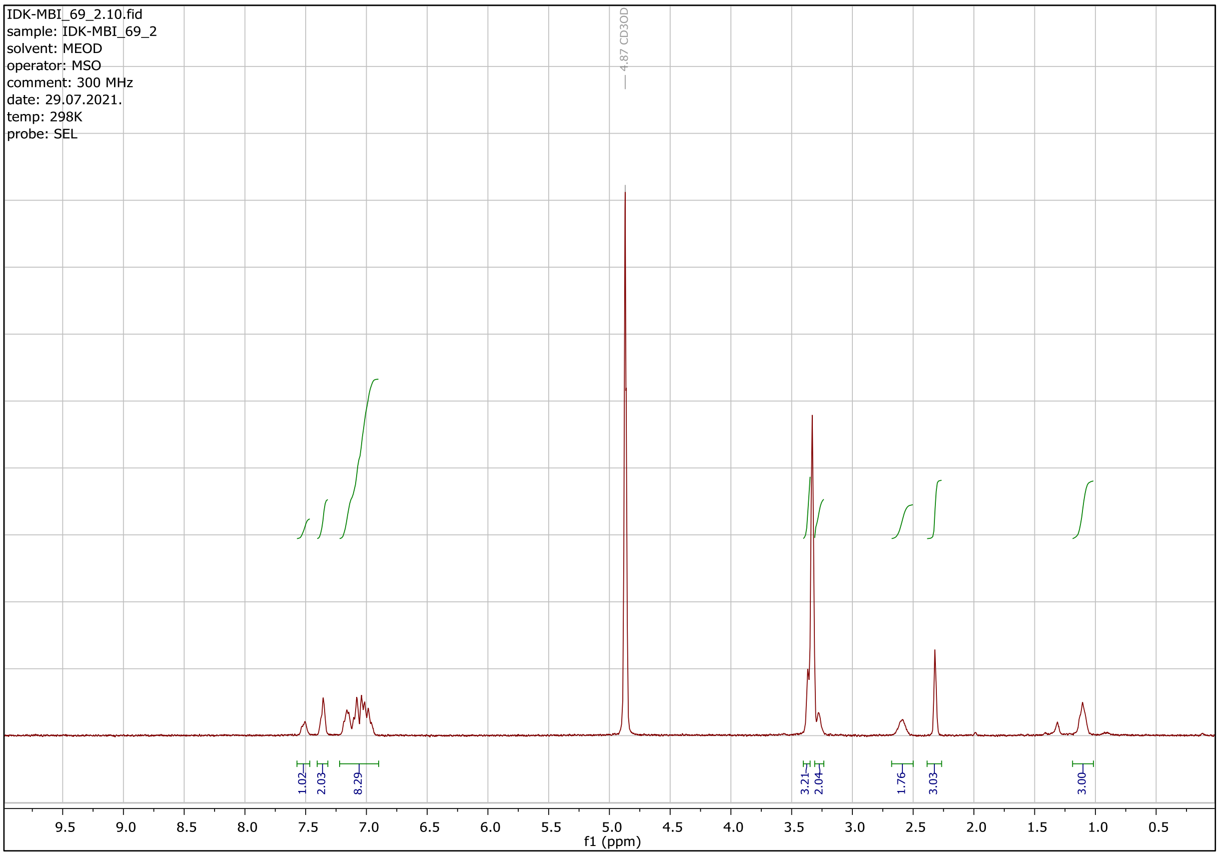

^{1}H (300 MHz, Methanol-d_4) δ 7.57 – 7.47 (m, 1H), 7.40 – 3.32 (m, 2H), 7.22 – 6.90 (m, 8H), 3.37 (s, 3H), 3.27 (br.s, 2H), 2.67 – 2.50 (m, 2H), 2.32 (s, 3H), 1.12 (t, *J* = 7.8 Hz, 3H).

IDK12074

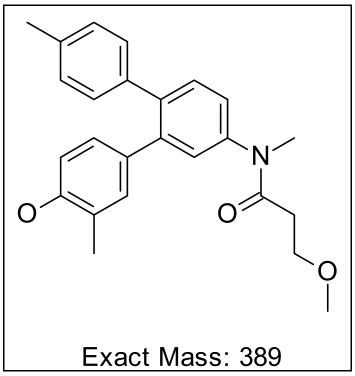

melting range: 174-177 °C

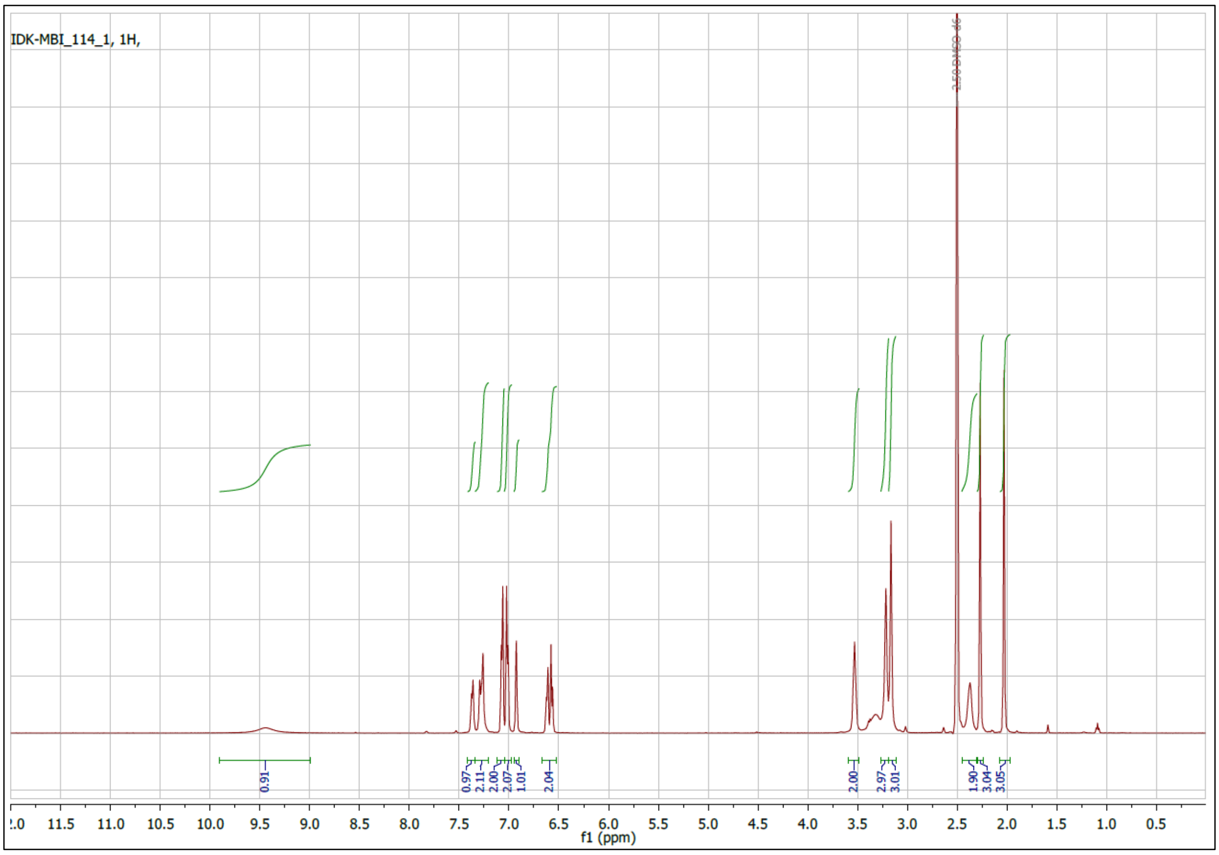

^{1}H NMR (300 MHz, DMSO-d_6) δ 9.45 (br.s, 1H), 7.37 (d, *J* = 8.0 Hz, 1H), 7.34 – 7.21 (m, 2H), 7.07 (d, *J* = 7.7 Hz, 2H), 7.02 (d, *J* = 7.8 Hz, 2H), 6.93 (s, 1H), 6.67 – 6.52 (m, 2H), 3.53 (t, *J* = 6.2 Hz, 2H), 3.22 (s, 3H), 3.16 (s, 3H), 2.37 (br.s, 2H), 2.27 (s, 3H), 2.03 (s, 3H).

IDK12078

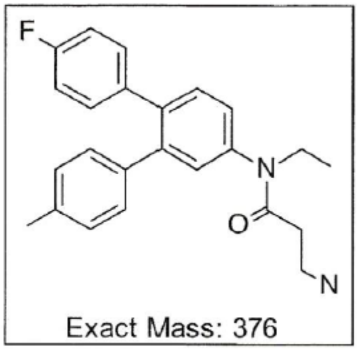

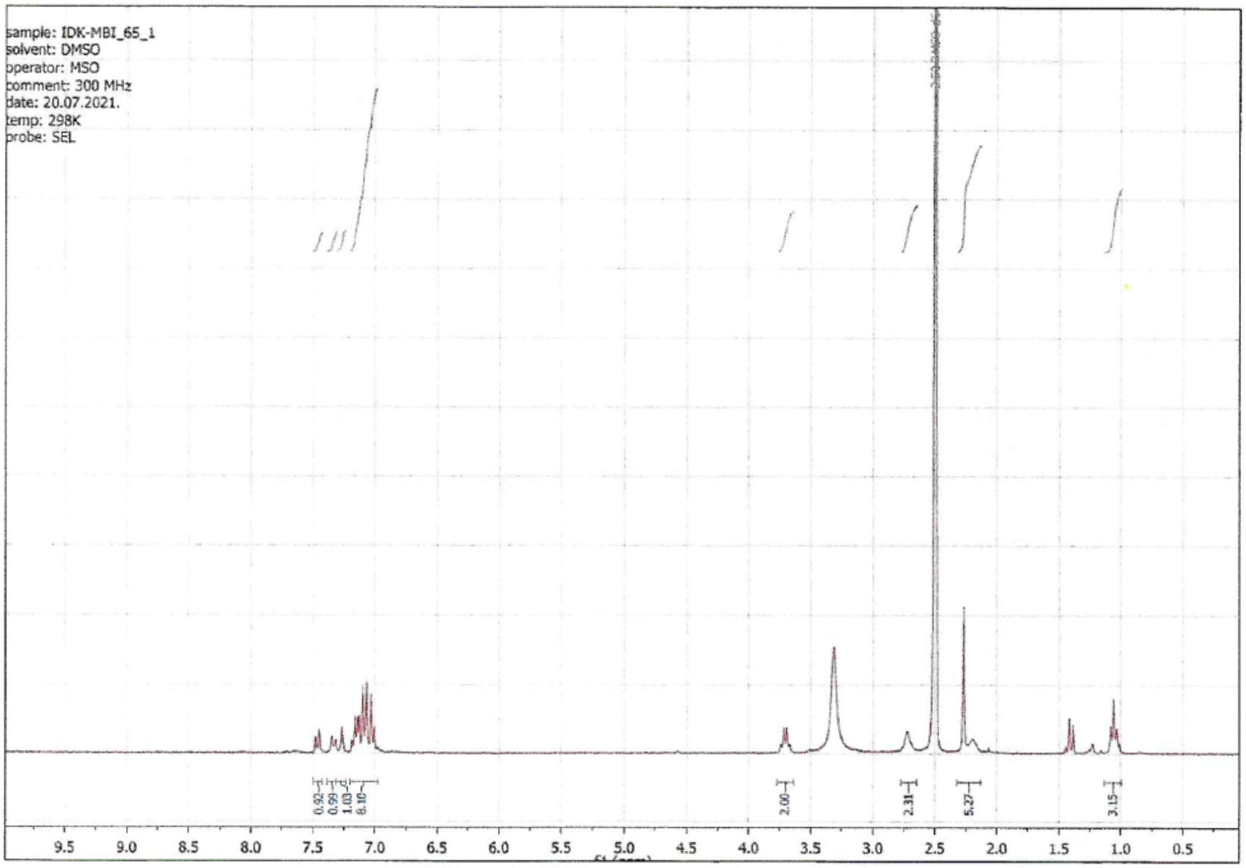

^{1}H NMR (300 MHz, DMSO-d_6) δ 7.46 (d, J = 7.9 Hz, 1H), 7.33 (dd, J=7.9, 2.2 Hz, 1H), 7.26 (d, J=2.2 Hz, 1H), 7.21 - 6.98 (m, 8H), 3.70 (q, J=7.0 Hz, 2H), 2.32 - 2.14 (m, 5H), 1.05 (t, J=7.0 Hz, 3H)

IDK12082

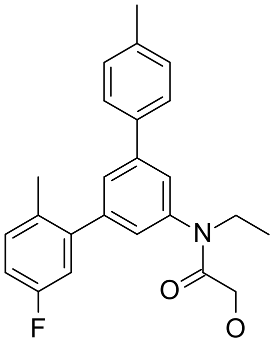

Exact Mass: 377

Melting Range: slow melting above 160°C

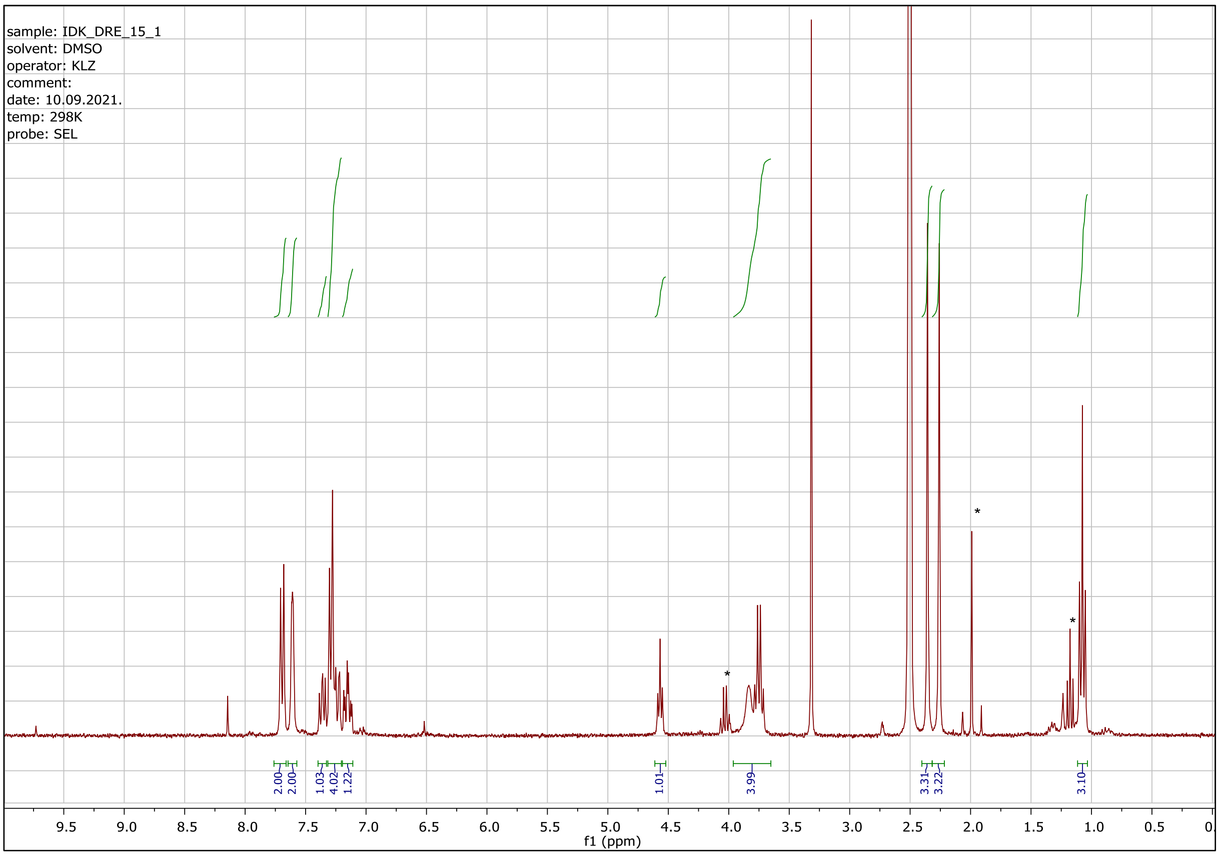

^{1}H NMR (300 MHz, DMSO-d_6) δ 7.69 (d, J = 8.1 Hz, 2H), 7.61 (p, J = 1.8 Hz, 2H), 7.36 (dd, J = 8.5, 6.0 Hz, 1H), 7.32 – 7.20 (m, 4H), 7.15 (td, J = 8.5, 2.8 Hz, 1H), 4.57 (t, J = 5.7 Hz, 1H), 3.96 – 3.65 (m, 4H), 2.36 (s, 3H), 2.26 (s, 3H), 1.08 (t, J = 7.1 Hz, 3H). Cont. 4.5 w/w% EtAOc.

IDK12084

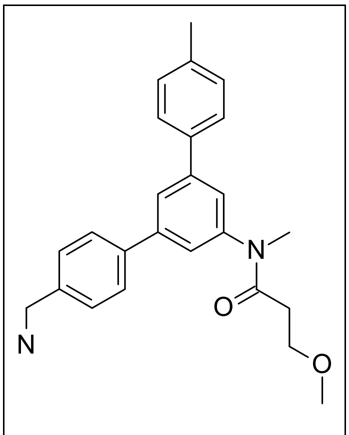

Exact Mass: 388

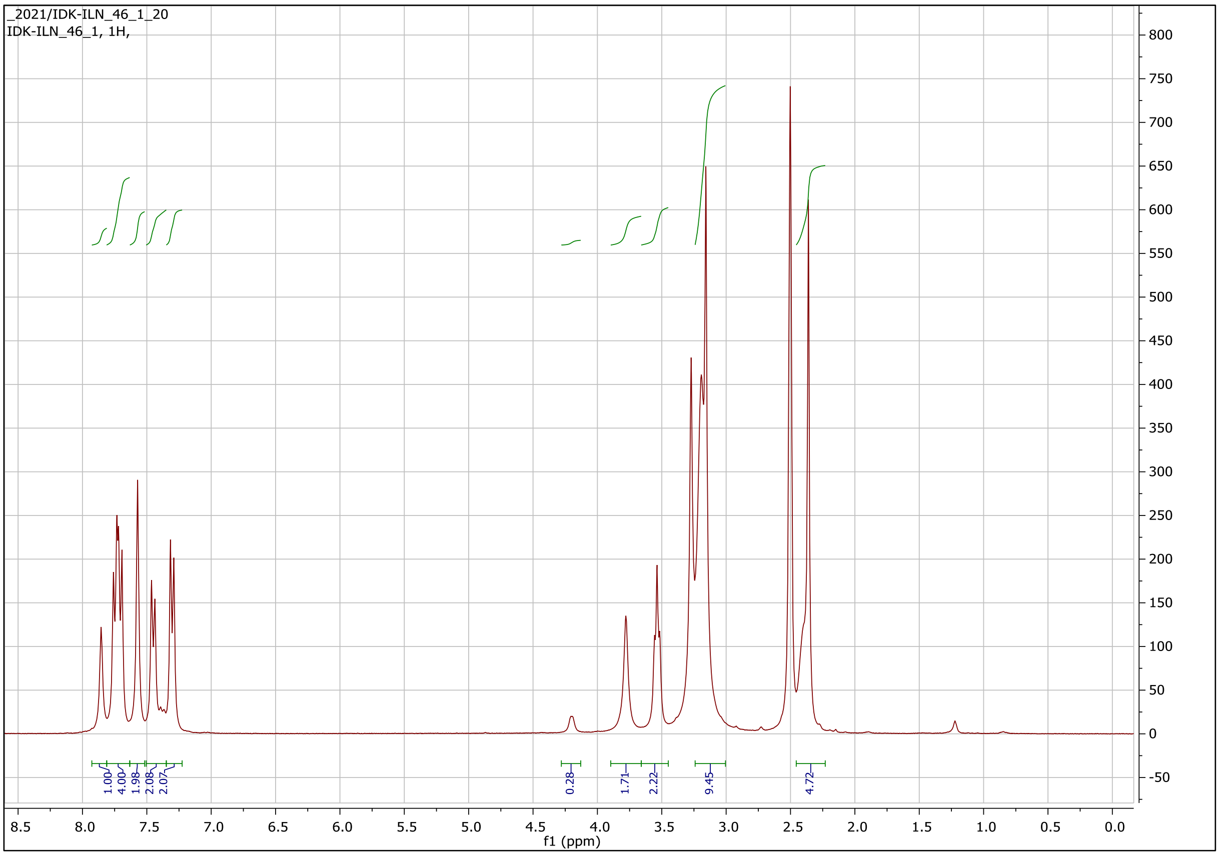

^{1}H NMR (300 MHz, DMSO-d_6) δ 7.85 (s, 1H), 7.81 – 7.63 (m, 4H), 7.57 (s, 2H), 7.45 (d, *J* = 7.9 Hz, 2H), 7.30 (d, *J* = 7.8 Hz, 2H), 3.78 (s, 2H), 3.54 (t, *J* = 6.4 Hz, 2H), 3.24 – 3.00 (m, 9H), 2.46 – 2.23 (m, 5H).

IDK12092

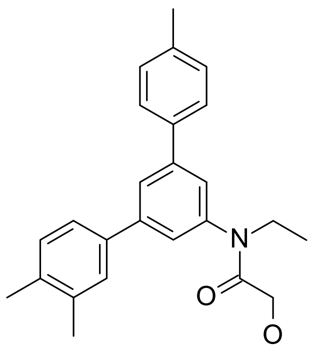

Exact Mass: 373

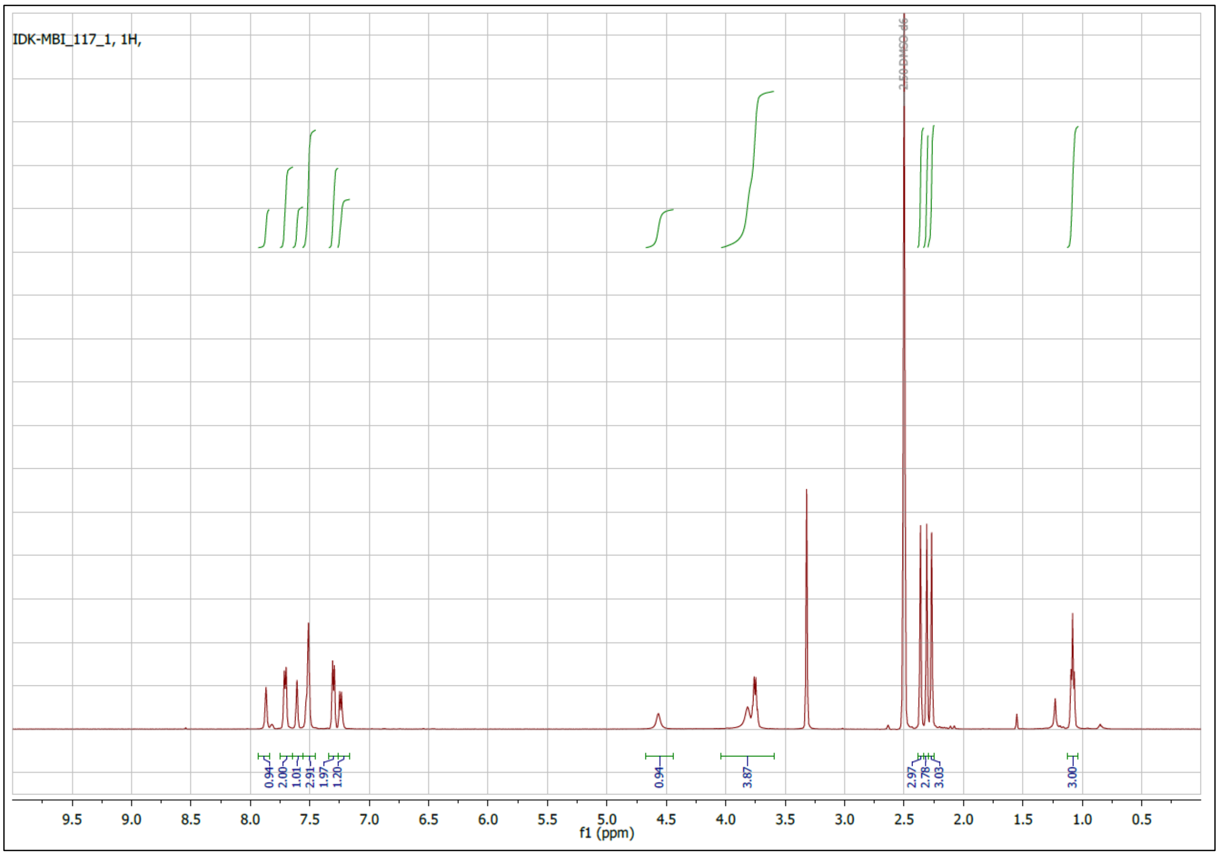

^{1}H NMR (300 MHz, DMSO-d_6) δ 7.87 (s, 1H), 7.71 (d, J = 7.6 Hz, 2H), 7.61 (s, 1H), 7.52 (d, J = 9.6 Hz, 3H), 7.30 (d, J = 7.7 Hz, 2H), 7.24 (d, J = 8.1 Hz, 1H), 4.57 (br.s, 1H), 4.04 – 3.60 (m, 4H), 2.36 (s, 3H), 2.31 (s, 3H), 2.27 (s, 3H), 1.08 (t, J = 7.1 Hz, 3H).

IDK12095

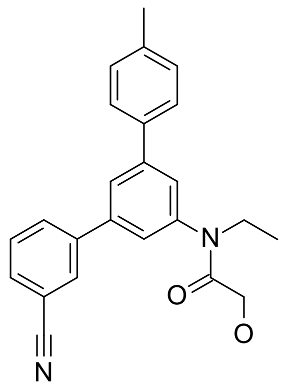

Exact Mass: 370

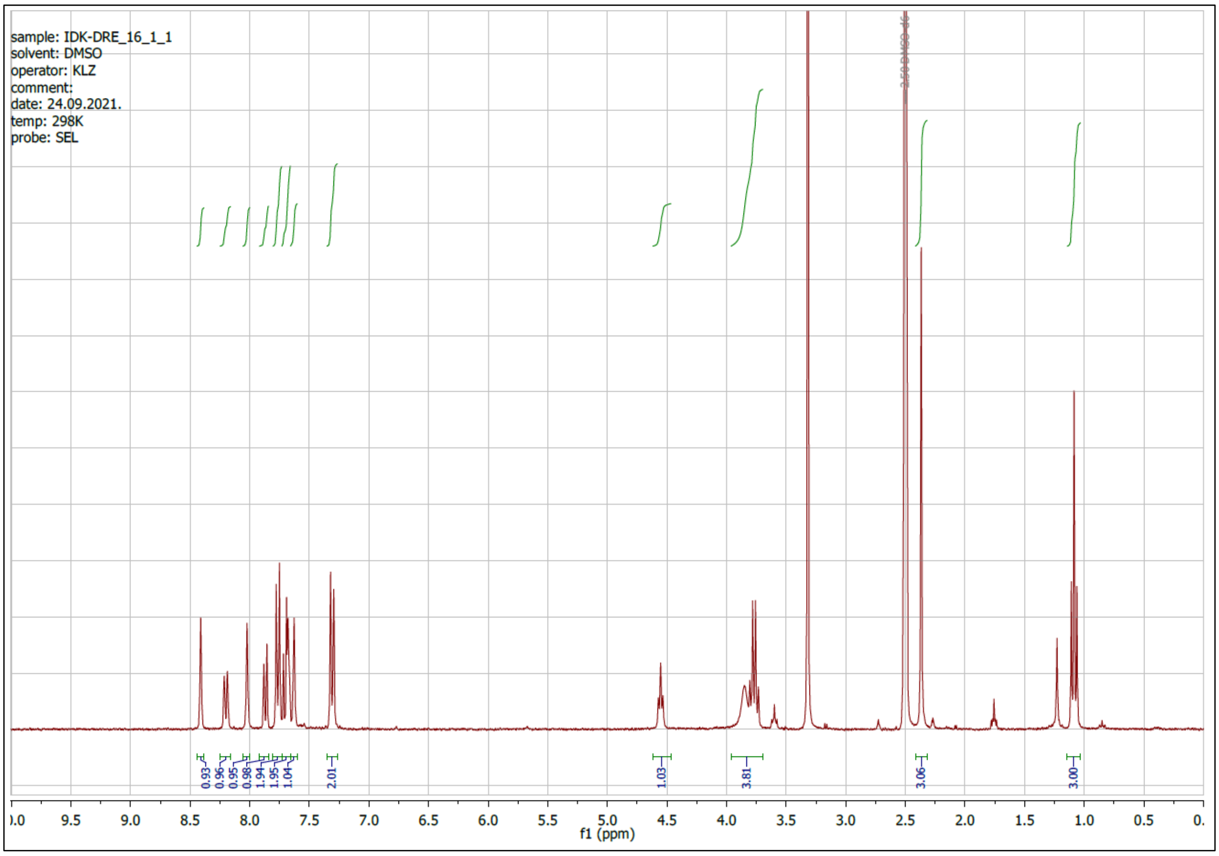

^{1}H NMR (300 MHz, DMSO-d_6) δ 8.41 (d, *J* = 1.7 Hz, 1H), 8.20 (dt, *J* = 8.0, 1.5 Hz, 1H), 8.02 (d, *J* = 1.7 Hz, 1H), 7.87 (dt, *J* = 7.7, 1.3 Hz, 1H), 7.81 – 7.73 (m, 2H), 7.73 – 7.66 (m, 2H), 7.63 (d, *J* = 1.8 Hz, 1H), 7.31 (d, *J* = 8.0 Hz, 2H), 4.55 (t, *J* = 5.7 Hz, 1H), 3.96 – 3.70 (m, 4H), 2.37 (s, 3H), 1.08 (t, *J* = 7.1 Hz, 3H). Contains THF traces

IDK12097

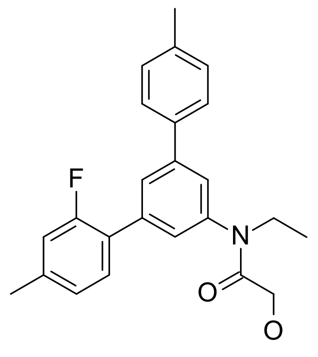

Exact Mass: 377

melting range: 118-122 °C

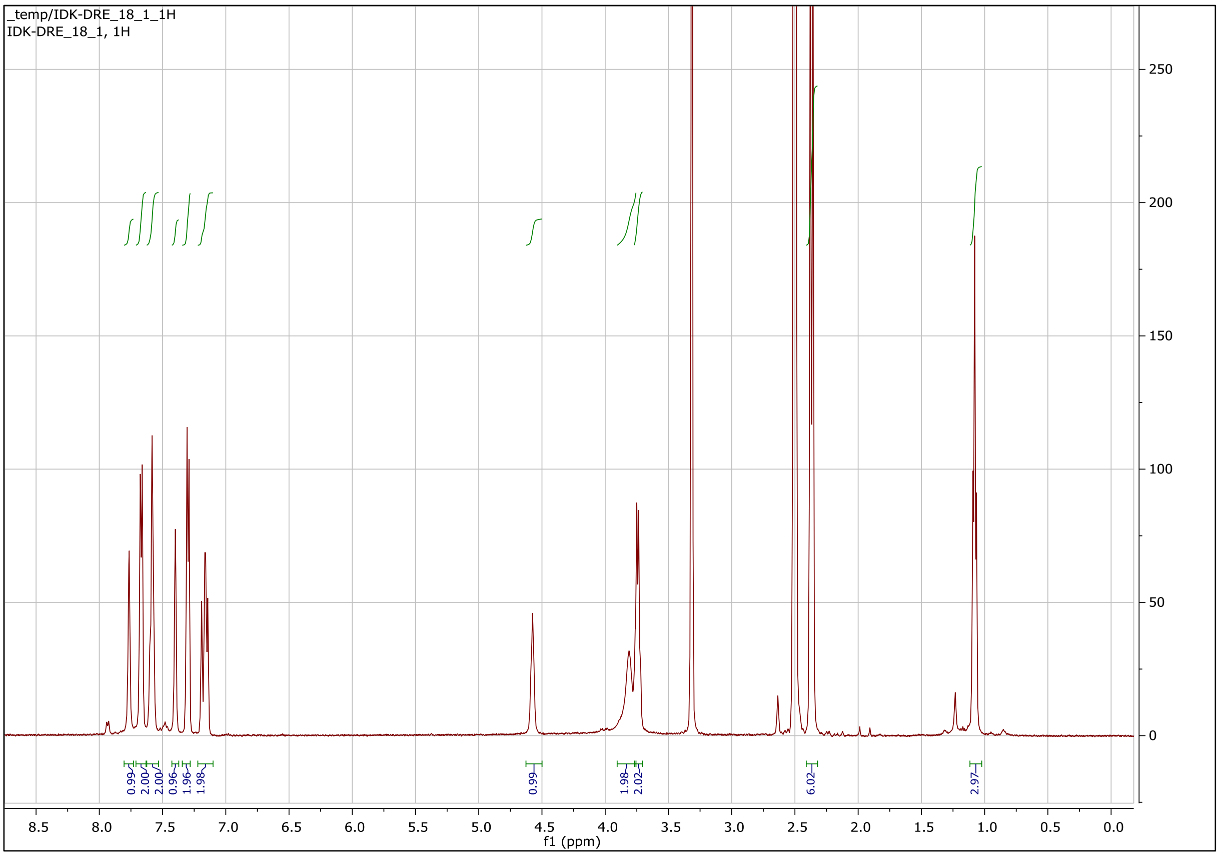

^{1}H NMR (500 MHz, DMSO-d_6) δ 7.76 (s, 1H), 7.67 (d, J = 7.6 Hz, 2H), 7.62 – 7.53 (m, 2H), 7.40 (s, 1H), 7.30 (d, J = 7.8 Hz, 2H), 7.22 – 7.10 (m, 2H), 4.57 (t, J = 5.6 Hz, 1H), 3.81 (br s, 2H), 3.74 (q, J = 7.0 Hz, 2H), 2.38 (s, 3H), 2.36 (s, 3H), 1.08 (t, J = 7.1 Hz, 3H).
Note: the sample contains some grease

IDK12098

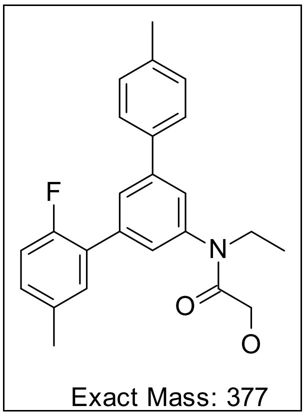

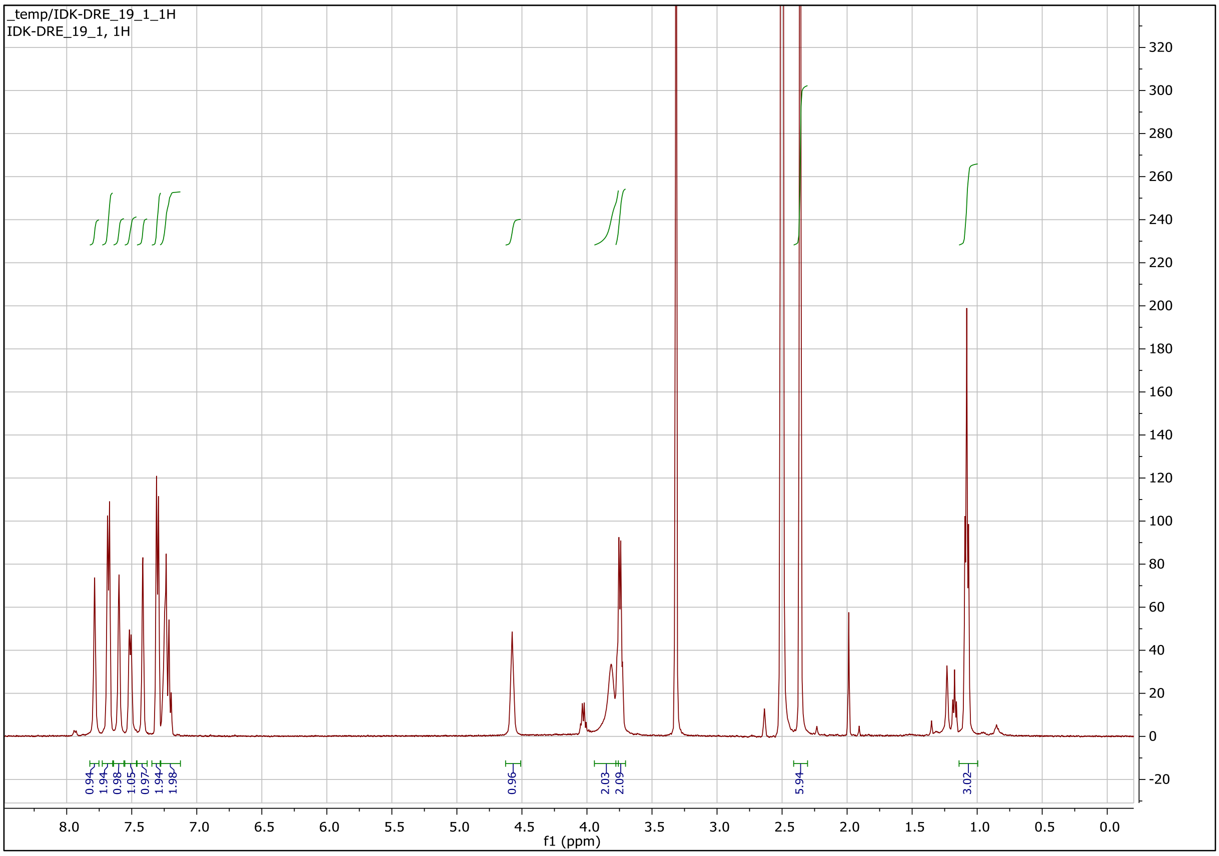

^{1}H NMR (500 MHz, DMSO-d_6) δ 7.79 (s, 1H), 7.68 (d, *J* = 7.7 Hz, 2H), 7.60 (s, 1H), 7.51 (d, *J* = 7.6 Hz, 1H), 7.41 (s, 1H), 7.30 (d, *J* = 7.8 Hz, 2H), 7.28 – 7.13 (m, 2H), 4.58 (t, *J* = 5.8 Hz, 1H), 3.79 (br s, 2H), 3.75 (q, *J* = 7.2 Hz, 2H), 2.36 (s, 6H), 1.08 (t, *J* = 7.1 Hz, 3H).
Note: the sample contains ~2.9 w/w% ethyl acetate and some grease

IDK12176

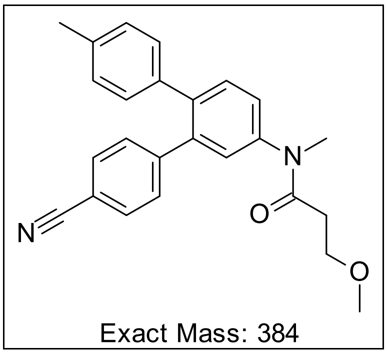

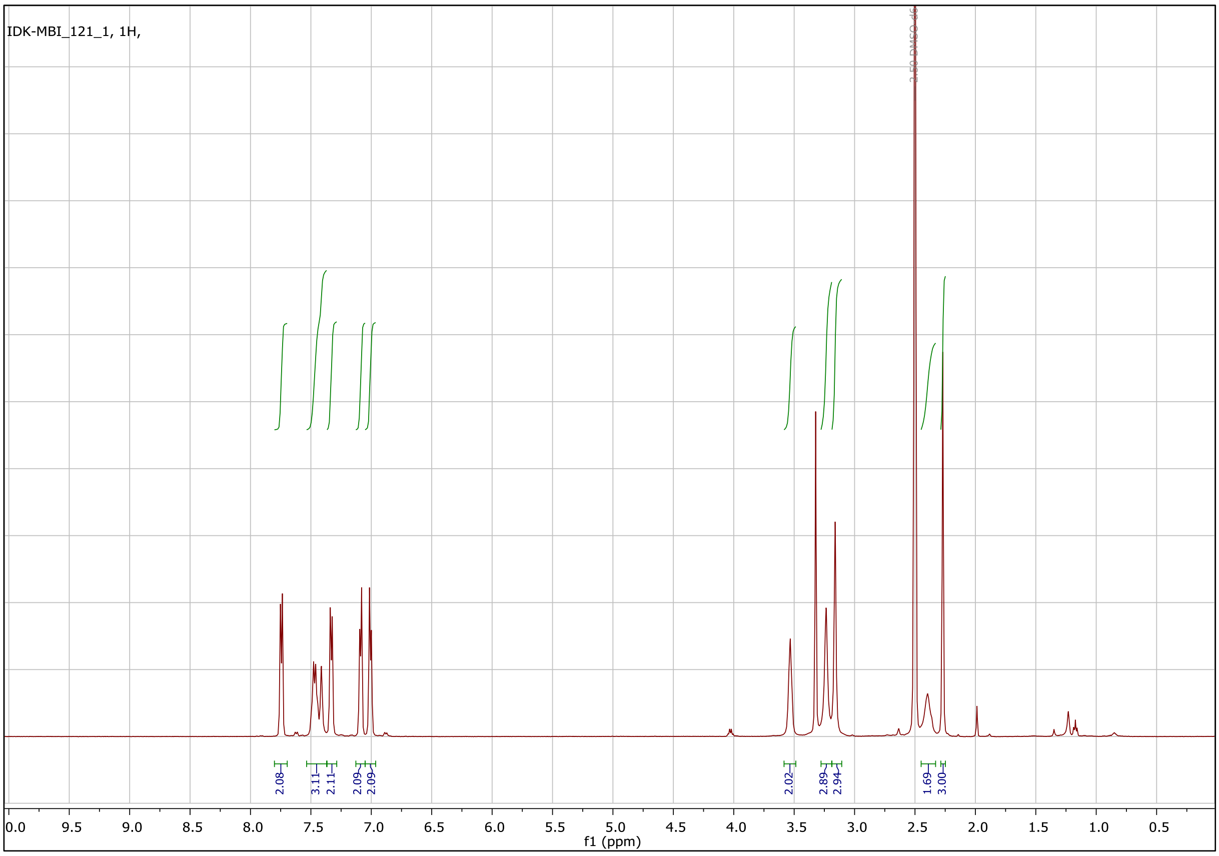

^{1}H NMR (300 MHz, DMSO-d_6) δ 7.74 (d, *J* = 7.9 Hz, 2H), 7.54 – 7.37 (m, 3H), 7.33 (d, *J* = 7.9 Hz, 2H), 7.09 (d, *J* = 7.7 Hz, 2H), 7.01 (d, *J* = 7.7 Hz, 2H), 3.53 (t, *J* = 6.2 Hz, 2H), 3.29 – 3.20 (s, 3H), 3.20 – 3.08 (s, 3H) 2.45 – 2.33 (m, 2H), 2.27 (s, 3H).

IDK12195

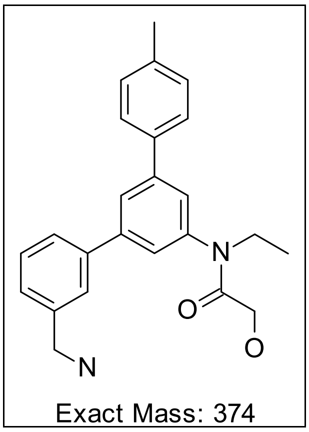

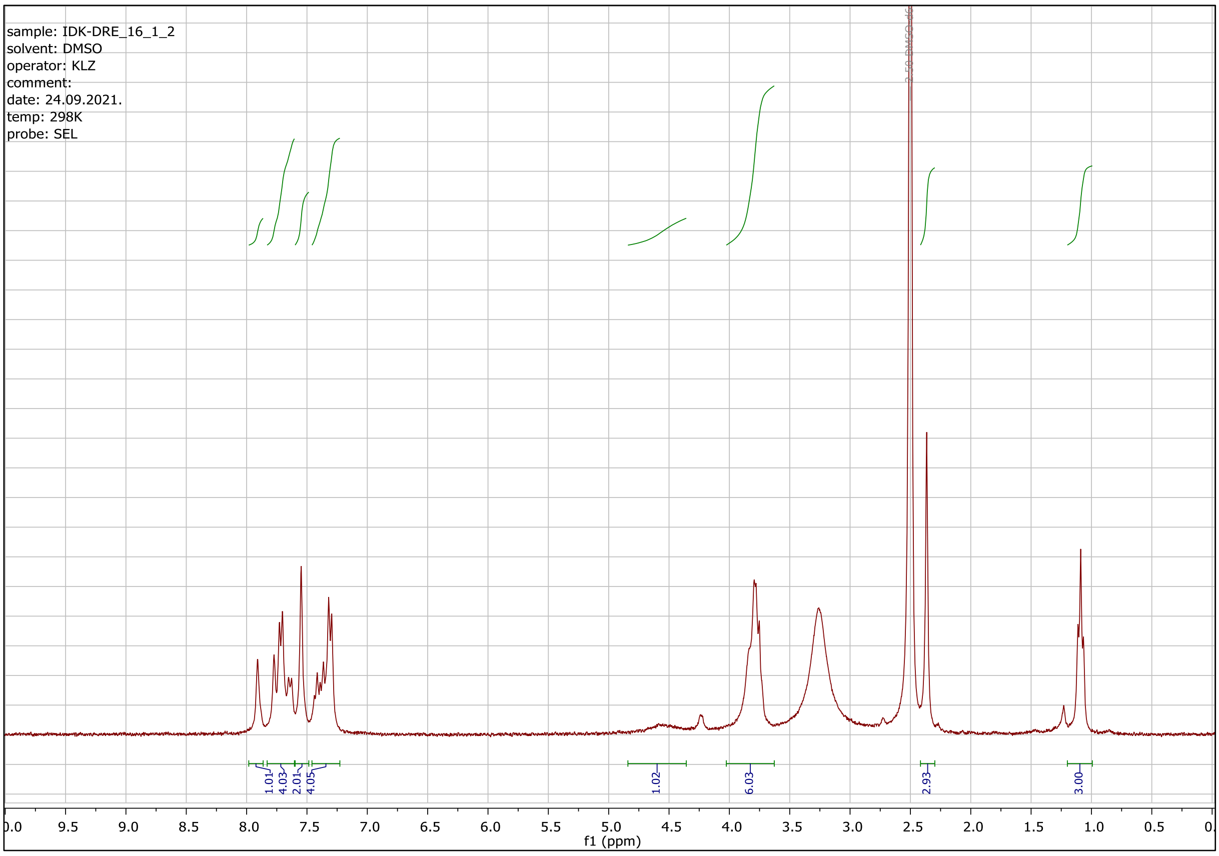

^{1}H NMR (300 MHz, DMSO-d_6) δ 7.91 (s, 1H), 7.83 – 7.60 (m, 4H), 7.55 (s, 2H), 7.46 – 7.23 (m, 4H), 4.58 (br.s, 1H), 4.03 - 3.63 (m, 6H), 2.37 (s, 3H), 1.09 (t, J = 7.1 Hz, 3H).
