# Supplementary material for: De novo generation of multi-target compounds using deep generative chemistry
Source: Nat Commun. 2024 May 6;15:3636. doi: 10.1038/s41467-024-47120-y (PMC11074339; doi:10.1038/s41467-024-47120-y)
Supplement: Supplementary file 7 — Reporting Summary [file 41467_2024_47120_MOESM7_ESM.pdf]

nature portfolio

Corresponding author(s): Trey Ideker

Last updated by author(s): 2/12/2024

Reporting Summary

Nature Portfolio wishes to improve the reproducibility of the work that we publish. This form provides structure for consistency and transparency in reporting. For further information on Nature Portfolio policies, see our Editorial Policies and the Editorial Policy Checklist.

Statistics

For all statistical analyses, confirm that the following items are present in the figure legend, table legend, main text, or Methods section.

n/a	Confirmed	
☐	☒	The exact sample size (*n*) for each experimental group/condition, given as a discrete number and unit of measurement
☐	☒	A statement on whether measurements were taken from distinct samples or whether the same sample was measured repeatedly
☐	☒	The statistical test(s) used AND whether they are one- or two-sided<br>*Only common tests should be described solely by name; describe more complex techniques in the Methods section.*
☒	☐	A description of all covariates tested
☒	☐	A description of any assumptions or corrections, such as tests of normality and adjustment for multiple comparisons
☐	☒	A full description of the statistical parameters including central tendency (e.g. means) or other basic estimates (e.g. regression coefficient) AND variation (e.g. standard deviation) or associated estimates of uncertainty (e.g. confidence intervals)
☐	☒	For null hypothesis testing, the test statistic (e.g. F, t, r) with confidence intervals, effect sizes, degrees of freedom and P value noted<br>*Give P values as exact values whenever suitable.*
☒	☐	For Bayesian analysis, information on the choice of priors and Markov chain Monte Carlo settings
☒	☐	For hierarchical and complex designs, identification of the appropriate level for tests and full reporting of outcomes
☐	☒	Estimates of effect sizes (e.g. Cohen's d, Pearson's r), indicating how they were calculated

Our web collection on statistics for biologists contains articles on many of the points above.

Software and code

Policy information about availability of computer code

Data collection	ProteinSimple (version 6.1.0) for capillary immunoblotting (https://www.bio-techne.com/brands/proteinsimple).<br>Molecular Devices SpectraMax i3x for cell viability quantification (https://www.moleculardevices.com/products/microplate-readers/multi-mode-readers/spectramax-i3x-readers).<br>Reaction Biology HotSpot Kinase Assay for cell-free kinase activity screening (https://www.reactionbiology.com/assay-protocol-hotspot/).
Data analysis	Polygon for polypharmacology compound design: https://github.com/bpmunson/chemist<br>GuacaMol for reward based reinforcement learning: https://github.com/BenevolentAI/guacamol<br>MOSES for the VAE implementation: https://github.com/molecularsets/moses<br>RDKit for computational chemistry: https://www.rdkit.org/<br>SciKit-learn (version 1.7.0) for machine learning: https://scikit-learn.org/stable/<br>Pytorch (version 1.13.1) for machine learning: https://pytorch.org/<br>Neutcurve (version 0.5.7) for drug response analysis: https://jbloomlab.github.io/neutcurve/<br>Autodock Vina (version 1.1.2) for molecular docking: https://vina.scripps.edu/<br>UCSF Chimera (version 1.16) for molecular docking: https://www.cgl.ucsf.edu/chimera/<br>Plotnine (version 0.7.0) for data visualization: https://plotnine.readthedocs.io/<br>SynergyFinder (version 3.0) for calculating drug combinations effects (https://synergyfinder.fimm.fi)<br>Softmax Pro for Molecular Devices SpectraMax i3x for quantification of cell viablity (https://www.moleculardevices.com/products/microplate-readers/multi-mode-readers/spectramax-i3x-readers).<br>Bioblocks, Inc. for 1H NMR of synthesized compounds (https://bioblocks.com/).

For manuscripts utilizing custom algorithms or software that are central to the research but not yet described in published literature, software must be made available to editors and reviewers. We strongly encourage code deposition in a community repository (e.g. GitHub). See the Nature Portfolio guidelines for submitting code & software for further information.

Data

Policy information about availability of data

All manuscripts must include a data availability statement. This statement should provide the following information, where applicable:

- Accession codes, unique identifiers, or web links for publicly available datasets
- A description of any restrictions on data availability
- For clinical datasets or third party data, please ensure that the statement adheres to our policy

The POLYGON source code is publicly available on Gitub at https://github.com/bpmunson/chemist. All datasets and materials generated in this study are provided in the Supplementary Information/Source Data file or from the corresponding author on reasonable request. A key resource to the POLYGON framework is experimental binding data of small molecule ligands. We use the BindingDB (www.bidningdb.org), ChEMBL 24 database (http://doi.org/10.6019/CHEMBL.database.24.1), and the Pharos (https://pharos.nih.gov/) as a source for this information. Molecule training dataset are available from the GuacaMol package: https://github.com/BenevolentAI/guacamol (https://doi.org/10.1021/acs.jcim.8b00839). Validation data of MEK1 and mTOR synergy under combination therapy across a panel of cancer cell lines was sourced from O'Neil et al.44 (https://doi.org/10.1158/1535-7163.mct-15-0843). For molecular docking sumulations we sourced the receptor protein structures from the Protein Data Bank (https://www.rcsb.org/). The following accession codes were used in this study: 7M0Y [https://doi.org/10.2210/pdb7M0Y/pdb], 3FAP [https://doi.org/10.2210/pdb3FAP/pdb], 7KK4 [https://doi.org/10.2210/pdb7KK4/pd], 3MXF [https://doi.org/10.2210/pdb3MXF/pdb], 6XD3 [https://doi.org/10.2210/pdb6XD3/pdb, 6Z45 [https://doi.org/10.2210/pdb6Z45/pdb], 7NXK [https://doi.org/10.2210/pdb7NXK/pdb], 6RLQ [https://doi.org/10.2210/pdb6RLQ/pdb], 7PCD [https://doi.org/10.2210/pdb7PCD/pdb], 6LVM [https://doi.org/10.2210/pdb6LVM/pdb], and1TL8 [https://doi.org/10.2210/pdb1TL8/pdb].

Research involving human participants, their data, or biological material

Policy information about studies with human participants or human data. See also policy information about sex, gender (identity/presentation), and sexual orientation and race, ethnicity and racism.

Reporting on sex and gender	N/A
Reporting on race, ethnicity, or other socially relevant groupings	N/A
Population characteristics	N/A
Recruitment	N/A
Ethics oversight	N/A

Note that full information on the approval of the study protocol must also be provided in the manuscript.

Field-specific reporting

Please select the one below that is the best fit for your research. If you are not sure, read the appropriate sections before making your selection.

☒ Life sciences ☐ Behavioural & social sciences ☐ Ecological, evolutionary & environmental sciences

For a reference copy of the document with all sections, see nature.com/documents/nr-reporting-summary-flat.pdf

Life sciences study design

All studies must disclose on these points even when the disclosure is negative.

Sample size	We chose eight molecular scaffolds to highlight in the embedding of the chemical space as a balance between highlighting chemical diversity and interpretability. To validate the specific compound-dual-target activities predicted by POLYGON we filtered the BindingDB to compounds that had activity profiles measured against two and only two protein structures, this resulted in 109,811 unique compounds. We chose ten pairs of protein targets to generate compound structures against with POLYGON to facilitate a broader interpretation of the generalizability of POLYGON across different proteins and different protein classes. The number of compounds synthesized in this study was balanced between maximizing the nominal amount and minimizing the individual reaction steps used across all molecules. The result was 32 compounds synthesized. Designing against 10 pairs of protein targets and synthesizing 32 molecules is often in excess of similar papers presenting the generation of chemical structures with machine learning techniques [Luo et al., Nat. Commun., 8(1), 573, 2017; Moret et al. Nat. Commun. 14, 1-12, 2023); Kalemati et al., Nat. Commun., 14(1), 1–12, 2023) (Ye et al., Nat. Commun., 12(1), 6775, 2021].
Data exclusions	Samples from the capillary western blot analysis were excluded if no band was detected in any of the primary antibody wells (e.g. GAPDH, ERK, pERK).

Replication	We performed dose-response curves in triplicate and all attempts at replication were successful. Capillary western blots were performed in duplicate or triplicate for each compound and target pair, as indicated. Four of the six compounds tested for replication were successful as indicated in Figure 6g.
Randomization	This study did not employ the technique of randomized case-control groups as each test was performed on biological replicates of the same human cancer cell line and there was no group allocation used.
Blinding	Blinding was not applicable to this study as there was no group allocation used.

Reporting for specific materials, systems and methods

We require information from authors about some types of materials, experimental systems and methods used in many studies. Here, indicate whether each material, system or method listed is relevant to your study. If you are not sure if a list item applies to your research, read the appropriate section before selecting a response.

Materials & experimental systems

n/a	Involved in the study	
☐	☒	Antibodies
☐	☒	Eukaryotic cell lines
☒	☐	Palaeontology and archaeology
☒	☐	Animals and other organisms
☒	☐	Clinical data
☒	☐	Dual use research of concern
☒	☐	Plants

Methods

n/a	Involved in the study	
☒	☐	ChIP-seq
☒	☐	Flow cytometry
☒	☐	MRI-based neuroimaging

Antibodies

Antibodies used	p44/42 MAPK (Erk1/2) (Cell Signaling Technology, 9102) (1:150),<br>Phospho-p44/42 MAPK (Erk1/2) (Thr202/Tyr204) (Cell Signaling Technology, 9101) (1:500)<br>p70 S6 Kinase (Cell Signaling Technology, 9202) (1:200)<br>Phospho-p70 S6 Kinase (Thr389) (Cell Signaling Technology, 9205) (1:10)<br>Phospho-AKT (Thr308) (Cell Signaling Technology, 9275) (1:100)<br>Phospho-Chk1 (Ser345) (Cell Signaling Technology, 2348) (1:100)<br>Phospho-MEK1/2 (Ser217/221) (Cell Signaling Technology, 8154) (1:100)<br>GAPDH (14C10) Rabbit mAB (HRP conjugate) (Cell Signaling Technology, 3683) (1:4000)
Validation	Validation of antibodies is provided on the vendor's (Cell Signaling Technology) websites .

Eukaryotic cell lines

Policy information about cell lines and Sex and Gender in Research

Cell line source(s)	Lung cancer A549 cells were sourced from ATCC (CRM-CCL-185).
Authentication	STR testing was performed on the A549 cells.
Mycoplasma contamination	A549 cell line was tested negative for mycoplasma contamination by IDEXXBioanalytics.
Commonly misidentified lines (See ICLAC register)	No commonly misidentified cell lines were used.
