# Supplementary material for: De novo generation of multi-target compounds using deep generative chemistry
Source: Nat Commun. 2024 May 6;15:3636. doi: 10.1038/s41467-024-47120-y (PMC11074339; doi:10.1038/s41467-024-47120-y)
Supplement: Supplementary file 5 — Supplementary Data 2 [file 41467_2024_47120_MOESM5_ESM.pdf]

Supplementary dataset 2: Synthetic routes of IDK compound

Synthetic Route Tree
(see following table for information on each reaction step)

Starting Material | Intermediates | IDK Compounds

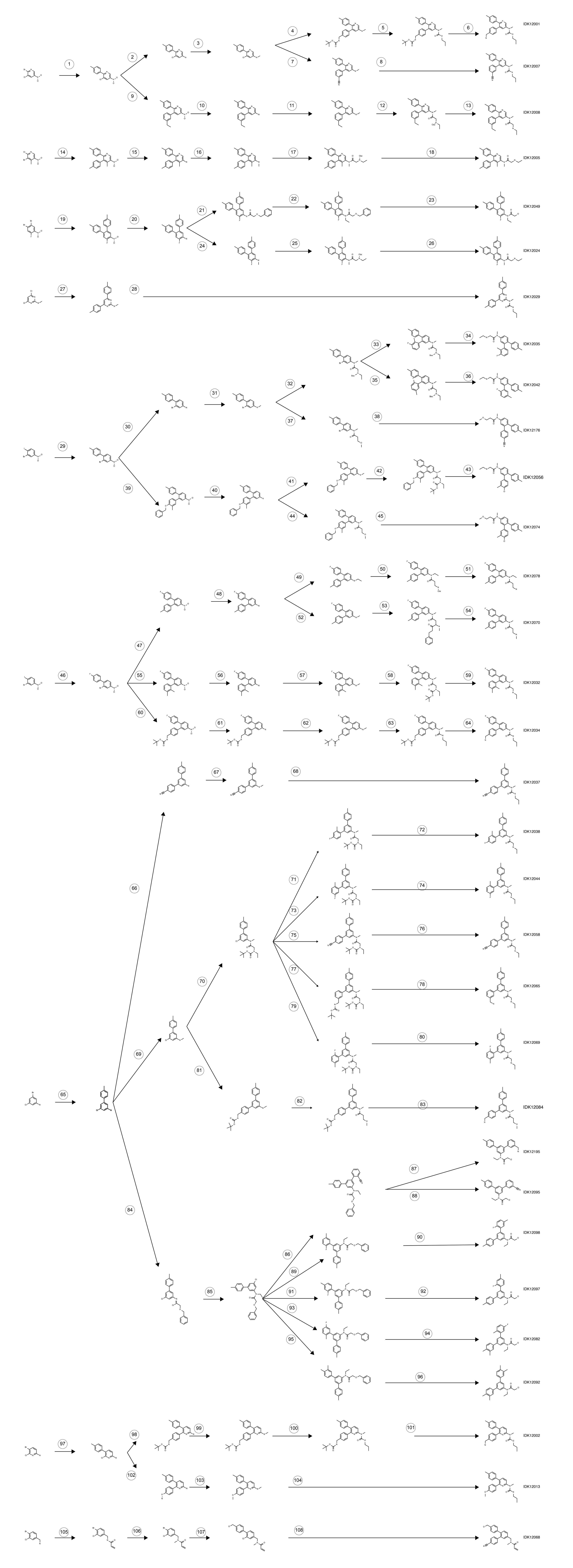

IDK Compound Synthetic Routes
(see following table for information on each reaction step)

Starting Material

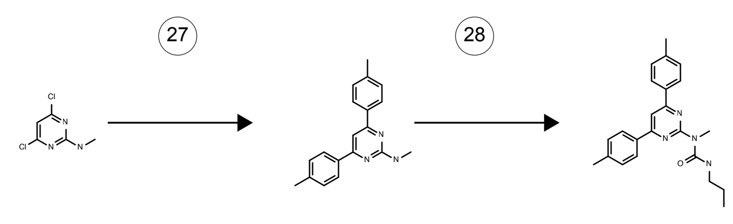

IDK12029

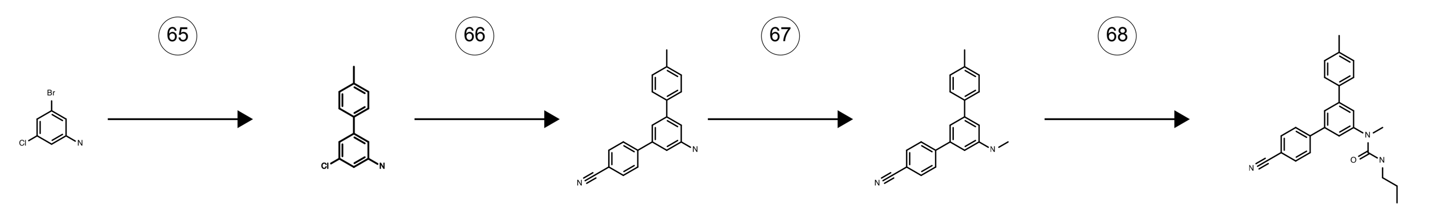

IDK12037

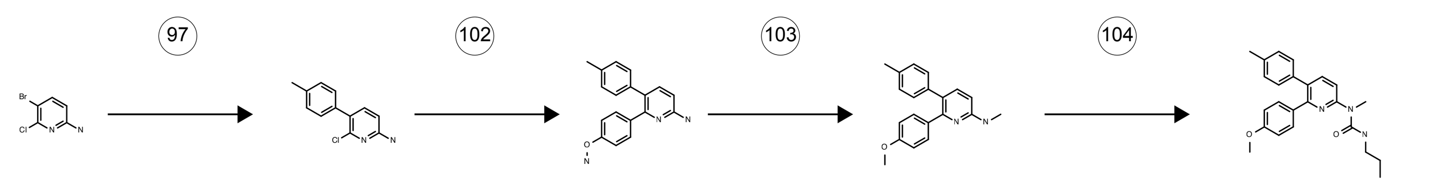

IDK12013

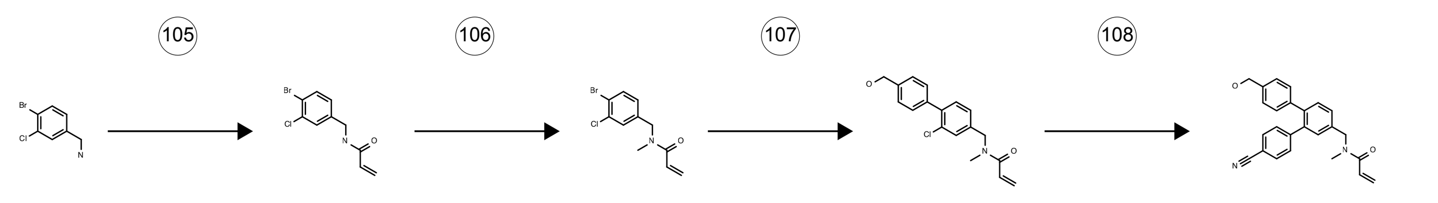

IDK12068

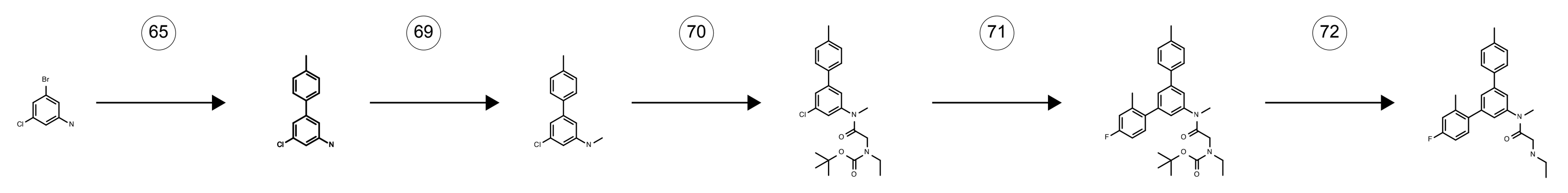

IDK12038

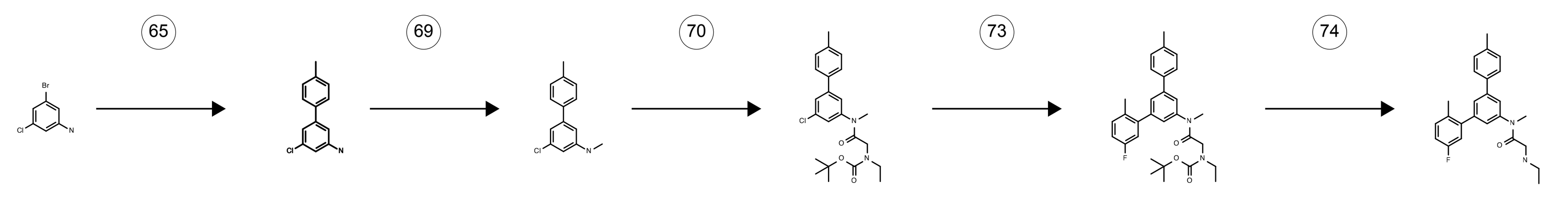

IDK12044

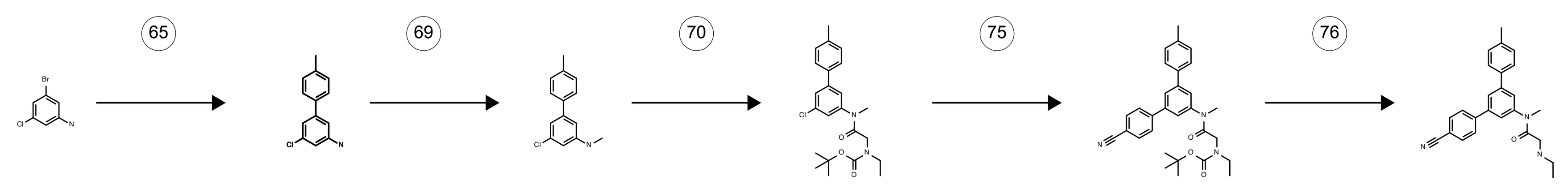

IDK12058

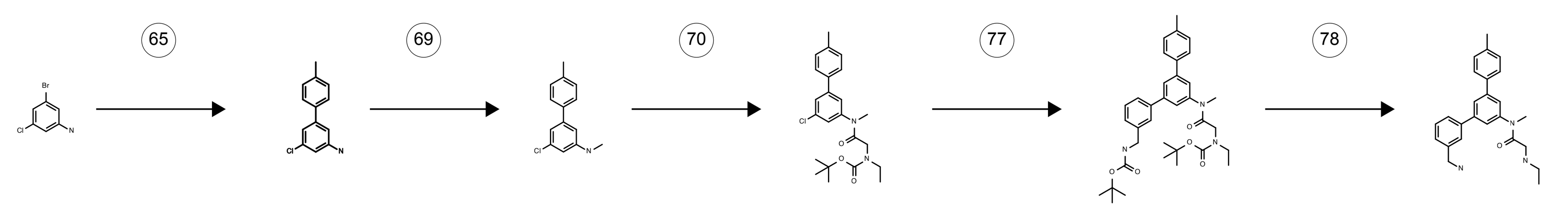

IDK12065

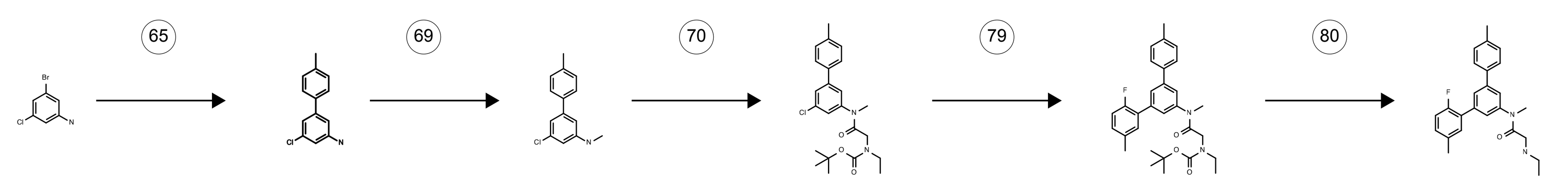

IDK12069

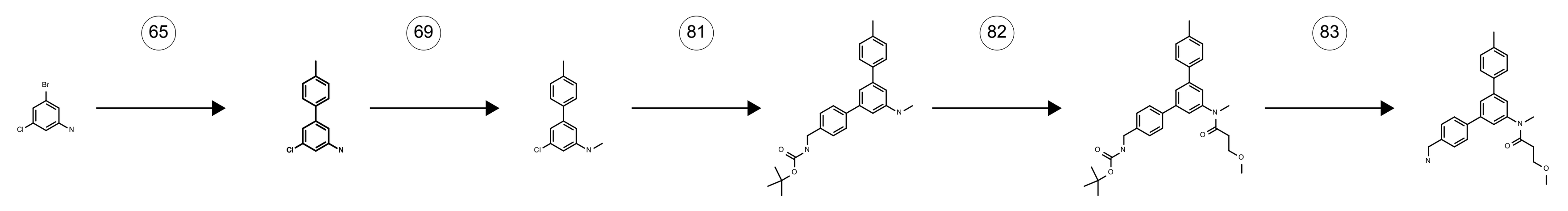

IDK12084

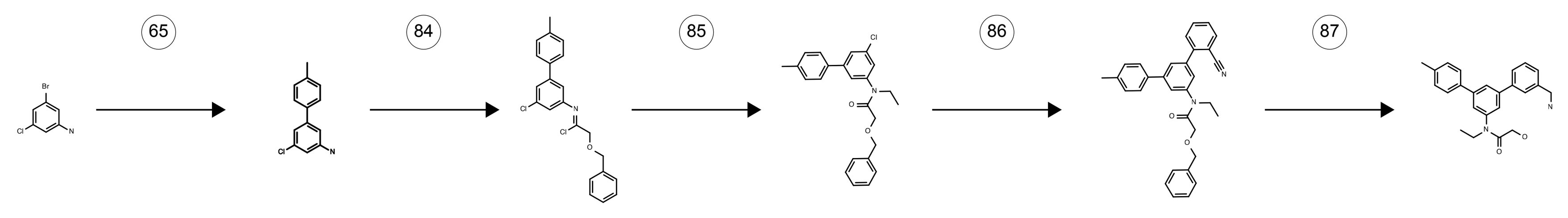

IDK12195

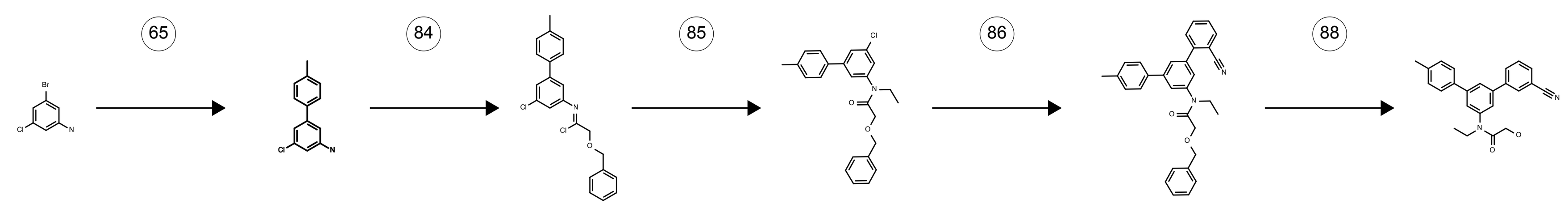

IDK12095

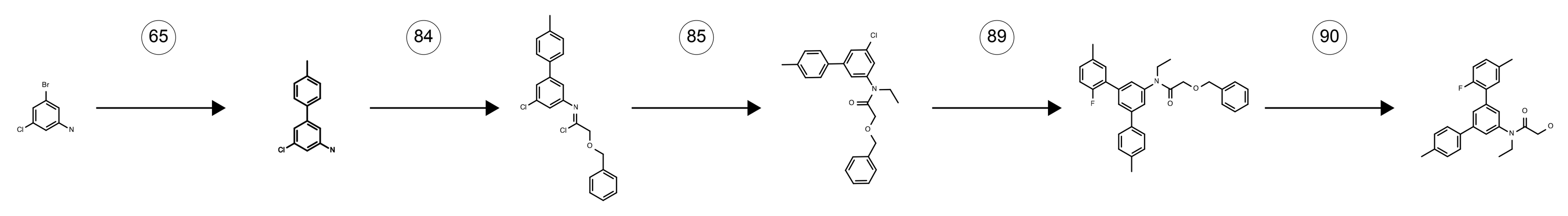

IDK12098

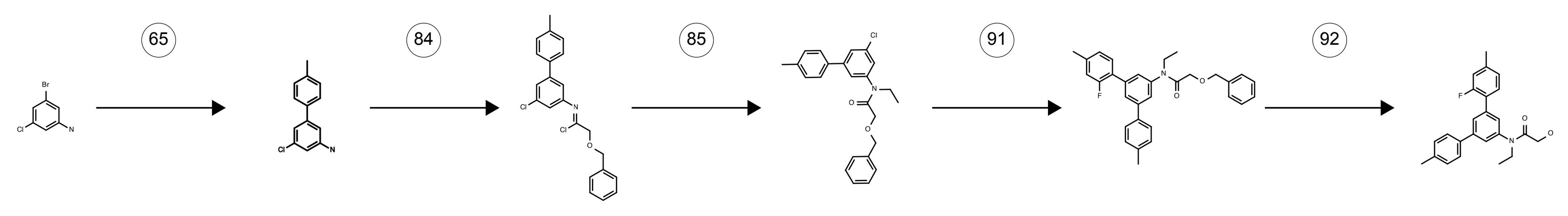

IDK12097

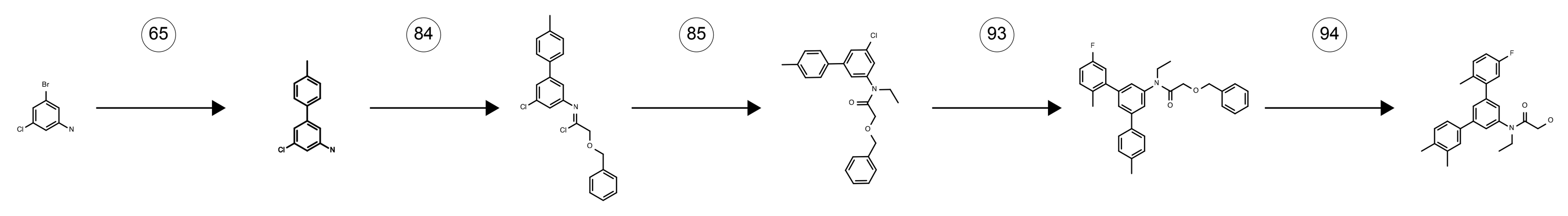

IDK12082

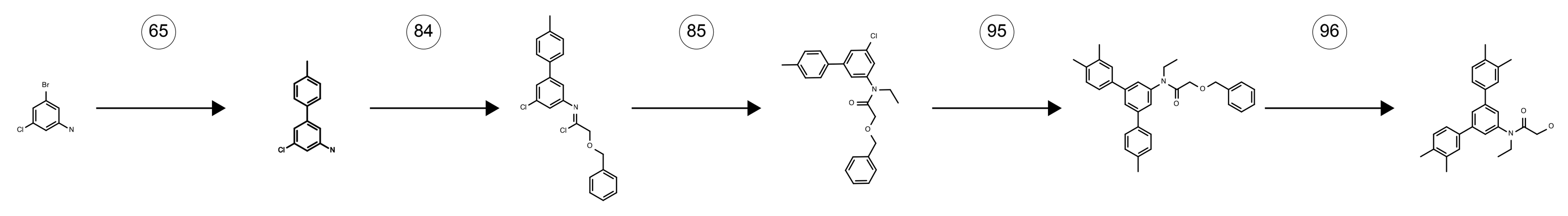

IDK12092

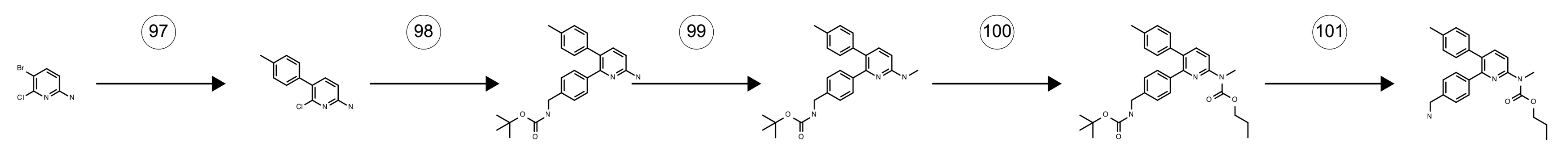

IDK12002

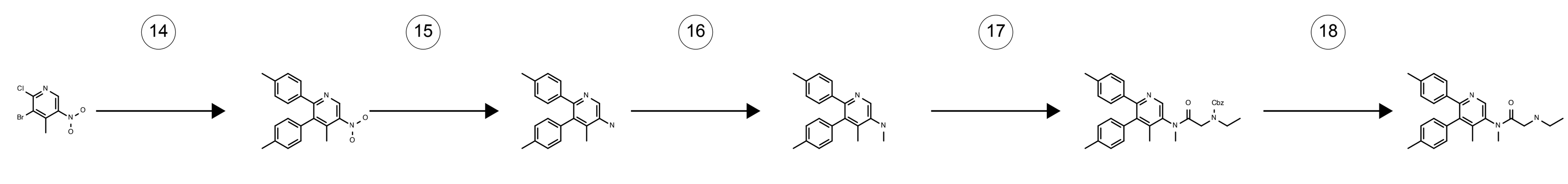

IDK12005

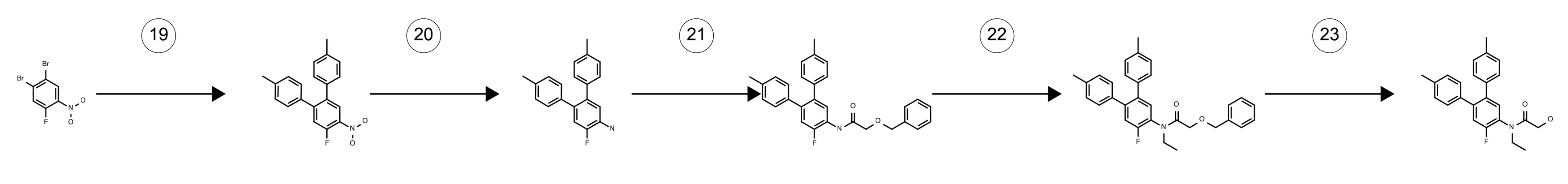

IDK12049

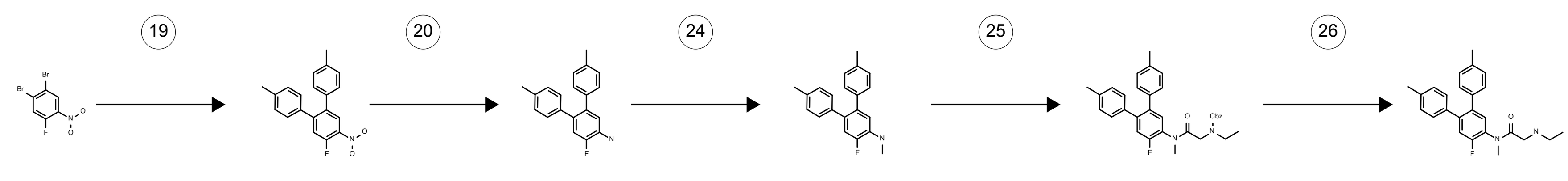

IDK12024

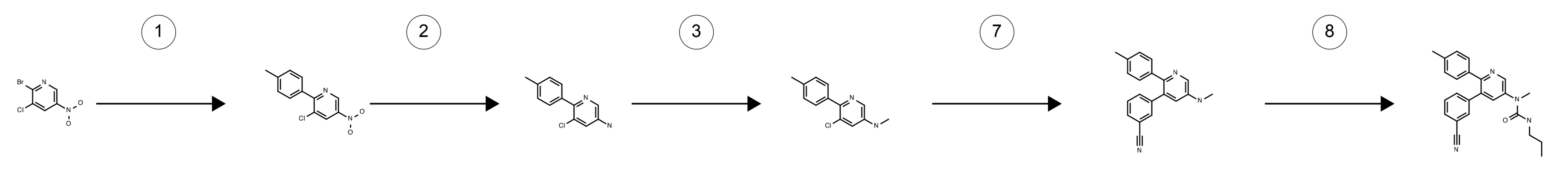

IDK12007

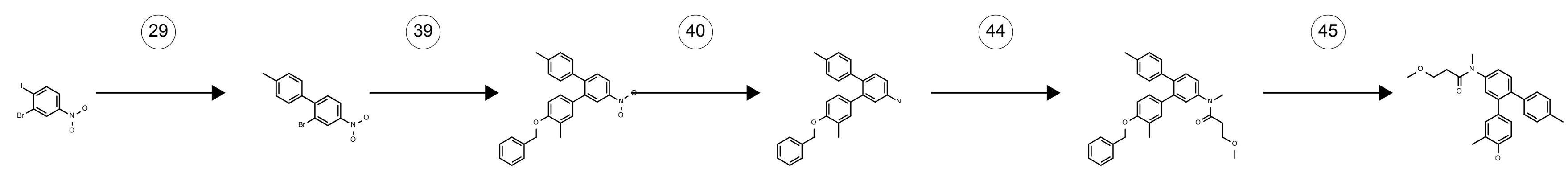

IDK12074

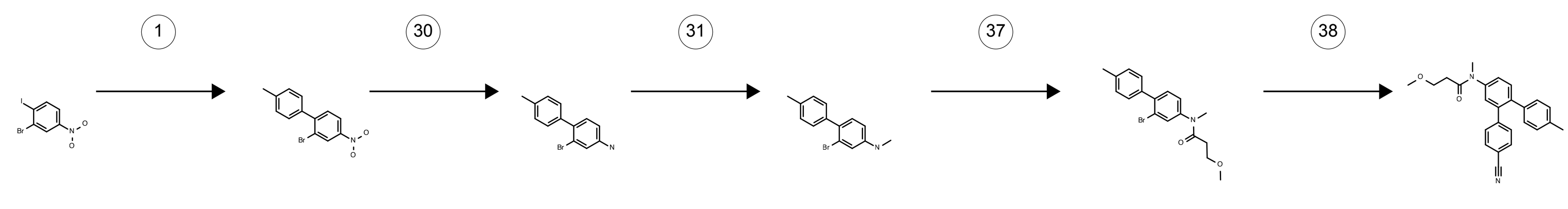

IDK12176

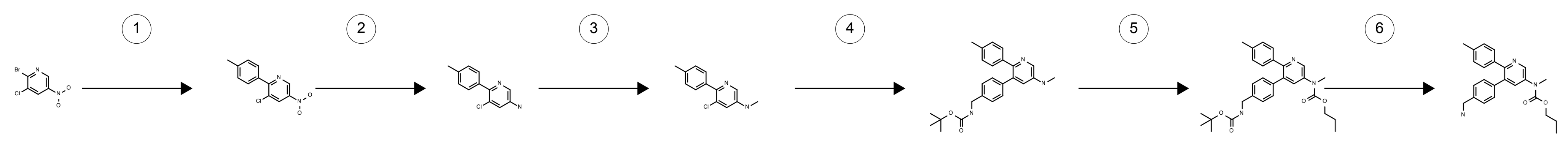

IDK12001

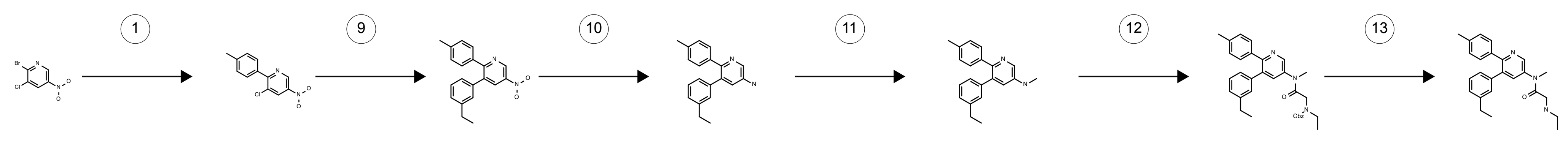

IDK12008

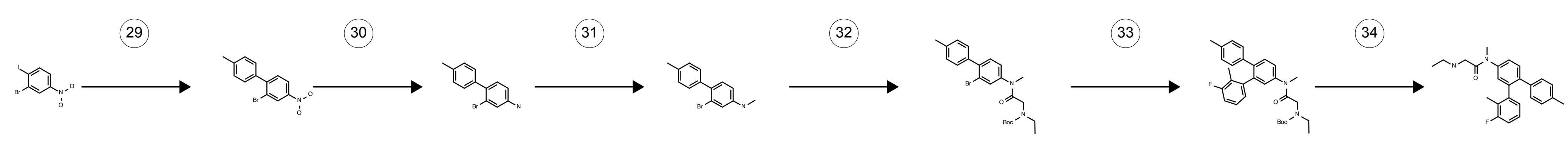

IDK12035

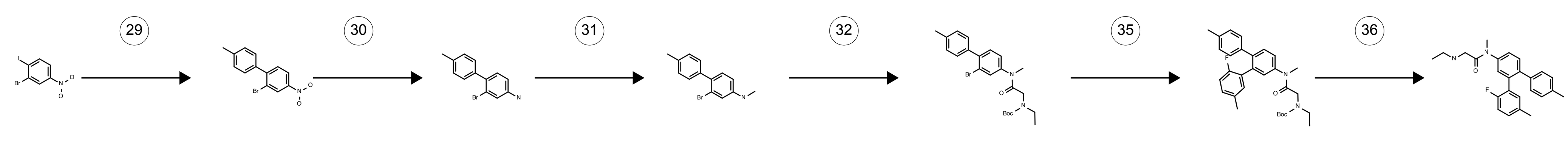

IDK12042

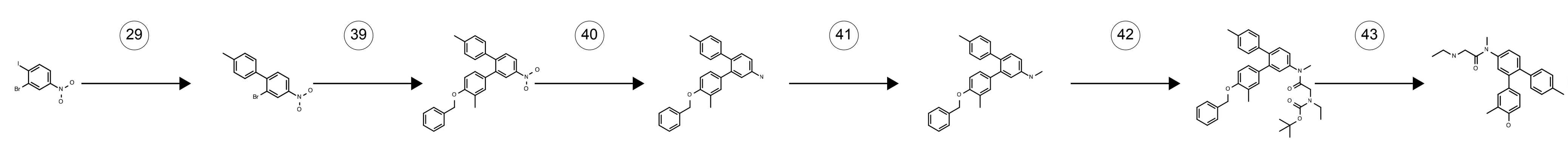

IDK12056

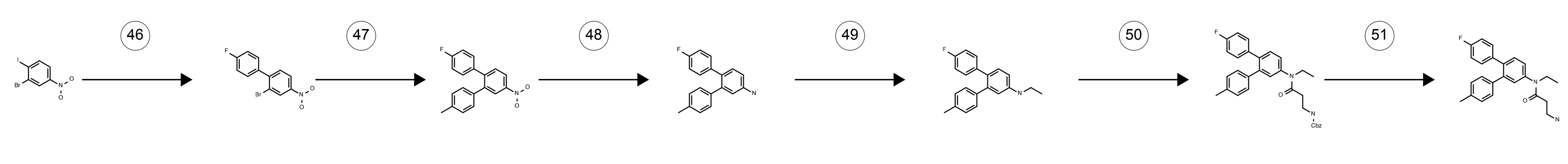

IDK12078

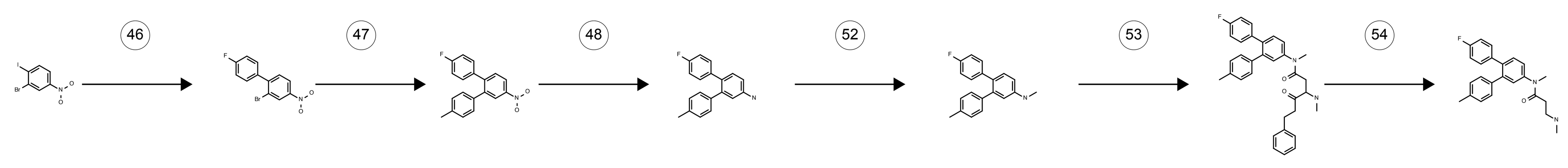

IDK12070

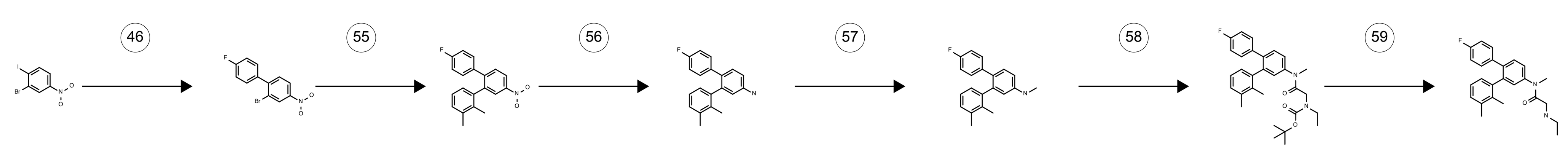

IDK12032

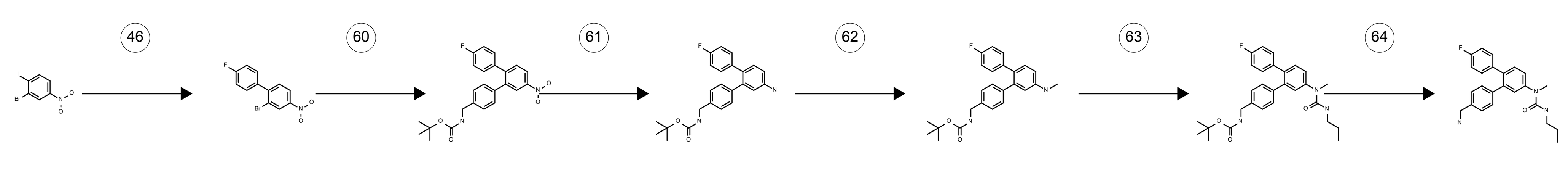

IDK12034

RXN #	Reference		Reaction				Profile	Result			Notebook Ref
	Nr.	type	scale	reagent(s)	T (°C)	time	(**N,M,W,G**)	amount	Y %	LCMS(%)	
**1**		G	1.5 g	*p*-tolylboronic acic, Pd(dppf)Cl2, 2M K3PO4, 1,4-dioxane	65	45 min	G	1.216 g	77	100	IDK-ILN_8
**2**		G	600 mg	Fe, AcOH	RT	3 h	G	482 mg	91	100	IDK-RBL_42
**3**		G	480 mg	1. **3**., NaOMe (2 M, 5 eq.), paraformaldehyde (2 eq.), MeOH<br>2. NaBH4 (1 eq.)	RT<br>65<br>65	16 h<br>2 h<br>2 h	G	457 mg	89	96	IDK-RBL_44
**4**		G	125 mg	4-((N-BOC-AMINO)METHYL)PHENYLBORONIC ACID (1.2 eq), RuPhos Pd G3 (0.05 eq.), Na2CO3 (3 eq.), 1,4-dioxane	80	2 h	G	170 mg	78	100	IDK-RBL_41
**5**		G	115 mg	Triphosgene, 1-Propanol, TEA, DCM	RT<br>80	17 h<br>2 h	G	28 mg	25	97	IDK-RBL_48
**6**											
9		G	450 mg	boronic acid (1.5 eq.), Pd(PPh3)4, 2M Na2CO3, 1,4-dioxane	90	9 h	G	512 mg	88	98	IDK-ILN_9
10		G	465 mg	H2, Pd/C, MeOH	RT	2 h	G	402 mg	95	99	IDK-ILN_12
11		G	361 mg	1. NaOMe, MeOH<br>2. paraformaldehyde<br>3. NaBH4	RT<br>RT<br>60<br>60	30 min<br>16.5 h<br>1.5 h<br>1 h	G	340 mg	83<br>9	99<br>95	IDK-ILN_15
12		G	160 mg	**IM-1**, EDC HCl, DMAP, DCM	RT	84 h	G	215 mg	78	98	IDK-ILN_17
13		G	190 mg	H2, Pd/C, MeOH	RT	2 h	G	106 mg	75	99	IDK-ILN_21
7		G	150 mg<br>(IDK-RBL_44)	3-CYANOPHENYLBORONIC ACID (1.2 eq), RuPhos Pd G3 (0.05 eq.), Na2CO3 (3 eq.), 1,4-dioxane	80	2 h	G	170 mg	88	97	IDK-RBL_47
**8**		G	85 mg	Pr-NCO, DCE	RT<br>50	1 d<br>3 h	G	90 mg	75	98	IDK-MBI_63
14		G	350 mg	4-tolylboronic acid, Pd(dppf) Cl2, 2M K3PO4, 1,4-dioxane	80	1.5 h	G	388 mg	87	100	IDK-ILN_13
15		G	366 mg	H2, Pd/C, MeOH	RT	2 h	G	316 mg	95	100	IDK-ILN_19
16		G	288 mg	1. NaOMe, MeOH<br>2. paraformaldehyde<br><br>3. NaBH4	RT<br>RT<br>60<br>60	30 min<br>20 h<br>3 h<br>1.5 h	G	255 mg	84	100	IDK-ILN_23
17		G	100 mg	**IM-1**, T3P, TEA, EtOAc<br>+ T3P. TEA<br>+ T3P, TEA<br>+ **IM-1**, T3P, TEA	RT	18 h<br>21 h<br>6 h<br>16 h	G				IDK-ILN_27
18		G	80 mg	H2, Pd/C, EtOAc<br>+aq HCl	RT (sonic)	3 h	G	45 mg	63	100	IDK-MBI_64
19		G	500 mg	4-TOLYLBORONIC ACID (3 eq), Pd(dppf)Cl2 (0.1 eq.), Na_2CO_3 (3 eq.), 1,4-dioxane	80	5 h	G	490 mg	91	98	IDK-RBL_14_1
20		G	760 mg	H_2, Pd/C , MeOH	RT	4 h	G	660 mg	95	95	IDK-RBL_18
21		G	200 mg	Benzyloxyacetic acid (1.1 eq), HATU (1.2 eq.), TEA (3 eq.), DCM	RT	45.5 h	G	196 mg	65	100	IDK-RBL_21_1
22		G	170 mg	1. NaH 60wt% ,THF<br>2. EtI	0<br>0-10	30 min<br>110 h	G	45 mg	25	100	IDK-RBL_23_1
23		G	45 mg	H_2, Pd/C , MeOH	RT	4.5 h	G	21 mg	58	98	IDK-RBL_28
24		G	345 mg	1. **3**., NaOMe (2 M, 5 eq.), paraformaldehyde (2 eq.), MeOH<br><br>2. NaBH4 (1 eq.)	RT<br>65<br>60	5 h<br>18 h<br>5 h	G	190 mg	52	100	IDK-RBL_30_1
25		G	165 mg	IM-1, EDC HCl, DMAP, DCM	RT	135.5 h	G	210 mg	74	87	IDK-RBL_31_1
26		G	187 mg	H_2, Pd/C, MeOH	RT	2 h	G	_2: 56 mg	40	99	IDK-RBL_38
27		G	200 mg	boronic acid (3 eq), Pd(dppf)Cl2, K3PO4 (4 eq), 1,4-dioxane	80	1d	G	144 mg	35	99	IDK-MBI_10
28		G	100 mg	Pr-NCO, DCE	100	1 d	G	31 mg	24	97	IDK-MBI_19
**29**		G	3.0 g	boronic acid (1 eq), Pd(dppf)Cl2, K3PO4, 1,4-dioxane	RT	5 d	G	_1: 1.15 g	43	98	IDK-MBI_81
**30**		G	1.0 g	Fe, AcOH	RT	1 h	G	937 mg	94	98	IDK-MBI_102
**31**		G	937 mg	1. NaOMe, MeOH<br>2. paraformaldehyde<br>3. NaBH4	RT<br>60<br>60	30 min<br>4 h<br>1 h	G	900 mg	85	97	IDK-MBI_105
32		G	200 mg	IM-2, HATU, DIPEA, DCM	RT	3 d	G	300 mg	89	97	IDK-MBI_111
33		G	100 mg	boronic acid, RuPhos Pd G3, 2M Na2CO3, 1,4-dioxane	80	4 h	G	85 mg	80	87	IDK-MBI_115
34		G	85 mg	HCl in dioxane	RT	2 h	G	28 mg	40	100	IDK-MBI_119
35		G	100 mg	boronic acid, RuPhos Pd G3, 2M Na2CO3, 1,4-dioxane	80	3 h	G	87 mg	82	96	IDK-MBI_116
36		G	87 mg	HCl in dioxane	RT	2 h	G	42 mg	64	98	IDK-MBI_120
37		G	200 mg	3-METHOXYPROPANOYL CHLORIDE, TEA, DCM	RT	1 d	G	175 mg	66	91	IDK-MBI_110
38		G	175 mg	boronic acid, RuPhos Pd G3, 2M Na2CO3, 1,4-dioxane/water	80	4 h	G	150 mg	63	98	IDK-MBI_121
39		G	660 mg	boronic acid (1.2 eq), Pd(dppf)Cl2, K3PO4, 1,4-dioxane/water	80	1 d	G	750 mg	81	93	IDK-MBI_80
40		G	660 mg	Fe, AcOH	RT	1 d	G	500 mg	81	97	IDK-MBI_99
41		G	470 mg	1. NaOMe, MeOH<br>2. paraformaldehyde<br>3. NaBH4	RT<br>60<br>60	30 min<br>4 h<br>1 h	G	340 mg	69	99	IDK-MBI_106
42		G	200 mg	**IM-2**, HATU, DIPEA, DCM	RT	1 d	G	283 mg	96	98	IDK-MBI_112
43		G	140 mg	HCl in dioxane<br>MeOH, H2, Pd-C	RT<br>RT	2 h<br>1 d	G	75 mg	73	98	IDK-MBI_118
44		G	100 mg	3-METHOXYPROPANOYL CHLORIDE, TEA, DCM	RT	2 d	G	102 mg	83	98	IDK-MBI_113
45		G	102 mg	H2, Pd-C, MeOH	RT	4 d	G	37 mg	45	100	IDK-MBI_114
46		G	2.0 g	boronic acid (1 eq), Pd(dppf)Cl2, K3PO4, 1,4-dioxane	RT	2 d	G	1.1 g	61	93	IDK-MBI_22
47		G	1.1 g	boronic acid (1.2 eq), Pd(dppf)Cl2, K3PO4, 1,4-dioxane	80	1 d	G	1.038 g	90	93	IDK-MBI_23
48		G	1 g	H2, Pd/C, MeOH	RT	2 h	G	913 mg	quant.	96	IDK-MBI_25
**49**		G	300 mg	1. NaOMe, MeOH<br>2. acetaldehyde<br>3. NaBH4	RT<br>60<br>60	15 min<br>1 d<br>1 d	G/N<br>(complete)				IDK-MBI_29
	1	A	250 mg<br>(MBI_29_crd)	acetaldehyde, AcOH, NaBH3CN, MeOH	RT	2 d	G/M *	54 mg	19	95	IDK-MBI_34
**50**		G	54 mg	Z-Beta-ALA-OH, EDC, DMAP, DCM<br>+Z-Beta-ALA-OH, EDC, DMAP (twice a day; 0.5 - 0.5 eqs)	RT	5 d	*				IDK-MBI_36
		G	3 mg (IDK-MBI_36 CF	Z-Beta-ALA-OH, EDC, DMAP, DCE	RT<br>50	1 d<br>7 d	*				IDK-MBI_47
		G	108 mg (CRD)	Z-Beta-ALA-OH, HATU, DIPEA, DMF	RT	18 h	G	37 mg	crude	72	IDK-MBI_58
51		G	37 mg (CRD)	H2, Pd/C, EtOAc<br>+aq HCl	RT (sonic)	2 h	G/M	10 mg	22	98	IDK-MBI_65
52		G	240 mg	1. NaOMe, MeOH<br>2. paraformaldehyde<br><br>3. NaBH4	RT<br>RT<br>60<br>60	15 min<br>1 d<br>1 h<br>1 h	G	166 mg	65	88	IDK-MBI_44
53		G	MBI_33<br>(CRD)	**IM-1**, EDC HCl, DMAP, DCE<br>**+IM-1**, EDC HCl, DMAP	60<br>60	18 h<br>4 h	M	11 mg	5	99	IDK-MBI_35
54		G	59 mg	HCl in dioxane	RT	1 h	G	36 mg	71	89	IDK-MBI_69
55		G	250 mg	boronic acid (1.2 eq), Pd(dppf)Cl2, K3PO4, 1,4-dioxane	80	1 d	G	210 mg	77	92	IDK-MBI_32
56		G	200 mg	H2, Pd/C, MeOH	RT	2 h	G	185 mg	quant.	93	IDK-MBI_45
57		G	185 mg	1. NaOMe, MeOH<br>2. paraformaldehyde<br><br>3. NaBH4	RT<br>RT<br>60<br>60	15 min<br>1 d<br>1 h<br>1 h	G	201 mg	quant.	93	IDK-MBI_46
58		G	10 mg	HATU, DIPEA, DMF	RT	1 d	G	71	55	94	IDK-MBI_57
59		G	70 mg	HCl in dioxane	RT	1 h	G	39 mg	63	97	IDK-MBI_66
60		G	250 mg	boronic acid (1.2 eq), Pd(dppf)Cl2, K3PO4, 1,4-dioxane	80	1 d	G	320 mg	75	96	IDK-MBI_31
61		G	320 mg	H2 (1 bar), Pd/C, MeOH	RT	2 h	G	244 mg	82	96	IDK-MBI_37
62		G	200 mg	1. NaOMe, MeOH<br>2. paraformaldehyde<br><br>3. NaBH4	RT<br>RT<br>60<br>60	15 min<br>1 d<br>1 h<br>1 h	G	240 mg	96	93	IDK-MBI_43
63		G	85 mg	Pr-NCO, DCE	RT	16 h	G	46 mg	48	97	IDK-MBI_48
64		G	40 mg	HCl in dioxane	RT	1 h	G	31 mg	89	99	IDK-MBI_55
65		G	500 mg	4-TOLYLBORONIC ACID (1.2 eq), Pd(dppf)Cl2 (0.05 eq.) K3PO4 (3 eq.), 1,4-dioxane	80	3 h	G	455 mg	86	96	IDK-RBL_4_1
66		G	295 mg	4-CYANOPHENYLBORONIC ACID (1.2 eq), RuPhos Pd G3 (0.05 eq.), Na2CO3 (3 eq.), 1,4-dioxane	80	2.5 h	G	225 mg<br>110 mg	58<br>28	99<br>96	IDK-RBL_8_1<br>IDK-RBL_8_2
67		G	220 mg	1. **3**., NaOMe (2 M, 5 eq.), paraformaldehyde (2 eq.), MeOH<br><br>2. NaBH4 (1 eq.)	RT<br>65<br>60	18 h<br>1 h<br>1 h	G	171 mg	74	99	IDK-RBL_13_1
68		G	166 mg	Pr-NCO, DCE	RT	42 h	G	95 mg	44	100	IDK-RBL_15_1
69		G	1.5 g	1. **2**, NaOMe (2 M, 5 eq.)<br>2. paraformaldehyde (2 eq.), MeOH<br>3. NaBH4 (1 eq.)	RT<br>60<br>60	30 min<br>3 h<br>1 h	G	1.21 g	76	97	IDK-ILN_36
70		G	500 mg	1. **IM-2**, HATU, DIPEA, DMF<br>2. **3**	RT	30 min<br>137 h	G	688 mg	76	97	IDK-ILN_40
71		G	90 mg	boronic acid, RuPhos Pd G3, 2M Na2CO3, 1,4-dioxane	80	2 h	G	107 mg	83	96	IDK-ILN_47
72		G	106 mg	HCl in 1,4-dioxane, DCM	RT	2 h	G	63 mg	68	97	IDK-ILN_49
73		G	90 mg	boronic acid, RuPhos Pd G3, 2M Na2CO3, 1,4-dioxane	80	2 h	G	89 mg	84	97	IDK-ILN_48
74		G	89 mg	HCl in 1,4-dioxane, DCM	RT	2 h	G	57 mg	74	98	IDK-ILN_51
75		G	95 mg	boronic acid, RuPhos Pd G3, 2M Na2CO3, 1,4-dioxane	80	2 h	G	81 mg	73	97	IDK-ILN_50
76		G	78 mg	HCl in 1,4-dioxane, DCM	RT	2 h	G	60 mg	89	98	IDK-ILN_52
77		G	100 mg	boronic acid, RuPhos Pd G3, 2M Na2CO3, 1,4-dioxane	80	2 h	G	73 mg	51	TLC: OK	IDK-DRE_13
78		G	73 mg	HCl in 1,4-dioxane	RT	1 h	G	31 mg	55	98	IDK-DRE_17
79		G	90 mg	boronic acid, RuPhos Pd G3, 2M Na2CO3, 1,4-dioxane	80	2 h	G	90 mg	84	97	IDK-MBI_95
80		G	90 mg	HCl in dioxane	RT	1 h	G	73 mg	93	96	IDK-MBI_97
81		G	200 mg	boronic acid, RuPhos Pd G3, Na2CO3, 1,4-dioxane	80	2 h	G	304 mg	80	97	IDK-ILN_34
82		G	140 mg	3-METHOXYPROPANOYL CHLORIDE, TEA, DCM	0 to RT	17 h	G	88 mg	52	91	IDK-ILN_42
83		G	86 mg	HCl in 1,4-dioxane, DCM	RT	2 h	G	21 mg	31	100	IDK-ILN_46
84		G	240 mg	BENZYLOXYACETIC ACID, HATU, DIPEA, DMF	RT	17 h	G	433 mg	99	95	IDK-ILN_39
85		G	380 mg	1. NaH, DMF<br>2. EtI	0<br>0 to RT	20 min<br>1 d	G	215 mg	52	100	IDK-MBI_96
86		G	100 mg	boronic acid, RuPhos Pd G3, 2M Na2CO3, 1,4-dioxane	80	2 h	G	79 mg	67	97	IDK-DRE_10
87		G	79 mg	H2 (1 to 3 bar), Pd-C, MeOH, EtOAc<br>+HCl in EtOAc	RT	3 d<br>4 h	N<br>G / W *	8.9 mg	13	95	IDK-DRE_16
88		G	79 mg	H2 (1 to 3 bar), Pd-C, MeOH, EtOAc<br>+HCl in EtOAc	RT	3 d<br>4 h	N<br>G / W *	24.1 mg	32	100	
89		G	100 mg	boronic acid, RuPhos Pd G3, 2M Na2CO3, 1,4-dioxane	80	2 h	G	56 mg	47	98	IDK-DRE_12
90		G	54 mg	H2, Pd-C, MeOH	RT	2 h	G	29 mg	66	97	IDK-DRE_19
91		G	100 mg	boronic acid, RuPhos Pd G3, 2M Na2CO3, 1,4-dioxane	80	2 h	G	71 mg	60	100	IDK-DRE_11
92		G	63 mg	H2, Pd-C, MeOH	RT	2 h	G	40 mg	73	95	IDK-DRE_18
93		G	100 mg	boronic acid, RuPhos Pd G3, 2M Na2CO3, 1,4-dioxane	80	2 h	G	104 mg	108	91	IDK-DRE_9
94		G	100 mg	H2, Pd-C, MeOH, THF	RT	1 d	G	57 mg	70	99	IDK-DRE_15
95		G	100 mg	boronic acid, RuPhos Pd G3, 2M Na2CO3, 1,4-dioxane	80	2 h	G	118 mg	100	99	IDK-DRE_20
96		G	115 mg	H2, Pd-C, MeOH	RT	1 d	G	56 mg	58	98	IDK-MBI_117
97		G	500 mg	4-TOLYLBORONIC ACID (0.98 eq), Pd(dppf)Cl2 (0.05 eq.) 2 M Na2CO3 (3 eq.), 1,4-dioxane	80	1 h	G	224 mg	42	100	IDK-RBL_53_1
98		G	275 mg	4-((N-BOC-AMINO)METHYL)PHENYLBORONIC ACID (1.2 eq), RuPhos Pd G3 (0.05 eq.) 2 M K3PO4 (3 eq.), toluene:EtOH (4:1)	80	3 h	G	304 mg	64	94	IDK-RBL_79_1
99		G	258 mg	1. 2, NaOMe (2 M, 5 eq.)<br>2. paraformaldehyde (2 eq.), MeOH<br>3. NaBH4 (1 eq.)	RT<br>60<br>60	30 min<br>4 h<br>1 h	G	185 mg	69	94	IDK-RBL_85_1
100		G	140 mg	Triphosgene, 1-Propanol, TEA, DCM	RT	42 h	G	27 mg	16	97	IDK-RBL_88_1
101		G	26 mg	7M HCl in 1,4-dioxane	RT	4 h	G	17 mg	82	97	IDK-RBL_91_1
102		G	210 mg	4-METHOXYPHENYLBORONIC ACID (1.2 eq), RuPhos Pd G3 (0.05 eq.) 2 M K3PO4 (3 eq.), toluene:EtOH (4:1)	80	17 h	G	206 mg	74	100	IDK-RBL_61_1
103		G	370 mg	1. 2, NaOMe (2 M, 5 eq.)<br>2. paraformaldehyde (2 eq.), MeOH<br>3. NaBH4 (1 eq.)	RT<br>60<br>60	30 min<br>10 h<br>1 h	G	230 mg	60	97	IDK-RBL_80_1
104		G	100 mg	Pr-NCO, DCE	RT	6 d	G	30 mg	23	100	IDK-RBL_81
105	1	A	450 mg	acryloyl chloride, TEA, DCM	RT	1 h	G	502 mg	89	68	IDK-FAB_3
106	1	A	502 mg<br>(crd)	MeI, NaH, THF	0 to RT	45 min	G	151 mg	23<br>(two steps)	89	IDK-FAB_4
107	2	A	75 mg	boronic acic (1.2 eq), Pd(dppf)Cl2 (0.05 eq.) K3PO4 (3 eq.), dioxane/water	80	36 h	G	40 mg	48	89	IDK-FAB_9
108	2	A	40 mg	boronic acid (1.2 eq), RuPhos Pd G3 (0.05 eq.), Na2CO3 (3 eq.), dioxane/water	90	8 h	G	30 mg	33	98	IDK-FAB_7

Letter Keys: **E**: exact
A: analog
G: general

Letter Keys: **N**: no rxn
M: multicomp., no major
W: major product, but wrong
G: major product, good
